# Supplementary material for: Endosome and Golgi‐associated degradation (EGAD) of membrane proteins regulates sphingolipid metabolism
Source: EMBO J. 2019 May 27;38(15):e101433. doi: 10.15252/embj.2018101433 (PMC6669922; doi:10.15252/embj.2018101433)
Supplement: Supplementary file 1 — Appendix [file EMBJ-38-e101433-s001.pdf]

Appendix Tables S1-S6 for Endosome and Golgi-associated degradation (EGAD) of membrane proteins regulates sphingolipid metabolism

Appendix Table S1 - related to Figure 1: Genes that display a synthetic sick / lethal phenotype with *vps4Δ* mutants, p. 2 - 4

Appendix Table S2 - related to Figure 1: Gene Ontology Analysis of Macromolecular Complexes, p. 5 - 6

Appendix Table S3 - related to Figure 1: HMMsearch of the Tul1 ring finger domain against the human UniProt reference proteome, p. 7 - 9

Appendix Table S4 - related to Figure 2: Comparative quantitative analysis of protein turnover in *pep4*Δ and *tul1*Δ,*pep4*Δ cells, p. 9 - 74

Appendix Table S5 - related to Figure EV2: Gene Ontology Analysis of Cellular Processes and Cellular Components from 76 proteins with reduced turnover in Dsc mutants, p. 75 - 79

Appendix Table S6. Yeast strains, plasmids and reagents, p. 80 - 85

Appendix Table S1 - related to Figure 1:
Genes that display a synthetic sick / lethal phenotype with *vps4Δ* mutants

systematic name	standard name	Synthetic Lethal (SL) in both replicates	SL in one and synthetic sick in the other replicate
YAL013W	DEP1		+
YAL016W	TPD3	+	
YAL021C	CCR4	+	
YAL044C	GCV3	+	
YBL003C	HTA2		+
YBL007C	SLA1	+	
YBL010C			+
YBL022C	PIM1	+	
YBL025W	RRN10	+	
YBL058W	SHP1	+	
YBR036C	CSG2		+
YBR089C-A	NHP6B		+
YBR095C	RXT2	+	
YBR099C		+	
YBR126C	TPS1	+	
YBR127C	VMA2	+	
YBR171W	SEC66	+	
YBR189W	RPS9B		+
YCL007C			+
YCR044C	PER1	+	
YCR063W	BUD31	+	
YCR081W	SRB8		+
YCR094W	CDC50	+	
YDL053C	PBP4	+	
YDL091C	UBX3	+	
YDL160C	DHH1	+	
YDL185W	VMA1	+	
YDR129C	SAC6	+	
YDR138W	HPR1	+	
YDR159W	SAC3	+	
YDR161W	ACL4		+
YDR162C	NBP2	+	
YDR207C	UME6		+
YDR225W	HTA1	+	
YDR320C	SWA2	+	
YDR359C	EAF1	+	
YDR455C		+	
YDR456W	NHX1	+	
YEL027W	VMA3	+	

YEL042W	GDA1	+	
YER070W	RNR1	+	
YER074W	RPS24A		+
YER122C	GLO3	+	
YER162C	RAD4		+
YER178W	PDA1		+
YFR030W	MET10	+	
YGL007C-A		+	
YGL023C	PIB2	+	
YGL167C	PMR1	+	
YGL168W	HUR1	+	
YGL188C-A			+
YGL200C	EMP24		+
YGL218W		+	
YGR020C	VMA7	+	
YGR036C	CAX4		+
YGR105W	VMA21	+	
YHL011C	PRS3	+	
YHL025W	SNF6	+	
YHR039C-A	VMA10	+	
YHR067W	HTD2	+	
YHR182W		+	
YIL052C	RPL34B	+	
YIL090W	ICE2		+
YJL029C	VPS53	+	
YJL179W	PFD1	+	
YJL200C	ACO2		+
YJR048W	CYC1	+	
YJR105W	ADO1		+
YKL034W	TUL1		+
YKL048C	ELM1		+
YKL055C	OAR1	+	
YKL080W	VMA5	+	
YKL118W		+	
YKL126W	YPK1	+	
YKL212W	SAC1		+
YKR024C	DBP7	+	
YLR235C		+	
YLR239C	LIP2	+	
YLR285W	NNT1	+	
YLR287C		+	
YLR294C			+
YLR296W		+	
YLR300W	EXG1	+	
YLR309C	IMH1		+
YLR312W-A	MRPL15	+	

YLR320W	MMS22	+	
YLR362W	STE11		+
YLR403W	SFP1	+	
YML008C	ERG6		+
YML009W-B		+	
YML094C-A		+	
YML094W	GIM5	+	
YML112W	CTK3	+	
YMR003W	AIM34	+	
YMR015C	ERG5		+
YMR038C	CCS1	+	
YMR123W	PKR1	+	
YMR165C	PAH1	+	
YMR202W	ERG2	+	
YNL064C	YDJ1		+
YNL076W	MKS1	+	
YNL084C	END3		+
YNL138W	SRV2	+	
YNL229C	URE2	+	
YNL236W	SIN4	+	
YNL243W	SLA2	+	
YNL250W	RAD50		+
YNL280C	ERG24	+	
YNL296W			+
YOL001W	PHO80	+	
YOL009C	MDM12	+	
YOL072W	THP1	+	
YOR061W	CKA2	+	
YOR221C	MCT1	+	
YOR331C		+	
YOR332W	VMA4	+	
YPL042C	SSN3		+
YPL148C	PPT2	+	
YPR172W			+
**YPR173C**	**VPS4**	**+ (bait)**	

Appendix Table S2 - related to Figure 1: Gene Ontology Analysis of Macromolecular Complexes

GOID	GO term	Frequency	Genome Frequency	Gene(s)	pvalue_adj	enrichment
**GO:0044695**	**Dsc E3 ubiquitin ligase complex**	**2 out of 119 genes, 1.7%**	**4 of 6433 genes, 0.1%**	**UBX3,TUL1**	**1,01E-02**	**27,03**
**GO:0070390**	**transcription export complex 2**	**2 out of 119 genes, 1.7%**	**4 of 6433 genes, 0.1%**	**SAC3,THP1**	**1,01E-02**	**27,03**
**GO:0016471**	**vacuolar proton-transporting V-type ATPase complex**	**7 out of 119 genes, 5.9%**	**17 of 6433 genes, 0.3%**	**VMA2,VMA1,VMA3,VM**	**1,57E-07**	**22,26**
**GO:0016592**	**mediator complex**	**3 out of 119 genes, 2.5%**	**9 of 6433 genes, 0.1%**	**SRB8,SIN4,SSN3**	**4,80E-03**	**18,02**
**GO:0016272**	**prefoldin complex**	**2 out of 119 genes, 1.7%**	**6 of 6433 genes, 0.1%**	**PFD1,GIM5**	**1,60E-02**	**18,02**
**GO:0016593**	**Cdc73/Paf1 complex**	**2 out of 119 genes, 1.7%**	**7 of 6433 genes, 0.1%**	**CCR4,HPR1**	**1,83E-02**	**15,45**
**GO:0033698**	**Rpd3L complex**	**3 out of 119 genes, 2.5%**	**12 of 6433 genes, 0.2%**	**DEP1,RXT2,UME6**	**8,94E-03**	**13,51**
**GO:0000788**	**nuclear nucleosome**	**2 out of 119 genes, 1.7%**	**9 of 6433 genes, 0.1%**	**HTA2,HTA1**	**2,78E-02**	**12,01**
**GO:0070210**	**Rpd3L-Expanded complex**	**3 out of 119 genes, 2.5%**	**19 of 6433 genes, 0.3%**	**DEP1,RXT2,UME6**	**1,60E-02**	**8,54**
GO:0000307	cyclin-dependent protein kinase holoenzyme complex	2 out of 119 genes, 1.7%	20 of 6433 genes, 0.3%	CTK3,PHO80	9,61E-02	5,41
GO:0031298	replication fork protection complex	2 out of 119 genes, 1.7%	25 of 6433 genes, 0.4%	HTA2,HTA1	1,22E-01	4,32
GO:0000932	P-body	2 out of 119 genes, 1.7%	42 of 6433 genes, 0.7%	CCR4,DHH1	1,95E-01	2,57
GO:0016591	DNA-directed RNA polymerase II, holoenzyme	3 out of 119 genes, 2.5%	75 of 6433 genes, 1.2%	CCR4,HPR1,SIN4	1,65E-01	2,16
GO:0000790	nuclear chromatin	5 out of 119 genes, 4.2%	133 of 6433 genes, 2.1%	DEP1,HTA2,RXT2,UME6,	1,22E-01	2,03
GO:0005643	nuclear pore	2 out of 119 genes, 1.7%	54 of 6433 genes, 0.8%	SAC3,THP1	2,41E-01	2,00
GO:0010494	cytoplasmic stress granule	3 out of 119 genes, 2.5%	89 of 6433 genes, 1.4%	VMA2,PBP4,DHH1	1,95E-01	1,82
GO:0022627	cytosolic small ribosomal subunit	2 out of 119 genes, 1.7%	65 of 6433 genes, 1.0%	RPS9B,RPS24A	2,58E-01	1,66
GO:0005667	transcription factor complex	2 out of 119 genes, 1.7%	81 of 6433 genes, 1.3%	RRN10,SIN4	2,73E-01	1,33
GO:0030686	90S preribosome	2 out of 119 genes, 1.7%	90 of 6433 genes, 1.4%	RPS9B,CKA2	2,74E-01	1,20
GO:0022626	cytosolic ribosome	3 out of 119 genes, 2.5%	180 of 6433 genes, 2.8%	RPS9B,RPS24A,RPL34B	2,58E-01	0,90
GO:0015934	large ribosomal subunit	2 out of 119 genes, 1.7%	150 of 6433 genes, 2.3%	RPL34B,MRPL15	2,69E-01	0,72

Notes:
Choosing large ribosomal subunit 0.72078431372549 over intracellular ribonucleoprotein complex. Parent-process has a higher p-value.
Choosing ribosome 0.605701103971 over intracellular ribonucleoprotein complex. Parent-process has a higher p-value.
Choosing Sin3-type complex 10.1360294117647 over histone deacetylase complex. Parent-process has a higher p-value.
Choosing P-body 2.57422969187675 over intracellular ribonucleoprotein complex. Parent-process has a higher p-value.
Choosing 90S preribosome 1.20130718954248 over preribosome,intracellular ribonucleoprotein complex. Parent-process has a higher p-value.
Choosing cytosolic ribosome 0.900980392156863 over ribosome,intracellular ribonucleoprotein complex. Parent-process has a higher p-value.
Choosing preribosome 0.59080681452909 over intracellular ribonucleoprotein complex. Parent-process has a higher p-value.
Choosing nuclear chromatin 2.03228659885007 over chromatin. Parent-process has a higher p-value.
Choosing Rpd3L complex 13.5147058823529 over Sin3-type complex,histone deacetylase complex. Parent-process has a higher p-value.
Choosing Rpd3L-Expanded complex 8.53560371517028 over histone deacetylase complex. Parent-process has a higher p-value.
Choosing Dsc E3 ubiquitin ligase complex 27.0294117647059 over ubiquitin ligase complex. Parent-process has a higher p-value.
Choosing cytosolic small ribosomal subunit 1.66334841628959 over small ribosomal subunit,intracellular ribonucleoprotein complex. Parent-process has a higher p-value.

Choosing small ribosomal subunit 1.08117647058824 over intracellular ribonucleoprotein complex. Parent-process has a higher p-value.
Choosing cytoplasmic stress granule 1.82220753469927 over intracellular ribonucleoprotein complex. Parent-process has a higher p-value.
Choosing Cdc73/Paf1 complex 15.4453781512605 over transcription elongation factor complex. Parent-process has a higher p-value.
large ribosomal subunit is the highest level.
cyclin-dependent protein kinase holoenzyme complex is the highest level.
DNA-directed RNA polymerase II, holoenzyme is the highest level.
transcription factor complex is the highest level.
P-body is the highest level.
nuclear pore is the highest level.
90S preribosome is the highest level.
cytosolic ribosome is the highest level.
mediator complex is the highest level.
nuclear chromatin is the highest level.
Rpd3L complex is the highest level.
Rpd3L-Expanded complex is the highest level.
vacuolar proton-transporting V-type ATPase complex is the highest level.
Dsc E3 ubiquitin ligase complex is the highest level.
cytosolic small ribosomal subunit is the highest level.
prefoldin complex is the highest level.
nuclear nucleosome is the highest level.
cytoplasmic stress granule is the highest level.
transcription export complex 2 is the highest level.
replication fork protection complex is the highest level.
Cdc73/Paf1 complex is the highest level.

Appendix Table S3 - related to Figure 1: HMMsearch of the Tul1 ring finger domain against the human UniProt reference proteome. The hitlist is sorted by E-value (of the full sequence) with similarites to Tul1 RING domain

UniProt Acc.	E-value	score	Gene Name	description
Q9H9V4	5,8E-13	48,6	RNF122	RING finger protein 122
Q9Y225	6,7E-12	45,2	RNF24	RING finger protein 24
Q7L0R7	8,1E-12	45	RNF44	RING finger protein 44
Q96MT1	1,4E-11	44,2	RNF145	RING finger protein 145
Q9H0F5	2,5E-11	43,4	RNF38	E3 ubiquitin-protein ligase RNF38
Q9UKV5	4,5E-11	42,6	AMFR	E3 ubiquitin-protein ligase AMFR
Q86TM6	5,8E-11	42,2	SYVN1	E3 ubiquitin-protein ligase synoviolin
Q6ZSG1	7,9E-11	41,8	RNF165	E3 ubiquitin-protein ligase RNF165
Q9P0P0	9,8E-11	41,5	RNF181	E3 ubiquitin-protein ligase RNF181
Q9H0A6	9,8E-11	41,5	RNF32	RING finger protein 32
Q8WU17	1E-10	41,4	RNF139	E3 ubiquitin-protein ligase RNF139
P78317	4,3E-10	39,4	RNF4	E3 ubiquitin-protein ligase RNF4
Q6ZNA4	8,2E-10	38,5	RNF111	E3 ubiquitin-protein ligase Arkadia
Q9Y3C5	0,000000001	38,2	RNF11	RING finger protein 11
Q9Y4L5	1,1E-09	38,1	RNF115	E3 ubiquitin-protein ligase RNF115
Q7Z569	2,1E-09	37,2	BRAP	BRCA1-associated protein
Q9NVW2	3,4E-09	36,6	RLIM	E3 ubiquitin-protein ligase RLIM
Q9H6Y7	3,5E-09	36,5	RNF167	E3 ubiquitin-protein ligase RNF167
Q9Y252	9,6E-09	35,1	RNF6	E3 ubiquitin-protein ligase RNF6
Q9BV68	0,000000011	34,9	RNF126	E3 ubiquitin-protein ligase RNF126
O43567	0,000000012	34,8	RNF13	E3 ubiquitin-protein ligase RNF13
Q86Y13	0,000000015	34,5	DZIP3	E3 ubiquitin-protein ligase DZIP3
Q86XS8	0,000000026	33,7	RNF130	E3 ubiquitin-protein ligase RNF130
Q8NC42	0,000000054	32,7	RNF149	E3 ubiquitin-protein ligase RNF149
Q9ULT6	0,000000057	32,6	ZNRF3	E3 ubiquitin-protein ligase ZNRF3
Q17RB8	0,000000071	32,3	LONRF1	LON peptidase N-terminal domain and RING finger protein 1
Q8N4F7	0,000000073	32,3	RNF175	RING finger protein 175
Q9ULK6	0,000000083	32,1	RNF150	RING finger protein 150
O60683	0,000000093	32	PEX10	Peroxisome biogenesis factor 10

Q9H920	0,000000095	31,9	RNF121	RING finger protein 121
Q8WWF5	0,0000001	31,8	ZNRF4	E3 ubiquitin-protein ligase ZNRF4
O76064	0,00000015	31,3	RNF8	E3 ubiquitin-protein ligase RNF8
Q9Y6U7	0,0000004	29,9	RNF215	RING finger protein 215
Q8ND25	0,00000043	29,8	ZNRF1	E3 ubiquitin-protein ligase ZNRF1
O43164	0,00000046	29,7	PJA2	E3 ubiquitin-protein ligase Praja-2
P53804	0,00000059	29,4	TTC3	E3 ubiquitin-protein ligase TTC3
Q96BH1	0,00000059	29,4	RNF25	E3 ubiquitin-protein ligase RNF25
Q9BWF2	0,00000061	29,3	TRAIP	E3 ubiquitin-protein ligase TRAIP
Q8WVZ7	0,00000063	29,3	RNF133	E3 ubiquitin-protein ligase RNF133
O00237	0,00000082	28,9	RNF103	E3 ubiquitin-protein ligase RNF103
Q8NHG8	0,00000085	28,9	ZNRF2	E3 ubiquitin-protein ligase ZNRF2
O94822	0,00000095	28,7	LTN1	E3 ubiquitin-protein ligase listerin
Q8N7C7	0,0000011	28,5	RNF148	RING finger protein 148
Q68DV7	0,0000018	27,8	RNF43	E3 ubiquitin-protein ligase RNF43
P62877	0,000002	27,7	RBX1	E3 ubiquitin-protein ligase RBX1
Q8TEB7	0,0000023	27,5	RNF128	E3 ubiquitin-protein ligase RNF128
Q496Y0	0,0000026	27,3	LONRF3	LON peptidase N-terminal domain and RING finger protein 3
Q96PM5	0,0000028	27,2	RCHY1	RING finger and CHY zinc finger domain-containing protein 1
Q13702	0,0000028	27,2	RAPSN	43 kDa receptor-associated protein of the synapse
Q3KNV8	0,0000068	26	PCGF3	Polycomb group RING finger protein 3
Q2KHN1	0,0000089	25,6	RNF151	RING finger protein 151
Q9H270	0,000011	25,3	VPS11	Vacuolar protein sorting-associated protein 11 homolog
Q9NS56	0,000012	25,2	TOPORS	E3 ubiquitin-protein ligase Topors
Q99942	0,000015	24,9	RNF5	E3 ubiquitin-protein ligase RNF5
Q8NG27	0,000016	24,8	PJA1	E3 ubiquitin-protein ligase Praja-1
Q13064	0,000016	24,8	MKRN3	Probable E3 ubiquitin-protein ligase makorin-3
Q96GF1	0,000016	24,8	RNF185	E3 ubiquitin-protein ligase RNF185
Q8TDB6	0,000023	24,3	DTX3L	E3 ubiquitin-protein ligase DTX3L
Q13434	0,000028	24	MKRN4P	Putative E3 ubiquitin-protein ligase makorin-4
Q9UHC7	0,000038	23,6	MKRN1	E3 ubiquitin-protein ligase makorin-1
Q8N9I9	0,00004	23,5	DTX3	Probable E3 ubiquitin-protein ligase DTX3
Q6PCD5	0,000058	23	RFWD3	E3 ubiquitin-protein ligase RFWD3

P22681	0,00007	22,7	CBL	E3 ubiquitin-protein ligase CBL
Q13191	0,000075	22,7	CBLB	E3 ubiquitin-protein ligase CBL-B
Q96EQ8	0,000075	22,6	RNF125	E3 ubiquitin-protein ligase RNF125
Q8TEC5	0,000079	22,6	SH3RF2	E3 ubiquitin-protein ligase SH3RF2
Q7Z6J0	0,000087	22,4	SH3RF1	E3 ubiquitin-protein ligase SH3RF1
Q9BRZ2	0,00011	22,1	TRIM56	E3 ubiquitin-protein ligase TRIM56
Q8NEG5	0,00015	21,7	ZSWIM2	E3 ubiquitin-protein ligase ZSWIM2
Q5M7Z0	0,00016	21,6	RNFT1	E3 ubiquitin-protein ligase RNFT1
Q9UBF6	0,00017	21,5	RNF7	RING-box protein 2
Q8N3P4	0,00019	21,4	VPS8	Vacuolar protein sorting-associated protein 8 homolog
Q96EP1	0,0002	21,3	CHFR	E3 ubiquitin-protein ligase CHFR
A0A1B0GVX0	0,00022	21,2	LOC101929989	Uncharacterized protein
Q1L5Z9	0,00023	21,1	LONRF2	LON peptidase N-terminal domain and RING finger protein 2
Q9H4P4	0,0003	20,7	RNF41	E3 ubiquitin-protein ligase NRDP1
Q8WVD5	0,00049	20	RNF141	RING finger protein 141
Q8N448	0,0005	20	LNX2	Ligand of Numb protein X 2
Q9UPQ7	0,00054	19,9	PDZRN3	E3 ubiquitin-protein ligase PDZRN3
Q9C040	0,00082	19,3	TRIM2	Tripartite motif-containing protein 2
Q86Y01	0,00086	19,2	DTX1	E3 ubiquitin-protein ligase DTX1
Q9NS91	0,001	19	RAD18	E3 ubiquitin-protein ligase RAD18

Appendix Table S4 - related to Figure 2: Comparative quantitative analysis of protein turnover in *pep4*∆ and *tul1*∆,*pep4*∆ cells

marked in bold are predicted membrane transmembrane proteins with [H/L(*tul1*∆,*pep4*∆ / H/L(*pep4*∆)] >2

(PEP4* = non-functional N-terminal fragment of Pep4 comprising amino acids 1-137 that is expressed from the the *PEP4* locus in *pep4*∆ strains made as described in Ammerer et al. 1986.)

Accession	Protein	*tul1Δ pep4*∆ Abundance (H/L)	*pep4*∆ Abundance (H/L)	[H/L(*tul1*Δ, *pep4*∆)] / [H/L(*pep4*∆)]	*tul1Δ pep4*∆ abundances count (light)	*pep4*∆ abundances count (light)	*tul1Δ pep4*∆ abundances count (heavy)	*pep4*∆ abundances count (heavy)
YCR020W-B	HTL1	0,984	0,01	98,400	1	1	1	0
YLR419W	YLR419W	0,959	0,01	95,900	4	1	4	0
YDR181C	SAS4	0,958	0,01	95,800	1	1	1	0
**YPR113W**	**PIS1**	**0,936**	**0,01**	**93,600**	**3**	**1**	**3**	**0**
YPL045W	VPS16	0,934	0,01	93,400	1	1	1	0
**YOR093C**	**YOR093C**	**0,923**	**0,01**	**92,300**	**1**	**1**	**1**	**0**
YBL066C	SEF1	0,898	0,01	89,800	4	1	3	0
YDL240W	LRG1	0,855	0,01	85,500	3	2	3	0
**YLR018C**	**POM34**	**0,854**	**0,01**	**85,400**	**4**	**1**	**3**	**0**
YIL156W	UBP7	0,852	0,01	85,200	1	1	1	0
**YLR020C**	**YEH2**	**0,847**	**0,01**	**84,700**	**2**	**1**	**2**	**0**
YGR171C	MSM1	0,841	0,01	84,100	1	1	1	0
YMR060C	SAM37	0,84	0,01	84,000	1	1	1	0
YMR275C	BUL1	0,827	0,01	82,700	3	1	3	0
**YMR162C**	**DNF3**	**0,818**	**0,01**	**81,800**	**4**	**2**	**5**	**0**
**YPR128C**	**ANT1**	**0,809**	**0,01**	**80,900**	**2**	**2**	**2**	**0**
**YJL134W**	**LCB3**	**0,801**	**0,01**	**80,100**	**3**	**1**	**3**	**0**
YOL081W	IRA2	0,795	0,01	79,500	1	1	1	0
YHR154W	RTT107	0,794	0,01	79,400	6	2	6	0
YIL079C	AIR1	0,782	0,01	78,200	1	2	1	0
YOR274W	MOD5	0,775	0,01	77,500	4	2	3	0
YBR264C	YPT10	0,769	0,01	76,900	1	1	1	0
**YLR205C**	**HMX1**	**0,756**	**0,01**	**75,600**	**2**	**1**	**3**	**0**
**YPL227C**	**ALG5**	**0,751**	**0,01**	**75,100**	**2**	**1**	**2**	**0**
YOR054C	VHS3	0,74	0,01	74,000	2	1	2	0
YKR010C	TOF2	0,736	0,01	73,600	4	2	4	0
**YGL080W**	**FMP37**	**0,73**	**0,01**	**73,000**	**5**	**1**	**5**	**0**
**YLR350W**	**ORM2**	**0,729**	**0,01**	**72,900**	**4**	**2**	**4**	**0**
YJR140C	HIR3	0,708	0,01	70,800	2	1	2	0
**YNL065W**	**AQR1**	**0,708**	**0,01**	**70,800**	**2**	**1**	**2**	**0**
YNL236W	SIN4	0,677	0,01	67,700	2	1	2	0
YHR172W	SPC97	0,666	0,01	66,600	4	2	4	0

YNL234W	YNL234W	0,663	0,01	66,300	2	1	2	0
**YCR037C**	**PHO87**	**0,635**	**0,01**	**63,500**	**2**	**1**	**2**	**0**
YBL093C	ROX3	0,626	0,01	62,600	1	1	1	0
**YOR307C**	**SLY41**	**0,615**	**0,01**	**61,500**	**2**	**1**	**2**	**0**
YHR082C	KSP1	0,601	0,01	60,100	3	2	4	0
**YMR071C**	**TVP18**	**0,577**	**0,01**	**57,700**	**1**	**1**	**1**	**0**
**YGR281W**	**YOR1**	**0,562**	**0,01**	**56,200**	**2**	**1**	**2**	**0**
YGR133W	PEX4	0,503	0,01	50,300	1	1	1	0
YHR165C	PRP8	0,456	0,01	45,600	2	1	2	0
**YDR326C**	**YSP2**	**0,439**	**0,01**	**43,900**	**2**	**1**	**2**	**0**
YDL104C	QRI7	0,432	0,01	43,200	2	1	1	0
YOR035C	SHE4	0,414	0,01	41,400	2	1	3	0
YDR208W	MSS4	0,384	0,01	38,400	3	1	2	0
YIL144W	TID3	0,895	0,025	35,800	3	1	3	1
**YLR056W**	**ERG3**	**0,291**	**0,01**	**29,100**	**1**	**1**	**1**	**0**
YML091C	RPM2	0,278	0,01	27,800	2	1	2	0
**YLR066W**	**SPC3**	**0,851**	**0,111**	**7,667**	**5**	**1**	**5**	**4**
YOR188W	MSB1	0,667	0,088	7,580	7	1	5	3
YPL112C	**PEX25**	0,851	0,152	5,599	1	1	1	2
YCL032W	STE50	0,652	0,117	5,573	1	1	2	1
**YBR230C**	**OM14**	**0,889**	**0,168**	**5,292**	**5**	**2**	**5**	**2**
**YPL221W**	**FLC1**	**0,746**	**0,148**	**5,041**	**5**	**2**	**5**	**1**
YJR043C	POL32	0,973	0,259	3,757	1	1	2	2
**YER145C**	**FTR1**	**0,702**	**0,195**	**3,600**	**2**	**1**	**3**	**2**
**YDR470C**	**UGO1**	**0,484**	**0,147**	**3,293**	**2**	**2**	**2**	**2**
**YDL012C**	**YDL012C**	**0,714**	**0,217**	**3,290**	**2**	**1**	**2**	**2**
**YDR093W**	**DNF2**	**0,986**	**0,327**	**3,015**	**3**	**1**	**3**	**2**
**YJL062W-A**	**COA3**	**1**	**0,333**	**3,003**	**4**	**2**	**3**	**2**
YGR296W	YRF1-3	0,754	0,261	2,889	7	4	4	3
YGR283C	YGR283C	0,812	0,285	2,849	4	2	4	2
**YOR016C**	**ERP4**	**0,938**	**0,331**	**2,834**	**5**	**4**	**4**	**4**
YDL030W	PRP9	0,982	0,352	2,790	1	2	1	1
YOR278W	HEM4	0,933	0,356	2,621	2	2	1	2
**YGL160W**	**AIM14**	**0,903**	**0,349**	**2,587**	**6**	**3**	**6**	**3**
**YIL048W**	**NEO1**	**0,918**	**0,383**	**2,397**	**5**	**3**	**5**	**2**
YGL029W	CGR1	0,853	0,365	2,337	6	2	5	4
YGR198W	YPP1	0,848	0,376	2,255	3	2	3	1
**YAL026C**	**DRS2**	**0,974**	**0,434**	**2,244**	**5**	**1**	**4**	**3**
YDR283C	GCN2	0,847	0,38	2,229	2	1	2	2

YPR036W-A	YPR036W-A	0,143	0,068	2,103	1	1	1	2
YBL046W	PSY4	0,807	0,386	2,091	1	1	1	2
**YDL193W**	**NUS1**	**0,946**	**0,455**	**2,079**	**4**	**1**	**3**	**2**
YDR415C	YDR415C	0,925	0,447	2,069	1	1	1	2
YCL049C	YCL049C	0,79	0,386	2,047	1	3	1	3
YMR305C	SCW10	0,735	0,368	1,997	8	13	8	13
YGL257C	MNT2	0,904	0,46	1,965	1	1	1	1
YPR101W	SNT309	0,657	0,335	1,961	1	1	1	1
YBL037W	APL3	0,985	0,537	1,834	1	2	3	2
YPL135W	ISU1	0,294	0,162	1,815	4	4	3	4
YLL015W	BPT1	0,732	0,41	1,785	5	3	5	2
YHL023C	NPR3	0,676	0,38	1,779	1	1	1	2
YDR475C	JIP4	0,738	0,417	1,770	1	1	3	2
YBR162C	TOS1	0,658	0,373	1,764	5	9	6	10
YDL159W	STE7	0,724	0,413	1,753	2	1	2	2
YLL022C	HIF1	0,889	0,508	1,750	7	6	6	7
YHR207C	SET5	0,97	0,555	1,748	5	7	4	5
YHR122W	YHR122W	0,842	0,486	1,733	3	3	2	3
YKL062W	MSN4	0,515	0,298	1,728	5	8	6	10
YHR023W	MYO1	0,8	0,471	1,699	9	8	12	7
YKL099C	UTP11	0,816	0,483	1,689	11	8	11	9
YDR195W	REF2	0,914	0,55	1,662	3	5	4	5
YKR092C	SRP40	0,786	0,476	1,651	11	5	12	5
YDR512C	EMI1	0,861	0,524	1,643	1	1	1	1
YIL016W	SNL1	0,923	0,565	1,634	3	2	1	2
YKR100C	SKG1	0,98	0,6	1,633	5	5	4	5
YDR371W	CTS2	0,899	0,552	1,629	1	1	1	1
YGR279C	SCW4	0,585	0,36	1,625	16	13	16	11
YML114C	TAF8	0,843	0,531	1,588	3	6	2	7
YDR182W	CDC1	0,865	0,55	1,573	3	1	3	1
YGL252C	RTG2	0,85	0,544	1,563	8	9	7	9
YJL159W	HSP150	0,488	0,315	1,549	4	4	3	3
YJR132W	NMD5	0,788	0,515	1,530	4	2	4	2
YDR162C	NBP2	0,63	0,412	1,529	3	5	3	5
YNL101W	AVT4	0,895	0,588	1,522	2	2	2	2
YCL044C	MGR1	0,92	0,605	1,521	5	2	5	3
YIL127C	RRT14	0,677	0,446	1,518	12	4	9	4
YNL139C	THO2	0,795	0,528	1,506	15	7	17	6
YHR102W	KIC1	0,842	0,562	1,498	2	1	3	1

YCL025C	AGP1	0,818	0,546	1,498	1	1	1	1
YPR025C	CCL1	0,835	0,559	1,494	4	3	4	2
YNL039W	BDP1	0,844	0,566	1,491	5	6	7	7
YJL049W	YJL049W	0,882	0,601	1,468	2	3	5	5
YER001W	MNN1	0,616	0,421	1,463	2	2	2	3
YLR262C	YPT6	0,951	0,65	1,463	4	3	5	3
YKL105C	YKL105C	0,807	0,553	1,459	2	2	2	1
YDR505C	PSP1	0,947	0,653	1,450	3	7	2	7
YER100W	UBC6	0,336	0,233	1,442	1	1	1	1
YNL254C	RTC4	0,797	0,553	1,441	3	1	3	1
YNL192W	CHS1	0,616	0,428	1,439	7	5	5	5
YNL048W	ALG11	0,972	0,677	1,436	6	4	5	3
YHR045W	YHR045W	0,795	0,555	1,432	3	3	3	3
YBR179C	FZO1	0,93	0,651	1,429	9	7	8	8
YNL191W	DUG3	0,641	0,451	1,421	2	1	2	1
YKR036C	CAF4	0,814	0,573	1,421	1	1	1	1
YDR170C	SEC7	0,919	0,649	1,416	10	9	10	10
YLR189C	ATG26	0,89	0,63	1,413	1	1	1	1
YBR108W	AIM3	0,959	0,679	1,412	4	8	4	8
YPR008W	HAA1	0,9	0,64	1,406	5	8	4	8
YER128W	YER128W	0,961	0,688	1,397	3	6	3	5
YMR015C	ERG5	0,356	0,256	1,391	11	5	9	6
YDR096W	GIS1	0,753	0,542	1,389	2	5	1	7
YLR095C	IOC2	0,892	0,644	1,385	5	3	5	4
YJL173C	RFA3	0,632	0,457	1,383	3	4	3	4
YBR102C	EXO84	0,822	0,595	1,382	3	7	5	4
YOR276W	CAF20	0,83	0,601	1,381	13	16	15	13
YML071C	COG8	0,737	0,534	1,380	5	1	4	3
YNL221C	POP1	0,829	0,601	1,379	1	2	1	2
YHR057C	CPR2	0,631	0,458	1,378	3	3	2	3
YDR457W	TOM1	0,91	0,666	1,366	7	2	6	4
YPL074W	YTA6	0,78	0,571	1,366	2	1	3	3
YJL158C	CIS3	0,867	0,635	1,365	7	3	7	4
YNL261W	ORC5	0,804	0,589	1,365	1	1	1	1
YKR018C	YKR018C	0,954	0,701	1,361	2	8	2	6
YJR033C	RAV1	0,996	0,736	1,353	2	2	1	1
YFR031C	SMC2	0,962	0,715	1,345	5	5	4	2
YBR123C	TFC1	0,871	0,649	1,342	3	3	3	2
YHR206W	SKN7	0,818	0,61	1,341	6	8	4	8

YGR295C	COS6	0,806	0,603	1,337	1	1	1	1
YBR170C	NPL4	0,958	0,717	1,336	3	6	3	7
YBL085W	BOI1	0,998	0,747	1,336	12	14	11	13
YLR378C	SEC61	0,922	0,691	1,334	7	4	5	3
YBL004W	UTP20	0,753	0,565	1,333	10	3	11	5
YMR233W	TRI1	0,794	0,596	1,332	3	3	2	3
YMR054W	STV1	0,846	0,637	1,328	1	2	1	2
YDL045W-A	MRP10	0,815	0,614	1,327	5	4	4	4
YNL288W	CAF40	0,889	0,67	1,327	2	1	2	1
YDR310C	SUM1	0,969	0,733	1,322	14	14	14	13
YER088C	DOT6	0,633	0,479	1,322	7	7	7	8
YOL060C	MAM3	0,652	0,494	1,320	5	1	5	1
YDR449C	UTP6	0,958	0,727	1,318	2	2	2	2
YMR295C	YMR295C	0,767	0,583	1,316	4	5	4	5
YMR255W	GFD1	0,588	0,449	1,310	6	6	5	6
YER083C	GET2	0,839	0,641	1,309	14	4	13	9
YMR076C	PDS5	0,824	0,63	1,308	4	6	5	4
YJL052W	TDH1	0,931	0,712	1,308	8	13	6	12
YIL123W	SIM1	0,546	0,418	1,306	5	9	4	8
YOL141W	PPM2	0,859	0,658	1,305	2	3	1	4
YKL096W	CWP1	0,305	0,234	1,303	7	9	4	10
YDR464W	SPP41	0,592	0,455	1,301	2	5	2	5
YLR356W	ATG33	0,761	0,586	1,299	1	2	1	2
YPL053C	KTR6	0,659	0,508	1,297	8	4	7	4
YKR093W	PTR2	0,557	0,43	1,295	1	1	1	1
YBL023C	MCM2	0,955	0,738	1,294	2	5	1	6
YDR270W	CCC2	0,674	0,521	1,294	2	1	2	1
YPR155C	NCA2	0,793	0,614	1,292	1	2	1	2
YKL186C	MTR2	0,295	0,23	1,283	1	1	2	2
YLR209C	PNP1	0,974	0,761	1,280	5	11	6	11
YEL018W	EAF5	0,888	0,697	1,274	6	5	5	5
YBR188C	NTC20	0,754	0,592	1,274	5	7	3	6
YMR294W	JNM1	0,801	0,629	1,273	5	3	4	4
YBR283C	SSH1	0,78	0,616	1,266	5	3	6	4
YDR097C	MSH6	0,769	0,609	1,263	9	12	8	12
YDR055W	PST1	0,468	0,371	1,261	13	6	13	9
YBR244W	GPX2	0,96	0,762	1,260	2	3	1	4
YMR272C	SCS7	0,559	0,444	1,259	7	3	8	4
YBR242W	YBR242W	0,706	0,561	1,258	6	5	8	6

YER155C	BEM2	0,848	0,675	1,256	14	10	14	9
YDR017C	KCS1	0,861	0,688	1,251	4	8	5	6
YHR161C	YAP1801	0,986	0,789	1,250	5	3	5	5
YNL186W	UBP10	0,768	0,615	1,249	10	14	9	14
YNL049C	SFB2	0,92	0,737	1,248	4	1	5	1
YHR205W	SCH9	0,925	0,743	1,245	9	8	8	7
YDR398W	UTP5	0,773	0,621	1,245	3	5	3	5
YML100W	TSL1	0,954	0,767	1,244	12	12	11	12
YBR069C	TAT1	0,675	0,543	1,243	5	5	5	6
YAL053W	FLC2	0,751	0,605	1,241	4	5	3	5
YPR189W	SKI3	0,882	0,711	1,241	13	11	10	9
YDR392W	SPT3	0,887	0,716	1,239	1	2	1	2
YPL082C	MOT1	0,875	0,707	1,238	6	4	6	2
YLR068W	FYV7	0,788	0,637	1,237	6	7	7	8
YMR148W	OSW5	0,912	0,739	1,234	4	2	3	2
YML115C	VAN1	0,721	0,585	1,232	3	2	2	3
YKR042W	UTH1	0,487	0,396	1,230	16	11	18	9
YOR138C	RUP1	0,768	0,625	1,229	4	3	4	4
YNL253W	TEX1	0,806	0,656	1,229	3	1	4	1
YDR016C	DAD1	0,828	0,674	1,228	1	1	1	1
YER172C	BRR2	0,874	0,712	1,228	5	5	7	6
YIR022W	SEC11	0,877	0,715	1,227	4	2	3	1
YMR296C	LCB1	0,909	0,742	1,225	10	11	10	8
YCR086W	CSM1	0,795	0,649	1,225	1	3	1	3
YDR476C	YDR476C	0,932	0,763	1,221	5	4	4	4
YNL084C	END3	0,968	0,793	1,221	14	14	13	14
YML128C	MSC1	0,936	0,768	1,219	12	17	13	21
YPL031C	PHO85	0,807	0,663	1,217	5	7	5	8
YJL165C	HAL5	0,872	0,717	1,216	4	5	4	5
YNL021W	HDA1	0,701	0,577	1,215	1	1	1	1
YJR133W	XPT1	0,99	0,815	1,215	7	12	5	12
YNL163C	RIA1	0,816	0,672	1,214	16	13	17	9
YMR208W	ERG12	1	0,825	1,212	7	6	8	7
YDR232W	HEM1	0,659	0,544	1,211	16	22	16	18
YIL104C	SHQ1	0,643	0,532	1,209	3	2	4	3
YPR154W	PIN3	0,674	0,558	1,208	2	6	1	6
YDR477W	SNF1	0,916	0,76	1,205	14	15	14	16
YJL183W	MNN11	0,854	0,71	1,203	9	6	7	6
YNR010W	CSE2	0,891	0,741	1,202	3	5	4	6

YPL032C	SVL3	0,93	0,774	1,202	14	8	14	11
YIL035C	CKA1	0,903	0,752	1,201	6	5	5	5
YKL173W	SNU114	0,85	0,709	1,199	6	4	8	5
YER105C	NUP157	0,892	0,746	1,196	6	8	7	5
YKL156W	RPS27A	0,798	0,669	1,193	46	29	47	28
YFR006W	YFR006W	0,91	0,764	1,191	8	12	8	14
YKL007W	CAP1	0,944	0,793	1,190	12	14	11	14
YER131W	RPS26B	0,983	0,826	1,190	6	4	6	3
YDR089W	YDR089W	0,39	0,328	1,189	3	3	4	3
YHR171W	ATG7	0,831	0,699	1,189	1	1	1	1
YOR164C	GET4	0,923	0,777	1,188	7	10	8	10
YOR270C	VPH1	0,898	0,756	1,188	19	13	19	13
YBR029C	CDS1	0,863	0,727	1,187	2	3	2	3
YOR370C	MRS6	0,838	0,706	1,187	8	5	5	6
YDL117W	CYK3	0,91	0,767	1,186	3	1	4	2
YDR330W	UBX5	0,905	0,763	1,186	2	6	4	6
YHL002W	HSE1	0,687	0,58	1,184	4	5	5	4
YAL001C	TFC3	0,84	0,71	1,183	2	2	2	2
YBR281C	DUG2	0,711	0,601	1,183	6	1	6	3
YEL050C	RML2	0,546	0,462	1,182	1	4	2	4
YOL087C	YOL087C	0,839	0,71	1,182	2	5	3	4
YJR088C	EMC2	0,937	0,793	1,182	7	4	5	3
YAL032C	PRP45	0,827	0,7	1,181	5	6	5	7
YOL045W	PSK2	0,65	0,551	1,180	3	2	3	2
YDL015C	TSC13	0,913	0,774	1,180	5	3	6	4
YBR202W	MCM7	0,309	0,262	1,179	5	4	6	3
YNL321W	VNX1	0,855	0,725	1,179	5	2	6	4
YOR124C	UBP2	0,934	0,792	1,179	5	3	5	2
YOR126C	IAH1	0,999	0,848	1,178	8	8	7	10
YOL148C	SPT20	0,95	0,807	1,177	3	2	1	2
YNR024W	MPP6	0,771	0,655	1,177	5	2	5	2
YEL011W	GLC3	0,76	0,646	1,176	1	1	1	1
YMR061W	RNA14	0,923	0,785	1,176	7	6	9	6
YKR046C	PET10	0,823	0,701	1,174	33	23	31	26
YFL004W	VTC2	0,938	0,799	1,174	19	16	19	15
YER111C	SWI4	0,732	0,624	1,173	8	3	6	3
YGR274C	TAF1	0,888	0,757	1,173	3	6	1	7
YBR081C	SPT7	0,922	0,786	1,173	3	7	3	5
YIR008C	PRI1	0,955	0,816	1,170	10	11	7	10

YNL307C	MCK1	0,949	0,811	1,170	19	24	18	24
YJR099W	YUH1	0,594	0,508	1,169	1	5	1	4
YOR251C	TUM1	0,919	0,786	1,169	10	16	13	15
YOL006C	TOP1	0,865	0,74	1,169	8	10	9	9
YGR215W	RSM27	0,803	0,688	1,167	4	6	4	5
YPL154C	PEP4*	0,126	0,108	1,167	2	4	2	4
YLR439W	MRPL4	0,992	0,851	1,166	4	4	3	4
YLR167W	RPS31	0,811	0,696	1,165	47	35	45	35
YMR250W	GAD1	0,969	0,832	1,165	6	8	3	8
YJR103W	URA8	0,941	0,809	1,163	3	5	3	5
YLL010C	PSR1	0,607	0,522	1,163	3	1	2	1
YER169W	RPH1	0,796	0,685	1,162	3	5	6	5
YPL125W	KAP120	0,864	0,744	1,161	6	2	6	4
YMR196W	YMR196W	0,7	0,603	1,161	3	2	3	2
YPR133C	SPN1	0,96	0,827	1,161	20	25	21	24
YOL009C	MDM12	0,297	0,256	1,160	1	1	1	1
YDL110C	TMA17	0,682	0,588	1,160	8	12	9	11
YMR237W	BCH1	0,871	0,752	1,158	9	5	9	5
YDR031W	MIC14	0,952	0,822	1,158	2	4	3	4
YIL155C	GUT2	0,814	0,703	1,158	12	20	15	22
YER095W	RAD51	0,785	0,678	1,158	5	3	5	3
YLL013C	PUF3	0,964	0,833	1,157	10	14	10	13
YGL210W	YPT32	0,839	0,726	1,156	4	3	5	4
YBR192W	RIM2	0,895	0,775	1,155	6	3	4	3
YOR210W	RPB10	0,978	0,848	1,153	11	14	12	12
YEL007W	YEL007W	0,718	0,623	1,152	3	6	4	5
YKL215C	OXP1	0,932	0,809	1,152	4	5	4	3
YOL008W	COQ10	0,821	0,713	1,151	1	1	1	1
YGR125W	YGR125W	0,905	0,786	1,151	4	1	3	1
YKR003W	OSH6	0,961	0,835	1,151	13	8	11	7
YPR188C	MLC2	0,527	0,458	1,151	6	12	4	11
YGR245C	SDA1	0,785	0,683	1,149	14	13	14	13
YDR141C	DOP1	0,958	0,834	1,149	6	3	5	4
YOL111C	MDY2	0,992	0,864	1,148	11	16	9	16
YOR360C	PDE2	0,863	0,752	1,148	1	1	1	1
YNL284C	MRPL10	0,728	0,635	1,146	22	23	19	21
YLR133W	CKI1	0,892	0,779	1,145	8	8	8	7
YPR133W-A	TOM5	0,9	0,786	1,145	4	3	4	3
YHR137W	ARO9	0,94	0,821	1,145	13	16	13	17

YER047C	SAP1	0,902	0,788	1,145	11	7	8	8
YFL041W	FET5	0,728	0,636	1,145	8	10	7	10
YNR017W	TIM23	0,949	0,83	1,143	9	12	8	10
YNL330C	RPD3	0,702	0,614	1,143	7	12	8	10
YDR192C	NUP42	0,798	0,698	1,143	2	4	2	4
YJL076W	NET1	0,847	0,741	1,143	36	34	34	31
YLR373C	VID22	0,882	0,772	1,142	3	3	4	3
YKR037C	SPC34	0,754	0,66	1,142	2	4	2	4
YDR407C	TRS120	0,827	0,724	1,142	1	2	1	2
YDR497C	ITR1	0,444	0,389	1,141	11	7	11	5
YKL074C	MUD2	0,854	0,749	1,140	4	4	3	3
YAL025C	MAK16	0,816	0,716	1,140	2	5	2	5
YPL086C	ELP3	0,906	0,795	1,140	14	12	13	16
YNL323W	LEM3	0,874	0,767	1,140	3	3	2	3
YER072W	VTC1	0,829	0,728	1,139	2	3	2	2
YOL034W	SMC5	0,905	0,796	1,137	4	7	5	4
YJR042W	NUP85	0,892	0,785	1,136	3	2	4	2
YBL038W	MRPL16	0,612	0,539	1,135	2	1	2	1
YMR130W	YMR130W	0,984	0,867	1,135	5	3	3	4
YPL195W	APL5	0,751	0,662	1,134	4	5	5	4
YDR481C	PHO8	0,937	0,826	1,134	3	3	3	4
YGR266W	YGR266W	0,846	0,746	1,134	5	6	4	3
YNL219C	ALG9	0,678	0,598	1,134	4	3	3	3
YLR129W	DIP2	0,873	0,77	1,134	12	7	13	6
YNL227C	JJJ1	0,933	0,823	1,134	4	2	4	3
YGL228W	SHE10	0,927	0,818	1,133	8	8	8	11
YJL087C	TRL1	0,976	0,862	1,132	3	5	4	6
YGL019W	CKB1	0,905	0,8	1,131	3	6	5	6
YBR233W-A	DAD3	0,923	0,817	1,130	2	2	2	2
YKL103C	LAP4	0,995	0,881	1,129	7	19	8	18
YML025C	YML6	0,947	0,839	1,129	11	13	9	15
YPL184C	MRN1	0,782	0,693	1,128	6	7	5	8
YGL221C	NIF3	0,972	0,862	1,128	11	13	8	11
YGL115W	SNF4	0,993	0,881	1,127	7	8	4	9
YOR197W	MCA1	0,994	0,882	1,127	14	18	12	18
YIL106W	MOB1	0,888	0,788	1,127	6	8	6	8
YHR190W	ERG9	0,962	0,854	1,126	13	10	12	8
YBR279W	PAF1	0,981	0,871	1,126	12	25	14	25
YLR410W	VIP1	0,904	0,803	1,126	17	23	18	25

YOR275C	RIM20	0,761	0,676	1,126	1	1	1	1
YGL151W	NUT1	0,896	0,796	1,126	8	4	9	5
YIL103W	DPH1	0,744	0,661	1,126	7	4	8	7
YBR059C	AKL1	0,996	0,885	1,125	10	10	11	9
YPR019W	MCM4	0,942	0,838	1,124	7	7	7	4
YER087W	AIM10	0,97	0,863	1,124	2	4	3	4
YMR197C	VTI1	0,781	0,695	1,124	2	2	2	2
YBL032W	HEK2	0,857	0,763	1,123	9	8	9	10
YKR068C	BET3	0,767	0,683	1,123	4	3	2	2
YCR051W	YCR051W	0,877	0,781	1,123	7	6	7	6
YNL312W	RFA2	0,8	0,713	1,122	7	13	7	12
YJL061W	NUP82	0,922	0,822	1,122	8	7	5	6
YOL053W	AIM39	0,897	0,8	1,121	3	2	3	2
YMR105C	PGM2	0,835	0,745	1,121	15	13	16	14
YNR026C	SEC12	0,875	0,781	1,120	5	4	4	2
YKL154W	SRP102	0,89	0,795	1,119	4	5	4	2
YMR165C	PAH1	0,817	0,73	1,119	2	1	3	1
YDR493W	MZM1	0,876	0,783	1,119	3	6	3	5
YJL174W	KRE9	0,859	0,768	1,118	4	4	4	3
YOR101W	RAS1	0,963	0,861	1,118	1	1	2	1
YOR340C	RPA43	0,921	0,824	1,118	11	15	10	14
YDR211W	GCD6	0,922	0,825	1,118	8	11	9	9
YBR086C	IST2	0,879	0,787	1,117	24	9	22	11
YBL033C	RIB1	0,786	0,704	1,116	4	9	2	7
YDR328C	SKP1	0,915	0,82	1,116	10	16	13	16
YCL057C-A	YCL057C-A	0,93	0,834	1,115	3	1	2	1
YNL004W	HRB1	0,951	0,853	1,115	4	10	5	11
YGL141W	HUL5	0,713	0,64	1,114	2	3	5	1
YPL030W	TRM44	0,894	0,803	1,113	8	13	7	11
YMR230W	RPS10B	0,887	0,797	1,113	5	2	4	2
YGR163W	GTR2	0,912	0,82	1,112	2	2	2	2
YBR160W	CDC28	0,919	0,827	1,111	13	19	20	19
YLR268W	SEC22	0,812	0,731	1,111	5	4	4	3
YIL117C	PRM5	0,355	0,32	1,109	3	3	3	3
YOR327C	SNC2	0,764	0,689	1,109	6	7	7	7
YBL051C	PIN4	0,877	0,791	1,109	4	8	3	9
YLR438C-A	LSM3	0,961	0,867	1,108	3	4	1	4
YLR181C	VTA1	0,983	0,887	1,108	5	5	4	4
YLR347C	KAP95	0,8	0,722	1,108	15	11	17	11

YDR436W	PPZ2	0,946	0,854	1,108	2	1	2	1
YLR178C	TFS1	0,998	0,901	1,108	11	14	9	13
YAL021C	CCR4	0,929	0,839	1,107	9	14	10	15
YOR290C	SNF2	0,961	0,868	1,107	5	6	4	5
YKR002W	PAP1	0,87	0,786	1,107	3	4	3	4
YDR519W	FPR2	0,86	0,777	1,107	7	11	6	11
YAL060W	BDH1	0,985	0,89	1,107	15	22	12	20
YGL174W	BUD13	0,841	0,76	1,107	2	6	2	6
YGR100W	MDR1	0,728	0,658	1,106	3	2	3	2
YMR202W	ERG2	0,605	0,547	1,106	2	2	2	2
YHR001W-A	QCR10	0,544	0,492	1,106	2	4	4	5
YCR084C	TUP1	0,953	0,862	1,106	21	26	23	27
YML108W	YML108W	0,988	0,894	1,105	2	4	2	4
YHR106W	TRR2	0,873	0,79	1,105	2	4	2	3
YOR155C	ISN1	0,906	0,82	1,105	2	9	2	9
YNR006W	VPS27	0,799	0,724	1,104	7	12	7	10
YML032C	RAD52	0,74	0,671	1,103	7	6	6	5
YGR090W	UTP22	0,859	0,779	1,103	28	18	26	19
YJR059W	PTK2	0,776	0,704	1,102	7	10	8	8
YDR354W	TRP4	0,921	0,836	1,102	6	9	4	10
YDL165W	CDC36	0,995	0,904	1,101	2	5	3	5
YGR191W	HIP1	0,747	0,679	1,100	2	2	3	2
YLL032C	YLL032C	0,892	0,811	1,100	4	1	3	1
YMR047C	NUP116	0,917	0,834	1,100	13	19	16	19
YBL090W	MRP21	0,999	0,909	1,099	12	9	9	13
YOR157C	PUP1	0,905	0,824	1,098	12	8	8	10
YOL142W	RRP40	0,936	0,853	1,097	4	3	5	3
YKR081C	RPF2	0,959	0,874	1,097	21	13	16	13
YNL180C	RHO5	0,886	0,808	1,097	8	7	7	6
YPR051W	MAK3	0,625	0,57	1,096	4	6	5	5
YFL023W	BUD27	0,968	0,883	1,096	13	19	12	21
YDR261C-D	YDR261C-D	0,24	0,219	1,096	1	2	2	2
YGR005C	TFG2	0,917	0,837	1,096	3	6	3	6
YER004W	FMP52	1	0,913	1,095	10	17	12	14
YDR363W-A	SEM1	0,851	0,777	1,095	6	4	6	4
YDR169C	STB3	0,721	0,659	1,094	8	8	6	9
YPL117C	IDI1	0,943	0,862	1,094	13	24	15	22
YMR129W	POM152	0,84	0,768	1,094	14	15	12	13
YHL031C	GOS1	0,902	0,825	1,093	3	4	3	4

YMR266W	RSN1	0,705	0,645	1,093	7	4	8	3
YDR311W	TFB1	0,941	0,861	1,093	1	9	2	9
YBR003W	COQ1	0,944	0,864	1,093	8	20	12	20
YKL067W	YNK1	0,956	0,875	1,093	16	14	16	14
YGL087C	MMS2	0,945	0,865	1,092	4	8	6	6
YHR198C	AIM18	0,975	0,893	1,092	7	7	7	7
YMR302C	YME2	0,943	0,864	1,091	17	15	13	11
YDR084C	TVP23	0,876	0,803	1,091	1	1	2	1
YPL001W	HAT1	0,975	0,894	1,091	7	8	8	10
YPL228W	CET1	0,89	0,818	1,088	14	23	15	22
YDR346C	SVF1	0,943	0,867	1,088	20	23	13	26
YMR109W	MYO5	0,934	0,859	1,087	18	21	16	18
YLR260W	LCB5	0,99	0,911	1,087	2	1	1	1
YLR396C	VPS33	0,815	0,75	1,087	3	2	4	2
YER022W	SRB4	0,96	0,884	1,086	5	6	6	6
YGR048W	UFD1	0,739	0,681	1,085	5	5	5	5
YKR026C	GCN3	0,957	0,882	1,085	9	11	9	10
YOR127W	RGA1	0,741	0,683	1,085	4	6	3	6
YMR124W	YMR124W	0,856	0,789	1,085	9	10	12	12
YJL153C	INO1	0,899	0,829	1,084	34	29	32	27
YHR046C	INM1	0,928	0,856	1,084	8	8	7	8
YJL098W	SAP185	0,759	0,701	1,083	5	6	4	7
YGR049W	SCM4	0,826	0,763	1,083	2	2	2	3
YDR348C	YDR348C	0,842	0,778	1,082	8	13	8	13
YGR116W	SPT6	0,909	0,84	1,082	26	34	31	36
YBL028C	YBL028C	0,489	0,452	1,082	9	8	7	6
YGL167C	PMR1	0,836	0,773	1,082	6	9	7	9
YMR192W	GYL1	0,834	0,772	1,080	1	4	1	4
YLR185W	RPL37A	0,929	0,86	1,080	44	28	43	35
YDL064W	UBC9	0,952	0,882	1,079	4	9	5	8
YHR143W-A	RPC10	0,885	0,82	1,079	15	7	14	7
YCR004C	YCP4	0,845	0,783	1,079	25	14	25	11
YNL327W	EGT2	0,355	0,329	1,079	2	3	1	3
YPR052C	NHP6A	0,865	0,802	1,079	15	8	14	8
YHR111W	UBA4	0,838	0,777	1,079	3	3	2	5
YDR214W	AHA1	0,968	0,898	1,078	20	31	17	28
YBL047C	EDE1	0,933	0,866	1,077	62	70	52	73
YNL088W	TOP2	0,783	0,727	1,077	9	8	7	7
YPL235W	RVB2	0,926	0,86	1,077	30	29	24	30

YDL144C	YDL144C	0,814	0,756	1,077	1	3	1	3
YOR217W	RFC1	0,829	0,77	1,077	8	7	9	8
YLR132C	YLR132C	0,873	0,811	1,076	1	1	1	1
YHR021C	RPS27B	0,848	0,788	1,076	2	4	2	3
YML009C	MRPL39	0,472	0,439	1,075	6	4	6	4
YOR065W	CYT1	0,944	0,878	1,075	2	5	2	5
YEL060C	PRB1	0,873	0,812	1,075	24	25	29	25
YPL037C	EGD1	0,904	0,841	1,075	54	43	51	38
YDL053C	PBP4	0,848	0,789	1,075	13	13	14	14
YMR220W	ERG8	0,951	0,885	1,075	15	19	15	19
YPL013C	MRPS16	0,982	0,914	1,074	7	5	7	5
YBR103W	SIF2	0,889	0,828	1,074	2	6	4	5
YNR023W	SNF12	0,936	0,872	1,073	3	5	2	4
YPL094C	SEC62	0,937	0,873	1,073	9	8	8	8
YDR248C	YDR248C	0,974	0,908	1,073	3	6	4	6
YPL128C	TBF1	0,949	0,885	1,072	5	2	5	2
YMR058W	FET3	0,729	0,68	1,072	7	9	7	8
YJR118C	ILM1	0,804	0,75	1,072	1	2	1	2
YGL091C	NBP35	0,836	0,78	1,072	1	2	1	2
YGR229C	SMI1	0,837	0,781	1,072	13	20	14	22
YKR060W	UTP30	0,914	0,853	1,072	8	8	6	10
YDR068W	DOS2	0,886	0,827	1,071	3	11	1	12
YBR085C-A	YBR085C-A	0,497	0,464	1,071	2	3	3	4
YDR381C-A	YDR381C-A	0,756	0,706	1,071	1	3	2	1
YLR351C	NIT3	0,938	0,876	1,071	7	13	5	12
YOR254C	SEC63	0,915	0,855	1,070	25	18	20	22
YMR178W	YMR178W	0,905	0,846	1,070	7	12	8	11
YMR228W	MTF1	0,798	0,746	1,070	4	6	4	7
YEL047C	YEL047C	0,8	0,748	1,070	23	29	22	28
YML125C	PGA3	0,988	0,924	1,069	4	2	4	3
YOR153W	PDR5	0,79	0,739	1,069	26	27	25	24
YGL137W	SEC27	0,883	0,826	1,069	44	44	44	48
YDL205C	HEM3	0,976	0,913	1,069	9	16	11	18
YBR016W	YBR016W	0,855	0,8	1,069	7	5	7	4
YBL075C	SSA3	0,902	0,844	1,069	1	1	1	1
YLR257W	YLR257W	0,731	0,684	1,069	9	14	10	14
YOR288C	MPD1	0,949	0,888	1,069	5	7	4	9
YGL041W-A	YGL041W-A	0,711	0,666	1,068	5	5	4	5
YDR168W	CDC37	0,901	0,844	1,068	28	41	35	43

YHR099W	TRA1	0,822	0,77	1,068	11	5	9	5
YOL102C	TPT1	0,922	0,864	1,067	1	2	2	2
YFR024C-A	LSB3	0,845	0,792	1,067	10	14	10	12
YJL123C	MTC1	0,975	0,914	1,067	20	22	23	22
YJL145W	SFH5	0,975	0,914	1,067	10	9	9	10
YOR280C	FSH3	0,897	0,841	1,067	3	3	2	3
YLR342W	FKS1	0,773	0,725	1,066	20	20	22	17
YOR362C	PRE10	0,953	0,894	1,066	19	18	18	19
YGR267C	FOL2	0,94	0,882	1,066	9	14	10	14
YNL297C	MON2	0,876	0,822	1,066	5	1	5	3
YDR289C	RTT103	0,879	0,825	1,065	8	16	9	18
YML069W	POB3	0,962	0,903	1,065	9	15	10	15
YDR033W	MRH1	0,855	0,803	1,065	6	5	5	5
YKR084C	HBS1	0,939	0,882	1,065	6	16	6	13
YMR183C	SSO2	0,928	0,872	1,064	5	10	7	9
YER080W	AIM9	0,915	0,86	1,064	30	36	29	38
YBR151W	APD1	0,958	0,901	1,063	9	6	10	7
YMR301C	ATM1	0,859	0,808	1,063	3	1	2	1
YEL017W	GTT3	0,963	0,906	1,063	2	1	2	1
YNL264C	PDR17	0,886	0,834	1,062	5	4	6	4
YFR011C	AIM13	0,756	0,712	1,062	24	24	24	23
YPL050C	MNN9	0,985	0,928	1,061	5	7	8	6
YNL287W	SEC21	0,883	0,832	1,061	32	33	38	37
YLR077W	FMP25	0,711	0,67	1,061	2	1	3	2
YMR261C	TPS3	0,972	0,916	1,061	21	14	18	15
YPR028W	YOP1	0,854	0,805	1,061	10	9	11	10
YPR148C	YPR148C	0,978	0,922	1,061	21	21	20	21
YDL189W	RBS1	0,77	0,726	1,061	3	5	4	4
YMR298W	LIP1	0,861	0,812	1,060	4	4	3	3
YPL273W	SAM4	0,861	0,812	1,060	20	18	13	20
YNL290W	RFC3	0,865	0,816	1,060	5	17	7	17
YML001W	YPT7	0,8	0,755	1,060	7	6	8	6
YDR155C	CPR1	0,947	0,894	1,059	66	54	65	52
YGR001C	YGR001C	0,834	0,788	1,058	7	13	5	13
YDR032C	PST2	0,93	0,879	1,058	23	20	24	19
YPL085W	SEC16	0,899	0,85	1,058	31	34	27	35
YOR112W	CEX1	0,844	0,798	1,058	13	11	13	12
YGL200C	EMP24	0,865	0,818	1,057	4	4	4	5
YDL052C	SLC1	0,928	0,878	1,057	11	6	10	10

YMR075W	RCO1	0,951	0,9	1,057	7	14	5	8
YBR009C	HHF1	0,934	0,884	1,057	19	16	14	16
YOR253W	NAT5	0,863	0,817	1,056	10	14	11	18
YBR142W	MAK5	0,883	0,836	1,056	23	18	21	19
YLR370C	ARC18	0,849	0,804	1,056	9	12	9	11
YDR358W	GGA1	0,837	0,793	1,055	7	6	7	7
YMR315W	YMR315W	0,992	0,94	1,055	29	30	30	31
YPL243W	SRP68	0,86	0,815	1,055	26	18	27	17
YOR119C	RIO1	0,69	0,654	1,055	2	4	1	6
YPL266W	DIM1	0,892	0,846	1,054	8	10	6	10
YLR100W	ERG27	0,897	0,851	1,054	6	9	5	7
YNR029C	YNR029C	0,997	0,946	1,054	4	8	6	9
YDR300C	PRO1	0,943	0,895	1,054	12	17	13	15
YOR089C	VPS21	0,689	0,654	1,054	6	5	6	4
YCL014W	BUD3	0,868	0,824	1,053	12	10	10	10
YFR037C	RSC8	0,891	0,846	1,053	22	31	22	34
YJL171C	YJL171C	0,955	0,907	1,053	6	4	5	4
YJL112W	MDV1	0,784	0,745	1,052	9	7	8	9
YKR070W	YKR070W	0,929	0,883	1,052	3	2	2	2
YML057W	CMP2	0,912	0,867	1,052	18	16	17	15
YNL277W	MET2	0,49	0,466	1,052	9	13	10	12
YBR126C	TPS1	0,942	0,896	1,051	19	17	18	18
YDR001C	NTH1	0,786	0,748	1,051	10	12	8	13
YER177W	BMH1	0,911	0,867	1,051	50	51	49	51
YGL119W	COQ8	0,896	0,853	1,050	4	5	3	4
YDR518W	EUG1	0,86	0,819	1,050	10	13	8	12
YMR024W	MRPL3	0,987	0,94	1,050	16	13	17	16
YLR186W	EMG1	0,93	0,886	1,050	14	22	15	20
YPL232W	SSO1	0,851	0,811	1,049	15	15	14	15
YBR294W	SUL1	0,491	0,468	1,049	12	9	11	7
YLR412W	BER1	0,858	0,818	1,049	2	4	2	5
YHR132W-A	IGO2	0,842	0,803	1,049	2	4	3	4
YLR291C	GCD7	0,933	0,89	1,048	6	6	5	7
YEL034W	HYP2	0,934	0,891	1,048	80	75	79	76
YGR076C	MRPL25	0,807	0,77	1,048	14	11	15	11
YLR430W	SEN1	0,774	0,739	1,047	6	5	6	4
YLR262C-A	TMA7	0,885	0,845	1,047	7	10	8	13
YDR062W	LCB2	0,845	0,807	1,047	5	5	5	5
YDR390C	UBA2	0,897	0,857	1,047	8	14	7	14

YDL056W	MBP1	0,785	0,75	1,047	2	4	3	4
YGR103W	NOP7	0,922	0,881	1,047	29	31	27	29
YDR483W	KRE2	0,676	0,646	1,046	6	11	6	11
YDR177W	UBC1	0,924	0,883	1,046	15	21	13	20
YHR072W	ERG7	0,836	0,799	1,046	4	4	4	4
YCL057W	PRD1	0,904	0,864	1,046	22	45	20	46
YOR195W	SLK19	0,453	0,433	1,046	6	21	5	20
YKL024C	URA6	0,911	0,871	1,046	10	14	12	16
YHR034C	PIH1	0,937	0,896	1,046	7	7	7	11
YLR028C	ADE16	0,825	0,789	1,046	25	26	26	28
YMR086W	YMR086W	0,826	0,79	1,046	22	27	25	23
YLR398C	SKI2	0,877	0,839	1,045	21	14	19	15
YHR088W	RPF1	0,739	0,707	1,045	11	14	13	14
YDL160C	DHH1	0,855	0,818	1,045	24	32	27	33
YGR111W	YGR111W	0,926	0,886	1,045	3	6	4	6
YOR272W	YTM1	0,982	0,94	1,045	25	30	23	27
YMR201C	RAD14	0,889	0,851	1,045	1	2	1	2
YGR130C	YGR130C	0,759	0,727	1,044	59	38	54	38
YKL151C	YKL151C	0,997	0,955	1,044	1	2	2	2
YNL045W	LAP2	0,811	0,777	1,044	9	20	10	19
YAL036C	RBG1	0,859	0,823	1,044	21	18	21	17
YDL088C	ASM4	0,914	0,876	1,043	4	5	5	5
YCR060W	TAH1	0,872	0,836	1,043	1	3	1	3
YKL179C	COY1	0,899	0,862	1,043	12	12	13	10
YKL025C	PAN3	0,802	0,769	1,043	2	4	2	4
YCL028W	RNQ1	0,973	0,933	1,043	3	8	4	9
YNL079C	TPM1	0,929	0,891	1,043	20	25	26	23
YDR361C	BCP1	0,834	0,8	1,043	6	12	9	14
YBR058C	UBP14	0,69	0,662	1,042	10	14	12	10
YJR085C	YJR085C	0,914	0,877	1,042	1	2	2	2
YKL112W	ABF1	0,846	0,812	1,042	6	11	6	12
YGR148C	RPL24B	0,897	0,861	1,042	8	7	7	6
YPR048W	TAH18	0,899	0,863	1,042	7	2	7	5
YOR361C	PRT1	0,907	0,871	1,041	51	68	47	70
YDR430C	CYM1	0,986	0,947	1,041	33	37	29	37
YDL130W-A	STF1	0,885	0,85	1,041	7	3	6	4
YGR271C-A	EFG1	0,887	0,852	1,041	9	17	8	16
YER101C	AST2	0,888	0,853	1,041	5	6	3	6
YNL005C	MRP7	0,89	0,855	1,041	12	13	13	14

YBR269C	FMP21	0,612	0,588	1,041	9	11	11	11
YLL021W	SPA2	0,819	0,787	1,041	30	22	31	21
YNL207W	RIO2	0,798	0,767	1,040	5	4	4	4
YBR112C	CYC8	0,78	0,75	1,040	12	22	13	22
YPR187W	RPO26	0,966	0,929	1,040	9	9	10	10
YDR175C	RSM24	0,969	0,932	1,040	8	11	6	11
YBL041W	PRE7	0,923	0,888	1,039	16	19	13	18
YNL298W	CLA4	0,818	0,787	1,039	4	6	4	6
YPL012W	RRP12	0,845	0,813	1,039	24	14	24	15
YGR017W	YGR017W	0,951	0,915	1,039	4	5	4	4
YGL207W	SPT16	0,846	0,814	1,039	40	37	35	34
YHR066W	SSF1	0,9	0,866	1,039	7	6	6	7
YER042W	MXR1	0,857	0,825	1,039	12	19	15	20
YNL059C	ARP5	0,911	0,877	1,039	11	9	13	10
YER012W	PRE1	0,919	0,885	1,038	6	6	6	8
YIL137C	TMA108	0,84	0,809	1,038	9	16	8	15
YKL117W	SBA1	0,961	0,926	1,038	15	10	13	10
YOR211C	MGM1	0,961	0,926	1,038	11	17	14	18
YDL171C	GLT1	0,831	0,801	1,037	58	69	63	66
YCL035C	GRX1	0,888	0,856	1,037	8	9	9	10
YOR132W	VPS17	0,866	0,835	1,037	6	9	4	10
YPL245W	YPL245W	0,783	0,755	1,037	2	3	1	3
YGL105W	ARC1	0,926	0,893	1,037	50	52	53	57
YLR212C	TUB4	0,819	0,79	1,037	1	2	1	2
YNL243W	SLA2	0,848	0,818	1,037	31	19	28	18
YBL069W	AST1	0,851	0,821	1,037	5	7	5	6
YBL026W	LSM2	0,884	0,853	1,036	3	7	3	7
YER019C-A	SBH2	0,856	0,826	1,036	6	6	6	6
YML030W	AIM31	0,799	0,771	1,036	2	2	2	2
YHR027C	RPN1	0,887	0,856	1,036	34	34	30	34
YDR502C	SAM2	0,899	0,868	1,036	76	48	72	47
YNL016W	PUB1	0,913	0,882	1,035	12	11	8	11
YCR090C	YCR090C	0,944	0,912	1,035	2	7	3	7
YGR082W	TOM20	0,829	0,801	1,035	6	6	7	6
YCL043C	PDI1	0,861	0,832	1,035	59	64	58	62
YMR092C	AIP1	0,891	0,861	1,035	13	19	15	20
YHR081W	LRP1	0,892	0,862	1,035	14	11	13	11
YBL003C	HTA2	0,895	0,865	1,035	24	18	26	15
YBR205W	KTR3	0,896	0,866	1,035	2	4	1	4

YDL019C	OSH2	0,812	0,785	1,034	7	13	10	14
YKL128C	PMU1	0,908	0,878	1,034	6	10	7	11
YBL061C	SKT5	0,639	0,618	1,034	7	6	7	8
YMR039C	SUB1	0,917	0,887	1,034	5	14	6	14
YBR162W-A	YSY6	0,766	0,741	1,034	2	2	2	2
YDL058W	USO1	0,92	0,89	1,034	14	15	17	15
YGL092W	NUP145	0,862	0,834	1,034	21	20	20	21
YER120W	SCS2	0,895	0,866	1,033	24	23	26	22
YKL113C	RAD27	0,93	0,9	1,033	11	10	8	9
YIL002W-A	YIL002W-A	0,714	0,691	1,033	6	8	7	8
YIR037W	HYR1	0,933	0,903	1,033	16	21	13	22
YLR147C	SMD3	0,999	0,967	1,033	1	3	1	3
YPR144C	NOC4	0,931	0,902	1,032	10	8	11	10
YKR035W-A	DID2	0,838	0,812	1,032	13	5	11	5
YCL050C	APA1	0,903	0,875	1,032	34	37	31	35
YER154W	OXA1	0,743	0,72	1,032	6	6	5	7
YHR073W	OSH3	0,841	0,815	1,032	13	13	11	12
YPL146C	NOP53	0,907	0,879	1,032	22	16	20	15
YDR074W	TPS2	0,847	0,821	1,032	27	37	32	35
YLR321C	SFH1	0,915	0,887	1,032	2	4	3	4
YDR515W	SLF1	0,854	0,828	1,031	11	13	10	12
YML028W	TSA1	0,92	0,892	1,031	25	22	24	24
YHR084W	STE12	0,628	0,609	1,031	3	5	4	5
YGL037C	PNC1	0,864	0,838	1,031	11	16	10	14
YML050W	AIM32	0,801	0,777	1,031	3	1	3	1
YPL180W	TCO89	0,869	0,843	1,031	4	6	2	6
YNL209W	SSB2	0,84	0,815	1,031	30	22	31	21
YDR320C	SWA2	0,813	0,789	1,030	8	15	9	13
YDR054C	CDC34	0,578	0,561	1,030	1	1	1	1
YDL022W	GPD1	0,864	0,839	1,030	21	23	23	21
YIR004W	DJP1	0,868	0,843	1,030	24	29	20	29
YNL037C	IDH1	0,906	0,88	1,030	38	37	36	38
YGL014W	PUF4	0,84	0,816	1,029	12	18	12	17
YCL031C	RRP7	0,876	0,851	1,029	10	11	7	11
YPR035W	GLN1	0,777	0,755	1,029	23	26	21	23
YNL189W	SRP1	0,885	0,86	1,029	25	20	26	15
YDR490C	PKH1	0,714	0,694	1,029	2	3	2	3
YNL169C	PSD1	0,715	0,695	1,029	7	3	7	4
YJL055W	YJL055W	0,935	0,909	1,029	14	17	12	17

YPL063W	TIM50	0,87	0,846	1,028	22	24	18	23
YML078W	CPR3	0,943	0,917	1,028	30	22	29	28
YML072C	TCB3	0,836	0,813	1,028	45	34	49	35
YLR387C	REH1	0,807	0,785	1,028	12	12	12	12
YNL067W	RPL9B	0,92	0,895	1,028	2	2	2	2
YIR001C	SGN1	0,892	0,868	1,028	8	10	8	10
YCL019W	YCL019W	0,526	0,512	1,027	8	12	8	13
YKL210W	UBA1	0,867	0,844	1,027	53	67	52	69
YGR106C	VOA1	0,381	0,371	1,027	2	5	3	5
YGR132C	PHB1	0,926	0,902	1,027	19	22	19	23
YBR096W	YBR096W	0,854	0,832	1,026	2	2	2	2
YML102W	CAC2	0,896	0,873	1,026	2	5	2	3
YBR146W	MRPS9	0,902	0,879	1,026	16	18	11	16
YLL034C	RIX7	0,944	0,92	1,026	14	16	13	16
YMR072W	ABF2	0,945	0,921	1,026	21	21	21	21
YHR200W	RPN10	0,87	0,848	1,026	8	12	9	12
YML056C	IMD4	0,873	0,851	1,026	19	18	16	15
YLR206W	ENT2	0,841	0,82	1,026	7	10	8	10
YHR098C	SFB3	0,817	0,797	1,025	11	12	12	11
YGL201C	MCM6	0,741	0,723	1,025	8	12	11	14
YEL013W	VAC8	0,827	0,807	1,025	19	12	20	11
YGL107C	RMD9	0,912	0,89	1,025	8	9	11	11
YOR207C	RET1	0,75	0,732	1,025	13	9	14	10
YAL033W	POP5	0,917	0,895	1,025	3	4	1	3
YFR053C	HXK1	0,797	0,778	1,024	37	43	37	41
YMR318C	ADH6	0,897	0,876	1,024	23	39	28	39
YER025W	GCD11	0,857	0,837	1,024	50	56	46	55
YHR001W	OSH7	0,865	0,845	1,024	16	29	14	26
YBL059C-A	CMC2	0,752	0,735	1,023	12	10	12	13
YBL011W	SCT1	0,841	0,822	1,023	3	1	3	1
YOR308C	SNU66	0,851	0,832	1,023	2	3	3	3
YDL040C	NAT1	0,898	0,878	1,023	21	31	20	29
YMR044W	IOC4	0,724	0,708	1,023	1	2	1	2
YKL213C	DOA1	0,819	0,801	1,022	13	14	12	14
YKL201C	MNN4	0,729	0,713	1,022	12	8	10	5
YBR078W	ECM33	0,739	0,723	1,022	28	17	25	17
YIL136W	OM45	0,885	0,866	1,022	22	26	21	26
YDR251W	PAM1	0,801	0,784	1,022	7	11	8	10
YOR151C	RPB2	0,711	0,696	1,022	20	22	19	21

YJL148W	RPA34	0,927	0,908	1,021	11	9	11	11
YLR247C	IRC20	0,586	0,574	1,021	1	1	1	1
YJL167W	ERG20	0,934	0,915	1,021	42	34	48	39
YNL010W	YNL010W	0,892	0,874	1,021	23	35	25	32
YFR001W	LOC1	0,855	0,838	1,020	15	9	15	11
YCR077C	PAT1	0,858	0,841	1,020	17	14	15	13
YDR079C-A	TFB5	0,625	0,613	1,020	1	4	2	5
YHR104W	GRE3	0,945	0,927	1,019	15	25	14	27
YPR161C	SGV1	0,738	0,724	1,019	11	8	11	7
YJR143C	PMT4	0,849	0,833	1,019	9	5	7	5
YML010W	SPT5	0,796	0,781	1,019	30	43	36	46
YNL149C	PGA2	0,858	0,842	1,019	7	5	7	7
YMR147W	YMR147W	0,699	0,686	1,019	5	2	4	3
YLR150W	STM1	0,861	0,845	1,019	98	72	91	70
YIL075C	RPN2	0,821	0,806	1,019	27	38	27	36
YMR172W	HOT1	0,779	0,765	1,018	1	2	1	2
YDR296W	MHR1	0,844	0,829	1,018	4	9	4	10
YLR166C	SEC10	0,738	0,725	1,018	3	3	2	3
YBR104W	YMC2	0,922	0,906	1,018	4	2	3	2
YBR199W	KTR4	0,405	0,398	1,018	6	2	7	3
YLR203C	MSS51	0,93	0,914	1,018	10	15	14	15
YDR139C	RUB1	0,939	0,923	1,017	4	4	4	4
YGR097W	ASK10	0,766	0,753	1,017	11	10	12	6
YJR105W	ADO1	0,9	0,885	1,017	77	52	76	50
YKL029C	MAE1	0,785	0,772	1,017	31	35	30	34
YLR182W	SWI6	0,667	0,656	1,017	4	5	4	4
YJL033W	HCA4	0,851	0,837	1,017	23	18	20	18
YJL136C	RPS21B	0,854	0,84	1,017	50	33	47	29
YNL111C	CYB5	0,981	0,965	1,017	6	4	5	3
YBR017C	KAP104	0,8	0,787	1,017	8	7	7	10
YER123W	YCK3	0,925	0,91	1,016	6	2	6	1
YPR033C	HTS1	0,864	0,85	1,016	40	40	39	40
YGL055W	OLE1	0,372	0,366	1,016	20	12	16	12
YJL081C	ARP4	0,942	0,927	1,016	6	11	6	8
YLL040C	VPS13	0,755	0,743	1,016	10	9	11	10
YLR314C	CDC3	0,823	0,81	1,016	25	37	28	37
YGR124W	ASN2	0,889	0,875	1,016	38	36	36	36
YCL016C	DCC1	0,767	0,755	1,016	2	4	3	4
YGL244W	RTF1	0,772	0,76	1,016	12	15	14	15

YDL124W	YDL124W	0,849	0,836	1,016	51	55	48	52
YER136W	GDI1	0,915	0,901	1,016	27	40	26	37
YPL139C	UME1	0,852	0,839	1,015	10	17	7	15
YKR025W	RPC37	0,856	0,843	1,015	14	18	9	16
YAL005C	SSA1	0,863	0,85	1,015	38	37	41	37
YKL181W	PRS1	0,935	0,921	1,015	11	18	13	16
YGR262C	BUD32	0,87	0,857	1,015	4	6	5	6
YNL137C	NAM9	0,897	0,884	1,015	17	16	15	17
YDR101C	ARX1	0,899	0,886	1,015	21	27	22	30
YCR028C-A	RIM1	0,91	0,897	1,014	23	17	22	17
YFR051C	RET2	0,856	0,844	1,014	17	27	19	24
YBR155W	CNS1	1	0,986	1,014	16	26	16	24
YDR432W	NPL3	0,931	0,918	1,014	10	10	13	10
YDR012W	RPL4B	0,937	0,924	1,014	9	5	8	6
YNR022C	MRPL50	0,585	0,577	1,014	4	6	3	6
YDR224C	HTB1	0,879	0,867	1,014	43	31	42	32
YOR215C	AIM41	0,882	0,87	1,014	12	19	10	19
YLR335W	NUP2	0,812	0,801	1,014	46	48	43	48
YOR243C	PUS7	0,889	0,877	1,014	28	40	30	39
YDR091C	RLI1	0,894	0,882	1,014	42	41	37	42
YHR051W	COX6	0,835	0,824	1,013	15	22	17	21
YKL027W	YKL027W	0,837	0,826	1,013	9	11	8	11
YDR129C	SAC6	0,843	0,832	1,013	34	34	36	32
YDR150W	NUM1	0,855	0,844	1,013	30	40	30	38
YLR301W	YLR301W	0,947	0,935	1,013	15	22	11	23
YML127W	RSC9	0,793	0,783	1,013	11	6	10	8
YPR002W	PDH1	0,952	0,94	1,013	3	10	4	9
YGR254W	ENO1	0,878	0,867	1,013	64	32	62	40
YEL031W	SPF1	0,879	0,868	1,013	31	22	31	22
YGR232W	NAS6	0,885	0,874	1,013	3	9	4	9
YOR141C	ARP8	0,815	0,805	1,012	8	9	11	8
YLR104W	LCL2	0,906	0,895	1,012	5	1	5	1
YHR039C-A	VMA10	0,837	0,827	1,012	23	15	26	15
YMR112C	MED11	0,758	0,749	1,012	4	4	3	4
YDR529C	QCR7	0,856	0,846	1,012	26	12	26	10
YPR129W	SCD6	0,794	0,785	1,011	10	17	9	15
YBL007C	SLA1	0,89	0,88	1,011	23	35	25	35
YOL005C	RPB11	0,891	0,881	1,011	5	13	8	12
YDL140C	RPO21	0,714	0,706	1,011	46	45	44	44

YEL020W-A	TIM9	0,994	0,983	1,011	16	13	16	14
YGR244C	LSC2	0,916	0,906	1,011	46	42	43	48
YOL027C	MDM38	0,938	0,928	1,011	24	26	21	24
YIL126W	STH1	0,846	0,837	1,011	15	17	15	15
YKL126W	YPK1	0,851	0,842	1,011	11	12	12	14
YKL145W	RPT1	0,856	0,847	1,011	15	18	12	18
YDR292C	SRP101	0,868	0,859	1,010	18	24	22	24
YBR256C	RIB5	0,97	0,96	1,010	11	14	8	13
YHR043C	DOG2	0,876	0,867	1,010	2	7	3	7
YMR309C	NIP1	0,889	0,88	1,010	42	34	40	33
YNL201C	PSY2	0,803	0,795	1,010	3	4	2	3
YGL064C	MRH4	0,932	0,923	1,010	6	8	6	9
YPR103W	PRE2	0,829	0,821	1,010	5	9	6	9
YJL012C	VTC4	0,726	0,719	1,010	13	9	12	12
YMR193W	MRPL24	0,726	0,719	1,010	17	17	14	15
YDL195W	SEC31	0,837	0,829	1,010	68	71	62	68
YHR197W	RIX1	0,74	0,733	1,010	11	7	11	8
YDR316W	OMS1	0,953	0,944	1,010	4	3	4	3
YML016C	PPZ1	0,848	0,84	1,010	11	7	11	6
YOR230W	WTM1	0,857	0,849	1,009	44	44	43	45
YNL074C	MLF3	0,543	0,538	1,009	3	5	2	5
YDR172W	SUP35	0,873	0,865	1,009	38	46	37	41
YDL136W	RPL35B	0,877	0,869	1,009	90	54	85	49
YCL040W	GLK1	0,881	0,873	1,009	36	34	42	33
YBR169C	SSE2	0,882	0,874	1,009	8	15	7	16
YNL160W	YGP1	0,78	0,773	1,009	17	14	16	15
YOL041C	NOP12	0,792	0,785	1,009	38	34	40	36
YGR173W	RBG2	0,797	0,79	1,009	13	15	12	17
YIL108W	YIL108W	0,919	0,911	1,009	12	6	11	6
YIL010W	DOT5	0,805	0,798	1,009	15	14	13	11
YBL036C	YBL036C	0,932	0,924	1,009	18	21	13	18
YPL118W	MRP51	0,937	0,929	1,009	10	17	10	18
YAL030W	SNC1	0,82	0,813	1,009	3	3	3	5
YLR229C	CDC42	0,835	0,828	1,008	8	9	7	7
YGL056C	SDS23	0,844	0,837	1,008	10	13	12	13
YIL076W	SEC28	0,848	0,841	1,008	14	14	10	14
YBR154C	RPB5	0,852	0,845	1,008	12	14	12	16
YER068W	MOT2	0,744	0,738	1,008	11	8	10	10
YDR447C	RPS17B	0,869	0,862	1,008	96	47	91	49

YNL058C	YNL058C	0,382	0,379	1,008	1	1	2	1
YJR083C	ACF4	0,782	0,776	1,008	6	7	7	8
YFL018C	LPD1	0,934	0,927	1,008	56	54	53	55
YGR235C	YGR235C	0,674	0,669	1,007	5	7	7	7
YDR381W	YRA1	0,834	0,828	1,007	25	23	27	23
YKL035W	UGP1	0,844	0,838	1,007	36	39	36	40
YLR438W	CAR2	0,857	0,851	1,007	52	45	57	46
YKL152C	GPM1	0,859	0,853	1,007	149	115	146	112
YKL142W	MRP8	0,88	0,874	1,007	30	36	29	37
YEL052W	AFG1	0,903	0,897	1,007	8	8	5	7
YHR005C-A	TIM10	0,911	0,905	1,007	24	21	26	22
YJL130C	URA2	0,774	0,769	1,007	151	135	151	144
YPL215W	CBP3	0,975	0,969	1,006	14	6	12	9
YNL015W	PBI2	0,981	0,975	1,006	10	9	10	9
YNL173C	MDG1	0,846	0,841	1,006	16	21	18	21
YBL092W	RPL32	0,888	0,883	1,006	81	61	77	54
YDR156W	RPA14	0,892	0,887	1,006	8	10	10	10
YDR454C	GUK1	0,901	0,896	1,006	44	31	40	32
YMR070W	MOT3	0,744	0,74	1,005	2	2	2	2
YDR303C	RSC3	0,95	0,945	1,005	10	6	7	6
YPR191W	QCR2	0,975	0,97	1,005	67	43	65	42
YDR380W	ARO10	0,395	0,393	1,005	14	14	13	14
YGR186W	TFG1	0,802	0,798	1,005	27	27	21	25
YLR118C	YLR118C	0,812	0,808	1,005	4	9	5	8
YBR245C	ISW1	0,826	0,822	1,005	37	35	35	38
YHL011C	PRS3	0,827	0,823	1,005	38	34	35	28
YMR304W	UBP15	0,839	0,835	1,005	5	7	7	5
YOR322C	LDB19	0,867	0,863	1,005	2	1	2	1
YLR293C	GSP1	0,878	0,874	1,005	36	37	37	37
YHL034C	SBP1	0,89	0,886	1,005	34	27	34	29
YNR053C	NOG2	0,683	0,68	1,004	26	27	25	28
YPL132W	COX11	0,916	0,912	1,004	2	3	3	2
YGR207C	YGR207C	0,94	0,936	1,004	28	29	32	30
YDL125C	HNT1	0,841	0,838	1,004	19	22	17	22
YKL157W	APE2	0,863	0,86	1,003	43	52	39	51
YGL135W	RPL1B	0,875	0,872	1,003	76	62	78	55
YKR001C	VPS1	0,888	0,885	1,003	44	56	37	53
YNL055C	POR1	0,948	0,945	1,003	47	42	41	42
YCL024W	KCC4	0,762	0,76	1,003	4	3	4	4

YAL038W	CDC19	0,857	0,855	1,002	261	220	259	225
YDR099W	BMH2	0,865	0,863	1,002	9	14	10	13
YFL038C	YPT1	0,869	0,867	1,002	11	7	10	6
YKL196C	YKT6	0,885	0,883	1,002	10	8	13	8
YLR131C	ACE2	0,485	0,484	1,002	4	2	2	3
YOR374W	ALD4	0,989	0,987	1,002	56	58	55	61
YGL150C	INO80	0,775	0,774	1,001	10	8	9	8
YLR221C	RSA3	0,785	0,784	1,001	8	7	9	9
YMR226C	YMR226C	0,845	0,844	1,001	37	28	35	31
YGR056W	RSC1	0,863	0,862	1,001	2	2	2	1
YMR142C	RPL13B	0,864	0,863	1,001	91	57	89	60
YHR128W	FUR1	0,865	0,864	1,001	20	23	19	24
YGL206C	CHC1	0,869	0,868	1,001	64	62	65	57
YDR424C	DYN2	0,87	0,869	1,001	4	10	6	8
YLR153C	ACS2	0,873	0,872	1,001	59	58	59	57
YOR117W	RPT5	0,873	0,872	1,001	50	53	48	53
YMR242C	RPL20A	0,875	0,874	1,001	74	58	78	62
YGR187C	HGH1	0,884	0,883	1,001	7	13	7	15
YER074W	RPS24A	0,887	0,886	1,001	89	57	89	54
YOL109W	ZEO1	0,917	0,916	1,001	33	21	32	22
YGL130W	CEG1	0,942	0,941	1,001	4	10	2	10
YDL031W	DBP10	0,915	0,915	1,000	30	42	26	37
YDL147W	RPN5	0,882	0,882	1,000	16	22	19	23
YDR237W	MRPL7	0,955	0,955	1,000	10	11	11	13
YDR408C	ADE8	0,842	0,842	1,000	11	10	11	9
YDR533C	HSP31	0,934	0,934	1,000	20	23	18	23
YER182W	FMP10	0,82	0,82	1,000	4	4	3	4
YGR159C	NSR1	0,804	0,804	1,000	21	21	21	25
YGR261C	APL6	0,839	0,839	1,000	11	7	8	6
YHR074W	QNS1	0,8	0,8	1,000	8	9	9	9
YJL080C	SCP160	0,888	0,888	1,000	154	140	149	131
YJR070C	LIA1	0,852	0,852	1,000	40	35	36	35
YKR014C	YPT52	0,991	0,991	1,000	8	10	6	8
YLR328W	NMA1	0,952	0,952	1,000	7	4	6	6
YNL313C	EMW1	0,934	0,934	1,000	10	13	12	16
YPR105C	COG4	0,927	0,927	1,000	4	2	3	3
YDL174C	DLD1	0,935	0,936	0,999	30	30	28	28
YBR085W	AAC3	0,933	0,934	0,999	1	1	1	1
YER055C	HIS1	0,914	0,915	0,999	27	32	31	31

YBR084C-A	RPL19A	0,877	0,878	0,999	84	41	79	41
YMR074C	YMR074C	0,874	0,875	0,999	9	11	9	8
YOR369C	RPS12	0,865	0,866	0,999	47	32	48	28
YMR029C	FAR8	0,862	0,863	0,999	3	9	4	9
YHR169W	DBP8	0,849	0,85	0,999	5	5	6	5
YNL231C	PDR16	0,827	0,828	0,999	13	21	13	19
YOR332W	VMA4	0,806	0,807	0,999	66	55	63	58
YOR181W	LAS17	0,788	0,789	0,999	7	5	5	5
YBR273C	UBX7	0,668	0,669	0,999	7	10	3	10
YBR225W	YBR225W	0,502	0,503	0,998	1	1	1	1
YKL192C	ACP1	0,955	0,957	0,998	6	4	5	4
YFL016C	MDJ1	0,859	0,861	0,998	14	23	15	20
YMR219W	ESC1	0,81	0,812	0,998	9	10	9	12
YMR110C	HFD1	0,956	0,959	0,997	11	13	10	10
YDR280W	RRP45	0,926	0,929	0,997	3	7	3	7
YGR209C	TRX2	0,893	0,896	0,997	18	13	20	15
YKL206C	ADD66	0,874	0,877	0,997	2	5	3	5
YLR027C	AAT2	0,869	0,872	0,997	56	68	61	65
YPR132W	RPS23B	0,865	0,868	0,997	66	51	64	50
YCR093W	CDC39	0,815	0,818	0,996	18	22	21	22
YPR173C	VPS4	0,792	0,795	0,996	8	16	10	17
YLR163C	MAS1	0,788	0,791	0,996	17	23	20	24
YML092C	PRE8	0,957	0,961	0,996	23	19	23	21
YKL091C	YKL091C	0,903	0,907	0,996	1	3	1	3
YHR121W	LSM12	0,894	0,898	0,996	13	16	10	15
YLR303W	MET17	0,889	0,893	0,996	127	99	131	99
YDR037W	KRS1	0,875	0,879	0,995	79	74	81	79
YER091C	MET6	0,872	0,876	0,995	357	263	363	261
YML126C	ERG13	0,852	0,856	0,995	44	33	40	36
YGL111W	NSA1	0,826	0,83	0,995	16	12	16	12
YNR074C	AIF1	0,934	0,939	0,995	1	6	2	7
YDL046W	NPC2	0,893	0,898	0,994	1	1	2	1
YLR359W	ADE13	0,888	0,893	0,994	32	37	34	39
YGR180C	RNR4	0,881	0,886	0,994	35	46	34	48
YGR204W	ADE3	0,875	0,88	0,994	94	94	94	97
YJL190C	RPS22A	0,871	0,876	0,994	42	44	43	43
YPL010W	RET3	0,853	0,858	0,994	8	6	10	4
YJR046W	TAH11	0,852	0,857	0,994	1	1	1	1
YDL185W	VMA1	0,831	0,836	0,994	127	136	133	135

YIL093C	RSM25	0,993	0,999	0,994	8	15	9	15
YLL026W	HSP104	0,811	0,816	0,994	68	80	64	81
YNL208W	YNL208W	0,783	0,788	0,994	9	5	9	5
YCR072C	RSA4	0,915	0,921	0,993	8	6	9	5
YBL022C	PIM1	0,906	0,912	0,993	39	48	36	46
YLR250W	SSP120	0,743	0,748	0,993	3	10	3	9
YGR034W	RPL26B	0,888	0,894	0,993	82	55	83	49
YJR094W-A	RPL43B	0,888	0,894	0,993	44	30	41	30
YKL006W	RPL14A	0,888	0,894	0,993	70	56	74	56
YER117W	RPL23B	0,879	0,885	0,993	37	34	33	35
YHR203C	RPS4B	0,879	0,885	0,993	96	86	100	85
YGR194C	XKS1	0,875	0,881	0,993	2	5	3	5
YDR050C	TPI1	0,84	0,846	0,993	61	69	60	70
YOR187W	TUF1	0,964	0,971	0,993	42	40	43	35
YOR061W	CKA2	0,826	0,832	0,993	10	17	13	19
YFR044C	DUG1	0,912	0,919	0,992	39	43	41	40
YJL189W	RPL39	0,884	0,891	0,992	15	12	14	12
YDR507C	GIN4	0,505	0,509	0,992	23	13	26	16
YDL075W	RPL31A	0,881	0,888	0,992	60	35	58	27
YGL245W	GUS1	0,875	0,882	0,992	69	74	73	73
YPR036W	VMA13	0,859	0,866	0,992	37	32	33	33
YER110C	KAP123	0,844	0,851	0,992	52	56	55	55
YHR087W	RTC3	0,958	0,966	0,992	8	7	7	7
YNL123W	NMA111	0,832	0,839	0,992	19	22	17	23
YDR221W	GTB1	0,815	0,822	0,991	9	5	7	7
YGL171W	ROK1	0,808	0,815	0,991	11	14	12	15
YOR175C	ALE1	0,808	0,815	0,991	3	2	3	1
YNR051C	BRE5	0,892	0,9	0,991	19	28	15	30
YNL131W	TOM22	0,887	0,895	0,991	15	9	17	10
YFL022C	FRS2	0,87	0,878	0,991	56	48	57	50
YGL001C	ERG26	0,868	0,876	0,991	16	18	13	17
YER133W	GLC7	0,831	0,839	0,990	7	16	6	17
YDR044W	HEM13	0,83	0,838	0,990	6	17	8	18
YGR231C	PHB2	0,921	0,93	0,990	26	18	26	21
YLR401C	DUS3	0,806	0,814	0,990	11	19	13	18
YMR140W	SIP5	0,894	0,903	0,990	2	2	2	3
YNR021W	YNR021W	0,888	0,897	0,990	15	12	13	9
YBR041W	FAT1	0,885	0,894	0,990	5	5	4	4
YNR035C	ARC35	0,885	0,894	0,990	17	14	14	16

YCL054W	SPB1	0,784	0,792	0,990	32	27	28	28
YML063W	RPS1B	0,877	0,886	0,990	95	77	93	67
YKL216W	URA1	0,779	0,787	0,990	77	77	73	72
YHR127W	YHR127W	0,867	0,876	0,990	5	6	5	7
YDR188W	CCT6	0,864	0,873	0,990	36	39	35	37
YMR229C	RRP5	0,863	0,872	0,990	99	99	94	103
YOR204W	DED1	0,859	0,868	0,990	49	37	48	32
YBR221C	PDB1	0,849	0,858	0,990	29	28	31	29
YLR432W	IMD3	0,849	0,858	0,990	63	54	59	54
YPL028W	ERG10	0,902	0,912	0,989	51	45	48	39
YDR135C	YCF1	0,804	0,813	0,989	13	7	11	6
YDR320C-A	DAD4	0,801	0,81	0,989	2	2	2	2
YDL213C	NOP6	0,889	0,899	0,989	5	10	7	11
YGL143C	MRF1	0,886	0,896	0,989	1	3	1	3
YPL131W	RPL5	0,883	0,893	0,989	67	40	65	41
YGL189C	RPS26A	0,882	0,892	0,989	58	53	57	51
YDR002W	YRB1	0,868	0,878	0,989	38	32	36	33
YLR180W	SAM1	0,862	0,872	0,989	142	111	139	118
YKL054C	DEF1	0,852	0,862	0,988	40	31	41	31
YDL100C	GET3	0,845	0,855	0,988	22	29	22	30
YIL142W	CCT2	0,84	0,85	0,988	60	43	55	43
YCL010C	SGF29	0,832	0,842	0,988	5	3	3	4
YLR295C	ATP14	0,742	0,751	0,988	6	13	6	14
YPL263C	KEL3	0,892	0,903	0,988	4	2	3	4
YDL181W	INH1	0,877	0,888	0,988	8	4	8	6
YOL049W	GSH2	0,868	0,879	0,987	12	20	10	20
YGR085C	RPL11B	0,864	0,875	0,987	67	40	70	45
YBR118W	TEF2	0,857	0,868	0,987	356	276	343	268
YPL231W	FAS2	0,853	0,864	0,987	241	196	237	185
YDR353W	TRR1	0,852	0,863	0,987	60	52	61	45
YGR019W	UGA1	0,846	0,857	0,987	20	24	21	20
YLR043C	TRX1	0,922	0,934	0,987	35	33	29	30
YBR037C	SCO1	0,918	0,93	0,987	6	5	6	5
YOR074C	CDC21	0,823	0,834	0,987	4	12	8	13
YGL031C	RPL24A	0,894	0,906	0,987	65	56	61	54
YGR027C	RPS25A	0,879	0,891	0,987	37	20	35	24
YJL034W	KAR2	0,876	0,888	0,986	72	75	82	75
YNL121C	TOM70	0,939	0,952	0,986	59	59	58	58
YOR241W	MET7	0,862	0,874	0,986	9	16	8	18

YHR042W	NCP1	0,85	0,862	0,986	28	30	30	30
YMR009W	ADI1	0,637	0,646	0,986	5	8	5	8
YPL049C	DIG1	0,773	0,784	0,986	8	11	10	10
YEL032W	MCM3	0,627	0,636	0,986	9	23	8	23
YLR096W	KIN2	0,966	0,98	0,986	4	2	4	4
YBR048W	RPS11B	0,89	0,903	0,986	66	42	66	55
YMR194W	RPL36A	0,881	0,894	0,985	65	46	63	41
YIL033C	BCY1	0,88	0,893	0,985	30	29	29	29
YPL061W	ALD6	0,877	0,89	0,985	160	121	150	120
YLR022C	SDO1	0,87	0,883	0,985	3	11	3	10
YGR202C	PCT1	0,867	0,88	0,985	7	11	10	10
YNL302C	RPS19B	0,864	0,877	0,985	6	6	6	6
YER089C	PTC2	0,909	0,923	0,985	19	16	20	17
YKL182W	FAS1	0,838	0,851	0,985	199	159	187	158
YOL077C	BRX1	0,832	0,845	0,985	19	20	21	23
YMR300C	ADE4	0,951	0,966	0,984	17	17	17	17
YFR016C	YFR016C	0,76	0,772	0,984	15	26	17	26
YML008C	ERG6	0,817	0,83	0,984	39	44	41	43
YMR311C	GLC8	0,744	0,756	0,984	14	15	16	16
YFR049W	YMR31	0,679	0,69	0,984	12	7	12	5
YKR095W-A	PCC1	0,861	0,875	0,984	4	4	4	4
YDL208W	NHP2	0,915	0,93	0,984	18	14	14	14
YER090W	TRP2	0,852	0,866	0,984	37	39	36	38
YPR104C	FHL1	0,827	0,841	0,983	2	2	2	4
YER023W	PRO3	0,881	0,896	0,983	37	30	36	30
YEL015W	EDC3	0,88	0,895	0,983	15	10	17	9
YER176W	ECM32	0,875	0,89	0,983	14	7	12	7
YNL216W	RAP1	0,812	0,826	0,983	15	17	13	17
YHR010W	RPL27A	0,868	0,883	0,983	92	44	90	44
YNL239W	LAP3	0,902	0,918	0,983	27	22	22	19
YLR223C	IFH1	0,393	0,4	0,983	2	3	1	5
YJR125C	ENT3	0,785	0,799	0,982	18	21	20	22
YOL127W	RPL25	0,894	0,91	0,982	76	53	75	48
YHR020W	YHR020W	0,89	0,906	0,982	80	67	74	70
YOR198C	BFR1	0,942	0,959	0,982	98	62	101	67
YGR089W	NNF2	0,771	0,785	0,982	3	2	2	1
YDL131W	LYS21	0,823	0,838	0,982	10	14	9	14
YOR171C	LCB4	0,656	0,668	0,982	7	6	7	6
YGL253W	HXK2	0,861	0,877	0,982	72	66	70	63

YOR216C	RUD3	0,858	0,874	0,982	7	17	9	19
YER021W	RPN3	0,801	0,816	0,982	19	18	20	20
YJR045C	SSC1	0,907	0,924	0,982	169	126	164	124
YMR011W	HXT2	0,32	0,326	0,982	2	1	2	1
YFR031C-A	RPL2A	0,851	0,867	0,982	157	116	155	118
YCR088W	ABP1	0,85	0,866	0,982	48	60	47	58
YKL061W	BLI1	0,739	0,753	0,981	1	4	2	5
YNL175C	NOP13	0,839	0,855	0,981	30	44	34	45
YDR356W	SPC110	0,785	0,8	0,981	14	23	11	23
YBR034C	HMT1	0,889	0,906	0,981	14	14	13	16
YJL128C	PBS2	0,782	0,797	0,981	14	10	11	9
YGL065C	ALG2	0,729	0,743	0,981	6	6	7	4
YDR368W	YPR1	0,829	0,845	0,981	19	29	25	29
YLR259C	HSP60	0,93	0,948	0,981	180	110	185	109
YOR310C	NOP58	0,878	0,895	0,981	60	47	59	50
YER125W	RSP5	0,825	0,841	0,981	12	15	13	16
YIL118W	RHO3	0,875	0,892	0,981	8	8	9	8
YHR179W	OYE2	0,772	0,787	0,981	35	39	32	37
YGR192C	TDH3	0,873	0,89	0,981	371	292	370	279
YBR079C	RPG1	0,87	0,887	0,981	123	116	126	107
YLR172C	DPH5	0,818	0,834	0,981	11	10	10	12
YER002W	NOP16	0,814	0,83	0,981	13	12	11	12
YER056C-A	RPL34A	0,863	0,88	0,981	70	47	71	45
YER048C	CAJ1	0,857	0,874	0,981	23	32	17	30
YBR191W	RPL21A	0,903	0,921	0,980	45	38	45	36
YKL014C	URB1	0,898	0,916	0,980	12	7	11	7
YNR046W	TRM112	0,847	0,864	0,980	3	4	3	4
YPR020W	ATP20	0,946	0,965	0,980	3	8	4	8
YOL086C	ADH1	0,846	0,863	0,980	189	146	177	148
YLR075W	RPL10	0,845	0,862	0,980	76	63	72	67
YJL131C	AIM23	0,889	0,907	0,980	3	6	4	6
YLR380W	CSR1	0,838	0,855	0,980	17	17	20	18
YLR447C	VMA6	0,786	0,802	0,980	6	10	6	10
YJL109C	UTP10	0,83	0,847	0,980	21	15	23	16
YGR112W	SHY1	0,926	0,945	0,980	5	7	4	6
YCL009C	ILV6	0,871	0,889	0,980	33	33	32	34
YOR224C	RPB8	0,87	0,888	0,980	5	7	5	6
YPR137W	RRP9	0,869	0,887	0,980	6	15	7	14
YDL166C	FAP7	0,856	0,874	0,979	6	8	6	8

YGL078C	DBP3	0,856	0,874	0,979	46	48	40	52
YHR007C	ERG11	0,375	0,383	0,979	26	24	24	22
YFR032C-A	RPL29	0,937	0,957	0,979	19	15	19	13
YDL178W	DLD2	0,883	0,902	0,979	35	26	35	28
YML111W	BUL2	0,836	0,854	0,979	5	3	4	2
YMR145C	NDE1	0,836	0,854	0,979	58	43	59	48
YIL036W	CST6	0,645	0,659	0,979	11	12	10	11
YFR028C	CDC14	0,826	0,844	0,979	6	8	5	8
YLR388W	RPS29A	0,868	0,887	0,979	33	28	31	30
YGL103W	RPL28	0,864	0,883	0,978	83	62	83	61
YNL147W	LSM7	0,953	0,974	0,978	2	4	2	4
YML094W	GIM5	0,86	0,879	0,978	18	12	14	13
YNL178W	RPS3	0,859	0,878	0,978	83	65	80	64
YHL038C	CBP2	0,948	0,969	0,978	5	3	4	3
YMR188C	MRPS17	0,902	0,922	0,978	12	12	11	12
YBR231C	SWC5	0,54	0,552	0,978	3	6	4	6
YGL148W	ARO2	0,855	0,874	0,978	47	55	47	50
YOR336W	KRE5	0,809	0,827	0,978	8	4	9	5
YBR127C	VMA2	0,807	0,825	0,978	75	68	75	76
YGL049C	TIF4632	0,848	0,867	0,978	29	26	25	27
YJR111C	YJR111C	0,801	0,819	0,978	4	3	4	2
YIL091C	UTP25	0,932	0,953	0,978	16	21	17	21
YGR264C	MES1	0,84	0,859	0,978	47	57	52	53
YGR136W	LSB1	0,482	0,493	0,978	5	11	5	10
YMR205C	PFK2	0,829	0,848	0,978	82	73	78	74
YBR084W	MIS1	0,869	0,889	0,978	37	53	34	52
YDR233C	RTN1	0,868	0,888	0,977	39	28	40	27
YBR196C	PGI1	0,867	0,887	0,977	112	79	112	73
YHR149C	SKG6	0,475	0,486	0,977	2	1	2	1
YOL040C	RPS15	0,862	0,882	0,977	71	60	79	59
YCR053W	THR4	0,857	0,877	0,977	69	67	67	63
YJR007W	SUI2	0,892	0,913	0,977	22	33	20	36
YMR079W	SEC14	0,892	0,913	0,977	32	34	28	31
YBR035C	PDX3	0,882	0,903	0,977	10	19	10	19
YDL066W	IDP1	0,878	0,899	0,977	62	61	62	62
YHR068W	DYS1	0,836	0,856	0,977	34	42	31	40
YPL196W	OXR1	0,708	0,725	0,977	2	1	2	1
YLR009W	RLP24	0,666	0,682	0,977	19	16	18	12
YML120C	NDI1	0,914	0,936	0,976	40	34	42	37

YDR148C	KGD2	0,896	0,918	0,976	21	22	24	20
YPR069C	SPE3	0,849	0,87	0,976	24	28	24	28
YML086C	ALO1	0,887	0,909	0,976	23	15	21	14
YNL274C	GOR1	0,921	0,944	0,976	17	18	16	18
YGR061C	ADE6	0,879	0,901	0,976	67	68	71	70
YPL145C	KES1	0,879	0,901	0,976	33	53	38	49
YDR028C	REG1	0,793	0,813	0,975	12	12	12	11
YIL041W	GVP36	0,905	0,928	0,975	62	58	63	59
YLR029C	RPL15A	0,862	0,884	0,975	67	60	63	57
YPL160W	CDC60	0,822	0,843	0,975	121	98	123	96
YKL081W	TEF4	0,86	0,882	0,975	79	76	79	72
YKR043C	YKR043C	0,816	0,837	0,975	9	13	8	11
YLR448W	RPL6B	0,891	0,914	0,975	70	50	70	47
YHR208W	BAT1	0,812	0,833	0,975	74	56	74	61
YDR189W	SLY1	0,888	0,911	0,975	15	19	16	21
YPL129W	TAF14	0,81	0,831	0,975	9	16	9	17
YJL069C	UTP18	0,809	0,83	0,975	10	13	9	14
YOR096W	RPS7A	0,885	0,908	0,975	101	80	105	71
YGL009C	LEU1	0,846	0,868	0,975	111	78	110	88
YKL085W	MDH1	0,922	0,946	0,975	57	40	59	46
YBR149W	ARA1	0,844	0,866	0,975	23	30	22	26
YDR450W	RPS18A	0,877	0,9	0,974	39	37	40	38
YER099C	PRS2	0,91	0,934	0,974	6	8	6	7
YNL157W	IGO1	0,755	0,775	0,974	12	10	12	10
YNL052W	COX5A	0,594	0,61	0,974	7	5	6	5
YLR148W	PEP3	0,776	0,797	0,974	4	4	3	5
YNL040W	YNL040W	0,886	0,91	0,974	6	7	6	8
YLR249W	YEF3	0,812	0,834	0,974	297	240	298	246
YDR069C	DOA4	0,659	0,677	0,973	5	3	4	2
YNL151C	RPC31	0,804	0,826	0,973	3	7	4	7
YOL057W	YOL057W	0,873	0,897	0,973	16	28	14	28
YEL044W	IES6	0,908	0,933	0,973	7	4	7	3
YCR043C	YCR043C	0,652	0,67	0,973	2	1	2	1
YGL238W	CSE1	0,796	0,818	0,973	7	4	8	6
YFL045C	SEC53	0,904	0,929	0,973	54	40	56	45
YLR195C	NMT1	0,864	0,888	0,973	18	28	19	28
YNL202W	SPS19	0,9	0,925	0,973	3	5	2	4
YIL022W	TIM44	0,931	0,957	0,973	32	33	31	32
YGL234W	ADE5,7	0,855	0,879	0,973	55	50	52	47

YBL064C	PRX1	0,921	0,947	0,973	15	17	16	13
YLL024C	SSA2	0,848	0,872	0,972	262	206	260	196
YCR012W	PGK1	0,879	0,904	0,972	267	177	255	173
YNL308C	KRI1	0,872	0,897	0,972	22	24	19	24
YHR183W	GND1	0,903	0,929	0,972	69	69	70	63
YMR186W	HSC82	0,861	0,886	0,972	139	123	149	124
YOR128C	ADE2	0,894	0,92	0,972	33	38	32	37
YLR286C	CTS1	0,48	0,494	0,972	2	5	3	5
YCL059C	KRR1	0,855	0,88	0,972	15	27	18	27
YNL135C	FPR1	0,887	0,913	0,972	28	20	29	22
YKL073W	LHS1	0,92	0,947	0,971	23	36	24	37
YPL239W	YAR1	0,816	0,84	0,971	17	16	16	18
YLR325C	RPL38	0,878	0,904	0,971	53	31	53	28
YBR263W	SHM1	0,877	0,903	0,971	57	57	54	53
YCR047C	BUD23	0,741	0,763	0,971	4	2	3	2
YGR234W	YHB1	0,804	0,828	0,971	70	62	73	60
YMR212C	EFR3	0,831	0,856	0,971	5	3	4	3
YOR317W	FAA1	0,93	0,958	0,971	41	46	39	44
YDR164C	SEC1	0,83	0,855	0,971	4	6	4	6
YMR012W	CLU1	0,857	0,883	0,971	65	87	62	82
YKL001C	MET14	0,888	0,915	0,970	33	27	35	27
YLR389C	STE23	0,886	0,913	0,970	14	19	15	17
YPR183W	DPM1	0,918	0,946	0,970	24	20	20	20
YGR032W	GSC2	0,686	0,707	0,970	1	1	1	1
YDR088C	SLU7	0,618	0,637	0,970	2	7	5	6
YKL080W	VMA5	0,811	0,836	0,970	38	33	35	35
YBR189W	RPS9B	0,871	0,898	0,970	70	79	70	75
YOR063W	RPL3	0,869	0,896	0,970	177	136	187	132
YJR009C	TDH2	0,802	0,827	0,970	34	28	38	34
YKL211C	TRP3	0,865	0,892	0,970	31	34	29	34
YDL112W	TRM3	0,863	0,89	0,970	10	9	11	11
YHR049W	FSH1	0,892	0,92	0,970	34	31	28	32
YOR091W	TMA46	0,792	0,817	0,969	17	24	17	24
YER164W	CHD1	0,755	0,779	0,969	23	14	22	15
YIL078W	THS1	0,874	0,902	0,969	49	58	46	58
YJR075W	HOC1	0,684	0,706	0,969	6	4	6	4
YJR060W	CBF1	0,807	0,833	0,969	16	17	14	15
YCR073W-A	SOL2	0,837	0,864	0,969	8	8	7	8
YPR181C	SEC23	0,831	0,858	0,969	23	21	21	23

YLR290C	YLR290C	0,922	0,952	0,968	2	2	1	2
YPR088C	SRP54	0,767	0,792	0,968	17	27	16	24
YBR177C	EHT1	0,859	0,887	0,968	20	16	18	19
YJL121C	RPE1	0,859	0,887	0,968	22	26	27	24
YNL007C	SIS1	0,825	0,852	0,968	22	31	21	31
YMR067C	UBX4	0,791	0,817	0,968	16	16	15	17
YGR123C	PPT1	0,845	0,873	0,968	7	14	11	15
YLL045C	RPL8B	0,871	0,9	0,968	128	96	131	94
YGR222W	PET54	0,931	0,962	0,968	1	1	1	1
YKL172W	EBP2	0,9	0,93	0,968	34	28	33	30
YOR356W	YOR356W	0,925	0,956	0,968	21	28	19	28
YNL134C	YNL134C	0,894	0,924	0,968	38	34	41	37
YOR142W	LSC1	0,921	0,952	0,967	44	43	43	37
YKR071C	DRE2	0,412	0,426	0,967	24	22	28	25
YGL097W	SRM1	0,912	0,943	0,967	9	18	10	16
YOL145C	CTR9	0,966	0,999	0,967	15	14	14	14
YGR178C	PBP1	0,847	0,876	0,967	36	39	35	39
YGL173C	KEM1	0,872	0,902	0,967	61	54	58	57
YJR117W	STE24	0,887	0,918	0,966	5	4	6	6
YHL015W	RPS20	0,885	0,916	0,966	51	34	50	33
YOR259C	RPT4	0,852	0,882	0,966	26	24	25	24
YDR367W	KEI1	0,88	0,911	0,966	1	1	1	1
YPR041W	TIF5	0,879	0,91	0,966	57	52	59	53
YDR304C	CPR5	0,878	0,909	0,966	13	19	11	19
YML093W	UTP14	0,679	0,703	0,966	16	22	18	23
YDR111C	ALT2	0,933	0,966	0,966	2	3	3	3
YBL076C	ILS1	0,845	0,875	0,966	71	63	74	67
YNL122C	YNL122C	0,752	0,779	0,965	3	3	5	3
YCR005C	CIT2	0,362	0,375	0,965	7	15	6	15
YER043C	SAH1	0,888	0,92	0,965	93	61	100	58
YOR001W	RRP6	0,775	0,803	0,965	18	20	21	20
YMR203W	TOM40	0,911	0,944	0,965	24	24	22	23
YPR125W	YLH47	0,855	0,886	0,965	7	9	8	10
YCR030C	SYP1	0,854	0,885	0,965	19	20	20	23
YDL229W	SSB1	0,853	0,884	0,965	311	206	303	197
YGL123W	RPS2	0,851	0,882	0,965	111	73	110	69
YLL031C	GPI13	0,711	0,737	0,965	4	2	3	2
YGR285C	ZUO1	0,869	0,901	0,964	61	49	59	46
YGR162W	TIF4631	0,86	0,892	0,964	62	53	57	55

YGL026C	TRP5	0,806	0,836	0,964	78	82	79	83
YKL214C	YRA2	0,806	0,836	0,964	13	9	7	10
YPL271W	ATP15	0,831	0,862	0,964	16	8	14	9
YGL012W	ERG4	0,96	0,996	0,964	3	2	3	3
YOL059W	GPD2	0,744	0,772	0,964	19	18	21	20
YOR143C	THI80	0,929	0,964	0,964	4	6	4	7
YJR002W	MPP10	0,796	0,826	0,964	29	28	25	25
YBL099W	ATP1	0,899	0,933	0,964	85	69	83	65
YMR120C	ADE17	0,846	0,878	0,964	60	61	55	62
YOL066C	RIB2	0,739	0,767	0,963	4	6	2	5
YPL260W	YPL260W	0,868	0,901	0,963	20	24	23	23
YOR232W	MGE1	0,89	0,924	0,963	46	38	46	37
YPR110C	RPC40	0,837	0,869	0,963	30	22	28	21
YNL061W	NOP2	0,863	0,896	0,963	27	32	25	34
YKL204W	EAP1	0,832	0,864	0,963	28	26	27	23
YJL172W	CPS1	0,879	0,913	0,963	15	10	13	12
YMR083W	ADH3	0,871	0,905	0,962	23	29	23	26
YLL001W	DNM1	0,868	0,902	0,962	19	29	23	33
YDL092W	SRP14	0,866	0,9	0,962	10	11	9	11
YNL301C	RPL18B	0,865	0,899	0,962	74	73	65	72
YLR287C-A	RPS30A	0,913	0,949	0,962	19	16	21	16
YER094C	PUP3	0,81	0,842	0,962	7	11	7	10
YJR123W	RPS5	0,885	0,92	0,962	61	53	60	49
YPL093W	NOG1	0,656	0,682	0,962	46	45	47	44
YIL020C	HIS6	0,83	0,863	0,962	7	12	7	11
YMR116C	ASC1	0,83	0,863	0,962	95	86	102	93
YDL078C	MDH3	0,88	0,915	0,962	22	25	19	24
YER006W	NUG1	0,804	0,836	0,962	41	45	42	45
YEL002C	WBP1	0,677	0,704	0,962	5	7	6	7
YGL147C	RPL9A	0,851	0,885	0,962	62	45	61	46
YBR248C	HIS7	0,9	0,936	0,962	30	40	32	42
YKL143W	LTV1	0,899	0,935	0,961	10	11	10	9
YKL033W-A	YKL033W-A	0,845	0,879	0,961	13	18	13	19
YFL005W	SEC4	0,844	0,878	0,961	11	16	12	15
YBR268W	MRPL37	0,818	0,851	0,961	2	5	2	4
YHR196W	UTP9	0,864	0,899	0,961	7	15	11	14
YLL008W	DRS1	0,861	0,896	0,961	19	29	17	28
YJR016C	ILV3	0,81	0,843	0,961	86	88	93	90
YJL138C	TIF2	0,856	0,891	0,961	90	90	91	89

YDL115C	IWR1	0,635	0,661	0,961	4	6	4	6
YPL225W	YPL225W	0,875	0,911	0,960	14	14	15	14
YDR382W	RPP2B	0,802	0,835	0,960	42	42	41	40
YCL045C	EMC1	0,848	0,883	0,960	6	7	6	7
YPL048W	CAM1	0,848	0,883	0,960	17	23	19	22
YNL087W	TCB2	0,847	0,882	0,960	15	13	16	12
YOL077W-A	ATP19	0,435	0,453	0,960	6	4	6	4
YJR144W	MGM101	0,845	0,88	0,960	16	20	14	18
YDR127W	ARO1	0,869	0,905	0,960	68	83	70	84
YLR089C	ALT1	0,724	0,754	0,960	18	26	18	27
YKL060C	FBA1	0,843	0,878	0,960	299	232	309	234
YOL121C	RPS19A	0,867	0,903	0,960	95	61	100	63
YJR077C	MIR1	0,914	0,952	0,960	35	30	37	31
YPL009C	TAE2	0,862	0,898	0,960	10	14	8	14
YIL074C	SER33	0,861	0,897	0,960	38	42	39	42
YOL139C	CDC33	0,902	0,94	0,960	24	24	24	27
YBR025C	OLA1	0,829	0,864	0,959	48	48	47	47
YFR052W	RPN12	0,876	0,913	0,959	13	17	12	16
YBR080C	SEC18	0,826	0,861	0,959	28	35	29	37
YBR218C	PYC2	0,825	0,86	0,959	16	14	15	15
YPR108W	RPN7	0,872	0,909	0,959	16	18	13	19
YDL236W	PHO13	0,87	0,907	0,959	17	20	21	21
YOR209C	NPT1	0,87	0,907	0,959	33	33	36	30
YPL004C	LSP1	0,799	0,833	0,959	56	47	54	45
YKL082C	RRP14	0,822	0,857	0,959	16	22	19	19
YDR238C	SEC26	0,796	0,83	0,959	23	20	26	19
YER073W	ALD5	0,819	0,854	0,959	58	60	48	58
YIL053W	RHR2	0,912	0,951	0,959	99	73	96	70
YPR029C	APL4	0,768	0,801	0,959	11	7	12	7
YNL162W	RPL42A	0,906	0,945	0,959	42	29	34	25
YBR286W	APE3	0,813	0,848	0,959	51	43	50	39
YPR143W	RRP15	0,808	0,843	0,958	12	11	14	10
YJL005W	CYR1	0,738	0,77	0,958	5	7	6	7
YMR189W	GCV2	0,803	0,838	0,958	27	38	27	36
YBR006W	UGA2	0,87	0,908	0,958	4	6	4	5
YJL020C	BBC1	0,8	0,835	0,958	32	45	34	47
YDL198C	GGC1	0,89	0,929	0,958	21	25	21	24
YER159C	BUR6	0,772	0,806	0,958	4	2	3	2
YGR211W	ZPR1	0,84	0,877	0,958	23	30	17	31

YER003C	PMI40	0,884	0,923	0,958	52	50	53	50
YOR354C	MSC6	0,906	0,946	0,958	17	20	14	20
YHR174W	ENO2	0,859	0,897	0,958	393	276	383	255
YGR214W	RPS0A	0,836	0,873	0,958	19	18	17	18
YOL123W	HRP1	0,813	0,849	0,958	15	19	15	20
YHR117W	TOM71	0,903	0,943	0,958	12	15	11	16
YDL103C	QRI1	0,856	0,894	0,957	32	41	32	38
YOR293W	RPS10A	0,875	0,914	0,957	33	20	31	19
YOR133W	EFT1	0,85	0,888	0,957	212	164	210	175
YBR031W	RPL4A	0,849	0,887	0,957	179	132	184	131
YGR054W	YGR054W	0,847	0,885	0,957	38	41	37	43
YLR441C	RPS1A	0,891	0,931	0,957	27	15	24	15
YOR286W	RDL2	0,868	0,907	0,957	19	15	19	20
YLR191W	PEX13	0,488	0,51	0,957	1	3	2	4
YJL200C	ACO2	0,838	0,876	0,957	36	49	36	46
YDR190C	RVB1	0,857	0,896	0,956	34	35	33	39
YER156C	YER156C	0,878	0,918	0,956	17	24	17	24
YDR486C	VPS60	0,812	0,849	0,956	4	5	4	3
YPL226W	NEW1	0,809	0,846	0,956	57	70	57	72
YGR167W	CLC1	0,807	0,844	0,956	21	27	24	29
YFR030W	MET10	0,87	0,91	0,956	107	84	107	83
YGR155W	CYS4	0,848	0,887	0,956	119	72	130	74
YER052C	HOM3	0,847	0,886	0,956	38	38	41	36
YPL173W	MRPL40	0,954	0,998	0,956	19	13	25	23
YML070W	DAK1	0,844	0,883	0,956	31	42	31	37
YNL124W	NAF1	0,649	0,679	0,956	4	6	4	5
YBR172C	SMY2	0,735	0,769	0,956	15	22	16	23
YER081W	SER3	0,925	0,968	0,956	13	12	13	11
YNL241C	ZWF1	0,881	0,922	0,956	50	61	50	60
YDR212W	TCP1	0,858	0,898	0,955	22	22	22	22
YDR388W	RVS167	0,81	0,848	0,955	18	20	20	18
YNL044W	YIP3	0,785	0,822	0,955	8	10	6	8
YBR265W	TSC10	0,763	0,799	0,955	2	2	1	2
YDR299W	BFR2	0,826	0,865	0,955	15	18	15	21
YBR011C	IPP1	0,868	0,909	0,955	84	67	85	68
YBL030C	PET9	0,91	0,953	0,955	37	38	43	35
YNL177C	MRPL22	0,888	0,93	0,955	9	12	7	10
YGL195W	GCN1	0,803	0,841	0,955	44	37	42	39
YMR031C	YMR031C	0,8	0,838	0,955	72	69	70	63

YKL155C	RSM22	0,841	0,881	0,955	14	9	14	9
YHR076W	PTC7	0,862	0,903	0,955	15	18	14	17
YDR158W	HOM2	0,861	0,902	0,955	72	59	77	59
YGL008C	PMA1	0,88	0,922	0,954	143	75	145	81
YPR004C	AIM45	0,919	0,963	0,954	19	22	18	22
YER102W	RPS8B	0,852	0,893	0,954	89	57	88	52
YCL017C	NFS1	0,747	0,783	0,954	5	10	7	13
YKR095W	MLP1	0,788	0,826	0,954	37	45	32	42
YOR375C	GDH1	0,829	0,869	0,954	92	71	92	70
YGL099W	LSG1	0,828	0,868	0,954	23	25	24	26
YOR267C	HRK1	0,765	0,802	0,954	17	15	19	14
YLR060W	FRS1	0,868	0,91	0,954	50	58	53	56
YDR391C	YDR391C	0,909	0,953	0,954	4	5	4	6
YOR260W	GCD1	0,805	0,844	0,954	6	6	6	6
YDL063C	YDL063C	0,908	0,952	0,954	2	2	4	2
YBR015C	MNN2	0,818	0,858	0,953	18	16	17	15
YGR037C	ACB1	0,838	0,879	0,953	14	17	15	18
YLR044C	PDC1	0,817	0,857	0,953	270	244	284	247
YLR355C	ILV5	0,856	0,898	0,953	119	102	119	100
YDL135C	RDI1	0,835	0,876	0,953	11	13	11	12
YOR007C	SGT2	0,832	0,873	0,953	47	42	44	42
YKL088W	CAB3	0,932	0,978	0,953	5	11	4	11
YBL050W	SEC17	0,828	0,869	0,953	14	20	16	18
YHR008C	SOD2	0,806	0,846	0,953	21	23	21	25
YDR165W	TRM82	0,823	0,864	0,953	16	27	15	29
YNL141W	AAH1	0,841	0,883	0,952	25	24	24	24
YPR118W	MRI1	0,879	0,923	0,952	16	22	19	21
YHR019C	DED81	0,855	0,898	0,952	42	51	41	50
YOR298C-A	MBF1	0,754	0,792	0,952	55	43	54	42
YDR071C	PAA1	0,813	0,854	0,952	28	25	27	25
YBR039W	ATP3	0,926	0,973	0,952	45	37	40	35
YOR020W-A	YOR020W-A	0,886	0,931	0,952	3	3	2	2
YDR119W	VBA4	0,883	0,928	0,952	3	4	4	2
YLR084C	RAX2	0,921	0,968	0,951	2	3	2	3
YGL145W	TIP20	0,742	0,78	0,951	5	4	6	6
YDR174W	HMO1	0,835	0,878	0,951	30	22	29	23
YBR171W	SEC66	0,912	0,959	0,951	4	7	4	6
YEL022W	GEA2	0,834	0,877	0,951	9	6	12	6
YOR239W	ABP140	0,832	0,875	0,951	13	23	13	22

YOR311C	DGK1	0,753	0,792	0,951	5	2	4	1
YFR033C	QCR6	0,924	0,972	0,951	14	12	16	12
YJR104C	SOD1	0,749	0,788	0,951	43	24	39	24
YDL001W	RMD1	0,824	0,867	0,950	3	1	2	2
YER078C	ICP55	0,822	0,865	0,950	3	9	2	10
YPR145W	ASN1	0,822	0,865	0,950	63	72	69	69
YML073C	RPL6A	0,879	0,925	0,950	25	17	26	19
YKL191W	DPH2	0,916	0,964	0,950	7	11	5	11
YMR290C	HAS1	0,819	0,862	0,950	18	29	18	31
YIL043C	CBR1	0,857	0,902	0,950	11	16	15	17
YNL085W	MKT1	0,835	0,879	0,950	15	17	15	17
YGR094W	VAS1	0,85	0,895	0,950	80	76	83	76
YCL037C	SRO9	0,792	0,834	0,950	42	31	42	33
YLR340W	RPP0	0,865	0,911	0,950	65	60	67	61
YDR345C	HXT3	0,747	0,787	0,949	6	9	7	9
YJR010W	MET3	0,82	0,864	0,949	55	52	59	52
YAL035W	FUN12	0,857	0,903	0,949	53	54	51	56
YDR312W	SSF2	0,836	0,881	0,949	11	14	12	15
YMR276W	DSK2	0,685	0,722	0,949	6	10	9	10
YNL069C	RPL16B	0,851	0,897	0,949	101	74	106	74
YPR167C	MET16	0,684	0,721	0,949	16	20	17	19
YMR314W	PRE5	0,868	0,915	0,949	22	25	23	21
YER149C	PEA2	0,794	0,837	0,949	12	12	16	13
YDR419W	RAD30	0,903	0,952	0,949	1	1	1	1
YGR181W	TIM13	0,866	0,913	0,949	15	11	13	10
YLL023C	POM33	0,68	0,717	0,948	1	1	1	1
YER165W	PAB1	0,863	0,91	0,948	105	96	111	97
YGL043W	DST1	0,878	0,926	0,948	21	19	21	17
YJL184W	GON7	0,768	0,81	0,948	2	3	3	3
YDL060W	TSR1	0,877	0,925	0,948	16	14	14	15
YMR308C	PSE1	0,893	0,942	0,948	15	17	16	17
YAR002W	NUP60	0,563	0,594	0,948	28	23	29	23
YLL018C	DPS1	0,835	0,881	0,948	59	58	58	59
YPL237W	SUI3	0,889	0,938	0,948	39	53	35	48
YOR116C	RPO31	0,688	0,726	0,948	12	13	12	12
YHR192W	YHR192W	0,866	0,914	0,947	2	4	2	4
YKL056C	TMA19	0,847	0,894	0,947	39	30	37	30
YBR289W	SNF5	0,682	0,72	0,947	3	3	2	3
YFR002W	NIC96	0,824	0,87	0,947	16	16	15	18

YGR063C	SPT4	0,716	0,756	0,947	3	3	4	3
YOR167C	RPS28A	0,821	0,867	0,947	25	14	27	15
YHR146W	CRP1	0,799	0,844	0,947	23	31	25	31
YDL084W	SUB2	0,852	0,9	0,947	39	42	40	42
YPR089W	YPR089W	0,744	0,786	0,947	1	1	1	1
YJL014W	CCT3	0,849	0,897	0,946	33	46	35	41
YHL033C	RPL8A	0,884	0,934	0,946	26	18	24	17
YER020W	GPA2	0,936	0,989	0,946	1	1	1	1
YGR086C	PIL1	0,812	0,858	0,946	67	57	68	55
YDR086C	SSS1	0,792	0,837	0,946	6	2	6	3
YJL041W	NSP1	0,827	0,874	0,946	60	60	67	60
YIL109C	SEC24	0,826	0,873	0,946	13	20	16	21
YPR074C	TKL1	0,861	0,91	0,946	101	76	100	84
YLR276C	DBP9	0,86	0,909	0,946	20	25	19	26
YGL172W	NUP49	0,77	0,814	0,946	7	7	7	7
YPL116W	HOS3	0,802	0,848	0,946	8	15	9	12
YGL202W	ARO8	0,836	0,884	0,946	60	55	59	58
YDR226W	ADK1	0,883	0,934	0,945	91	80	90	82
YLR309C	IMH1	0,812	0,859	0,945	26	31	24	32
YGL157W	ARI1	0,915	0,968	0,945	26	23	23	23
YEL058W	PCM1	0,894	0,946	0,945	14	20	15	19
YGR193C	PDX1	0,859	0,909	0,945	25	29	25	36
YPL090C	RPS6A	0,857	0,907	0,945	116	70	113	73
YLR025W	SNF7	0,889	0,941	0,945	3	10	4	10
YFL039C	ACT1	0,836	0,885	0,945	76	54	74	61
YNL255C	GIS2	0,836	0,885	0,945	15	16	16	18
YKL094W	YJU3	0,852	0,902	0,945	9	13	9	12
YLR277C	YSH1	0,766	0,811	0,945	2	3	3	3
YAR042W	SWH1	0,834	0,883	0,945	10	8	10	5
YDL126C	CDC48	0,779	0,825	0,944	82	86	87	84
YBR089C-A	NHP6B	0,863	0,914	0,944	6	2	4	4
YDL007W	RPT2	0,778	0,824	0,944	28	28	27	28
YHR040W	BCD1	0,71	0,752	0,944	2	3	3	3
YDR377W	ATP17	0,741	0,785	0,944	8	5	8	4
YLR058C	SHM2	0,857	0,908	0,944	125	93	124	89
YNL108C	YNL108C	0,839	0,889	0,944	11	20	10	16
YBR227C	MCX1	0,905	0,959	0,944	16	21	15	20
YOL064C	MET22	0,871	0,923	0,944	26	26	24	26
YDR064W	RPS13	0,868	0,92	0,943	51	30	49	33

YPR163C	TIF3	0,868	0,92	0,943	41	33	42	31
YBR106W	PHO88	0,817	0,866	0,943	19	17	16	18
YDR298C	ATP5	0,881	0,934	0,943	32	25	33	24
YPL111W	CAR1	0,795	0,843	0,943	39	36	42	34
YMR241W	YHM2	0,828	0,878	0,943	10	14	10	14
YOR086C	TCB1	0,875	0,928	0,943	25	9	24	13
YNL259C	ATX1	0,792	0,84	0,943	3	4	3	4
YER063W	THO1	0,84	0,891	0,943	15	16	15	14
YNL245C	CWC25	0,609	0,646	0,943	2	3	2	3
YDL081C	RPP1A	0,834	0,885	0,942	27	24	24	25
YJR064W	CCT5	0,85	0,902	0,942	53	61	53	59
YDR335W	MSN5	0,817	0,867	0,942	2	1	2	1
YIL125W	KGD1	0,882	0,936	0,942	25	38	25	37
YFR010W	UBP6	0,815	0,865	0,942	25	30	23	26
YIL105C	SLM1	0,861	0,914	0,942	6	6	6	7
YAL041W	CDC24	0,682	0,724	0,942	3	6	3	7
YDR035W	ARO3	0,893	0,948	0,942	44	38	36	37
YLR354C	TAL1	0,892	0,947	0,942	50	52	49	48
YNL292W	PUS4	0,826	0,877	0,942	8	14	8	12
YOR095C	RKI1	0,824	0,875	0,942	20	23	23	23
YJR137C	MET5	0,806	0,856	0,942	186	170	185	163
YDR152W	GIR2	0,846	0,899	0,941	9	10	10	9
YER036C	ARB1	0,844	0,897	0,941	51	47	54	43
YGL027C	CWH41	0,78	0,829	0,941	7	8	10	6
YNL267W	PIK1	0,923	0,981	0,941	4	4	3	5
YML075C	HMG1	0,811	0,862	0,941	20	12	21	11
YLR057W	YLR057W	0,824	0,876	0,941	5	4	4	4
YMR102C	YMR102C	0,839	0,892	0,941	2	2	2	1
YMR235C	RNA1	0,823	0,875	0,941	28	28	26	29
YEL054C	RPL12A	0,838	0,891	0,941	77	56	78	52
YAL003W	EFB1	0,836	0,889	0,940	78	67	74	64
YNL002C	RLP7	0,741	0,788	0,940	27	28	32	27
YIL009W	FAA3	0,772	0,821	0,940	7	3	7	3
YIL063C	YRB2	0,85	0,904	0,940	8	15	6	13
YIR012W	SQT1	0,865	0,92	0,940	6	12	8	12
YPL023C	MET12	0,8	0,851	0,940	4	12	3	12
YBR143C	SUP45	0,847	0,901	0,940	51	52	45	50
YFL037W	TUB2	0,878	0,934	0,940	19	23	22	19
YGR218W	CRM1	0,845	0,899	0,940	21	14	20	15

YIL051C	MMF1	0,829	0,882	0,940	41	28	42	28
YOR301W	RAX1	0,61	0,649	0,940	1	2	1	2
YER166W	DNF1	0,795	0,846	0,940	7	11	8	10
YPL079W	RPL21B	0,872	0,928	0,940	6	6	6	6
YDR517W	GRH1	0,794	0,845	0,940	10	4	8	4
YOR341W	RPA190	0,746	0,794	0,940	81	78	73	75
YDL095W	PMT1	0,791	0,842	0,939	13	9	13	12
YDL014W	NOP1	0,822	0,875	0,939	57	52	58	50
YLR429W	CRN1	0,822	0,875	0,939	53	55	64	54
YLR008C	PAM18	0,853	0,908	0,939	7	8	8	7
YOR283W	YOR283W	0,853	0,908	0,939	7	9	7	10
YDL029W	ARP2	0,834	0,888	0,939	20	23	18	19
YCL034W	LSB5	0,787	0,838	0,939	3	8	6	7
YHR070W	TRM5	0,845	0,9	0,939	6	11	6	12
YNL138W	SRV2	0,86	0,916	0,939	29	39	29	38
YBR121C	GRS1	0,888	0,946	0,939	97	93	89	83
YPL127C	HHO1	0,459	0,489	0,939	25	20	26	22
YAL012W	CYS3	0,838	0,893	0,938	58	53	63	51
YPR139C	VPS66	0,806	0,859	0,938	4	3	5	4
YDR428C	BNA7	0,851	0,907	0,938	1	2	1	2
YDL051W	LHP1	0,775	0,826	0,938	23	24	20	24
YOR326W	MYO2	0,789	0,841	0,938	34	27	25	27
YDR297W	SUR2	0,773	0,824	0,938	5	4	4	4
YDR083W	RRP8	0,893	0,952	0,938	9	8	11	7
YLL041C	SDH2	0,798	0,851	0,938	12	14	9	13
YLR216C	CPR6	0,843	0,899	0,938	30	36	30	38
YGL106W	MLC1	0,794	0,847	0,937	13	16	11	14
YBR249C	ARO4	0,836	0,892	0,937	60	58	59	60
YER178W	PDA1	0,806	0,86	0,937	24	38	24	41
YBL039C	URA7	0,805	0,859	0,937	45	52	47	56
YBL058W	SHP1	0,775	0,827	0,937	18	26	17	22
YJR131W	MNS1	0,596	0,636	0,937	2	2	3	2
YDL083C	RPS16B	0,878	0,937	0,937	65	49	69	53
YML048W	GSF2	0,815	0,87	0,937	20	14	19	13
YGL023C	PIB2	0,74	0,79	0,937	7	10	5	9
YDR465C	RMT2	0,799	0,853	0,937	6	7	7	7
YPL169C	MEX67	0,807	0,862	0,936	10	15	6	14
YKL077W	YKL077W	0,803	0,858	0,936	6	10	6	11
YER092W	IES5	0,613	0,655	0,936	7	7	7	8

YCR031C	RPS14A	0,817	0,873	0,936	95	78	95	76
YJR025C	BNA1	0,874	0,934	0,936	6	10	5	10
YLR382C	NAM2	0,814	0,87	0,936	4	3	4	3
YNL104C	LEU4	0,813	0,869	0,936	57	46	63	45
YMR217W	GUA1	0,856	0,915	0,936	57	55	56	53
YOR027W	STI1	0,826	0,883	0,935	99	85	102	81
YJR139C	HOM6	0,854	0,913	0,935	78	67	80	65
YHR064C	SSZ1	0,849	0,908	0,935	75	68	78	71
YML035C	AMD1	0,906	0,969	0,935	13	11	10	10
YNL154C	YCK2	0,733	0,784	0,935	8	6	9	7
YER057C	HMF1	0,818	0,875	0,935	27	22	26	25
YLR048W	RPS0B	0,803	0,859	0,935	49	37	50	34
YDR023W	SES1	0,845	0,904	0,935	62	60	67	55
YGR240C	PFK1	0,801	0,857	0,935	109	90	114	98
YLR175W	CBF5	0,813	0,87	0,934	28	31	34	33
YCL030C	HIS4	0,85	0,91	0,934	104	91	101	92
YMR267W	PPA2	0,906	0,97	0,934	5	13	8	14
YLR406C	RPL31B	0,762	0,816	0,934	4	4	4	4
YNL248C	RPA49	0,832	0,891	0,934	37	32	37	25
YDR429C	TIF35	0,845	0,905	0,934	15	15	15	17
YOL149W	DCP1	0,731	0,783	0,934	3	6	4	6
YJL154C	VPS35	0,829	0,888	0,934	3	1	3	1
YPL091W	GLR1	0,871	0,933	0,934	31	40	26	40
YOR261C	RPN8	0,771	0,826	0,933	27	17	30	19
YPL161C	BEM4	0,855	0,916	0,933	5	2	4	1
YHR199C	AIM46	0,588	0,63	0,933	3	6	3	6
YDR011W	SNQ2	0,696	0,746	0,933	4	4	4	5
YLR200W	YKE2	0,848	0,909	0,933	11	12	12	13
YLR219W	MSC3	0,834	0,894	0,933	17	13	17	14
YMR214W	SCJ1	0,874	0,937	0,933	8	12	7	14
YDL055C	PSA1	0,818	0,877	0,933	71	60	72	59
YLR179C	YLR179C	0,803	0,861	0,933	15	17	18	17
YDR060W	MAK21	0,854	0,916	0,932	19	29	21	27
YBL031W	SHE1	0,537	0,576	0,932	1	2	1	1
YMR307W	GAS1	0,81	0,869	0,932	31	20	31	24
YPL115C	BEM3	0,712	0,764	0,932	8	7	7	7
YKR016W	FCJ1	0,862	0,925	0,932	18	30	16	30
YJL180C	ATP12	0,833	0,894	0,932	5	7	5	8
YMR080C	NAM7	0,844	0,906	0,932	9	12	10	15

YNL132W	KRE33	0,83	0,891	0,932	24	23	22	23
YPR171W	BSP1	0,76	0,816	0,931	6	5	5	3
YOR271C	FSF1	0,881	0,946	0,931	10	10	9	10
YHR193C	EGD2	0,867	0,931	0,931	62	51	62	46
YPR010C	RPA135	0,77	0,827	0,931	40	30	39	30
YHR063C	PAN5	0,797	0,856	0,931	15	19	17	19
YEL055C	POL5	0,851	0,914	0,931	13	11	14	10
YCL018W	LEU2	0,782	0,84	0,931	90	81	87	82
YIL005W	EPS1	0,647	0,695	0,931	8	5	8	5
YDL153C	SAS10	0,795	0,854	0,931	19	14	19	13
YER070W	RNR1	0,484	0,52	0,931	26	26	26	26
YIL021W	RPB3	0,819	0,88	0,931	17	13	15	15
YDL004W	ATP16	0,872	0,937	0,931	30	25	31	23
YDL143W	CCT4	0,803	0,863	0,930	26	28	23	28
YPL249C	GYP5	0,814	0,875	0,930	7	6	7	5
YAL049C	AIM2	0,827	0,889	0,930	7	13	5	11
YBR280C	SAF1	0,879	0,945	0,930	1	1	1	1
YOL098C	YOL098C	0,785	0,844	0,930	23	21	21	22
YLR197W	NOP56	0,838	0,901	0,930	64	67	68	63
YJL008C	CCT8	0,823	0,885	0,930	51	54	44	50
YPL198W	RPL7B	0,874	0,94	0,930	10	4	11	3
YBR222C	PCS60	0,794	0,854	0,930	18	19	15	22
YGR270W	YTA7	0,807	0,868	0,930	5	7	6	7
YKL104C	GFA1	0,873	0,939	0,930	29	37	27	32
YJL071W	ARG2	0,845	0,909	0,930	5	4	4	2
YNL240C	NAR1	0,871	0,937	0,930	2	2	4	2
YPL217C	BMS1	0,8	0,861	0,929	21	28	24	24
YJR017C	ESS1	0,869	0,936	0,928	9	16	7	16
YER082C	UTP7	0,803	0,865	0,928	17	23	20	25
YPR060C	ARO7	0,851	0,917	0,928	11	18	10	16
YJL082W	IML2	0,696	0,75	0,928	1	2	2	2
YHR103W	SBE22	0,296	0,319	0,928	2	1	3	1
YMR297W	PRC1	0,744	0,802	0,928	13	25	14	24
YLR061W	RPL22A	0,87	0,938	0,928	15	13	17	13
YIL133C	RPL16A	0,844	0,91	0,927	49	33	51	29
YNL168C	FMP41	0,818	0,882	0,927	10	11	10	12
YNR043W	MVD1	0,816	0,88	0,927	24	23	24	22
YGL120C	PRP43	0,851	0,918	0,927	18	21	19	16
YOR168W	GLN4	0,824	0,889	0,927	52	67	46	68

YDR293C	SSD1	0,835	0,901	0,927	26	32	31	33
YPL151C	PRP46	0,759	0,819	0,927	1	2	1	3
YHL013C	OTU2	0,859	0,927	0,927	3	6	2	5
YBR111C	YSA1	0,896	0,967	0,927	17	11	18	14
YKL018W	SWD2	0,792	0,855	0,926	8	11	7	8
YOR196C	LIP5	0,665	0,718	0,926	10	11	9	11
YPL240C	HSP82	0,765	0,826	0,926	10	13	11	15
YMR091C	NPL6	0,802	0,866	0,926	12	21	12	22
YBL001C	ECM15	0,764	0,825	0,926	4	7	6	6
YNL071W	LAT1	0,889	0,96	0,926	51	40	55	36
YGL048C	RPT6	0,801	0,865	0,926	33	35	34	33
YLR208W	SEC13	0,8	0,864	0,926	17	18	16	18
YOR335C	ALA1	0,799	0,863	0,926	89	76	82	75
YPR024W	YME1	0,699	0,755	0,926	12	15	13	14
YMR049C	ERB1	0,848	0,916	0,926	31	35	31	37
YHR065C	RRP3	0,81	0,875	0,926	14	14	10	14
YJL117W	PHO86	0,893	0,965	0,925	2	2	2	1
YDR508C	GNP1	0,347	0,375	0,925	5	5	5	5
YIR036C	IRC24	0,878	0,949	0,925	6	10	9	9
YLR384C	IKI3	0,853	0,922	0,925	21	20	24	24
YHL039W	EFM1	0,865	0,935	0,925	5	3	4	2
YNR050C	LYS9	0,852	0,921	0,925	35	43	32	38
YLR192C	HCR1	0,753	0,814	0,925	31	34	32	33
YIL034C	CAP2	0,739	0,799	0,925	5	5	6	6
YLR287C	YLR287C	0,874	0,945	0,925	4	3	2	3
YBR088C	POL30	0,836	0,904	0,925	17	27	17	27
YAL044C	GCV3	0,811	0,877	0,925	13	12	12	13
YPL262W	FUM1	0,857	0,927	0,924	34	36	28	34
YDL226C	GCS1	0,832	0,9	0,924	24	31	28	33
YDR321W	ASP1	0,832	0,9	0,924	15	20	15	20
YLR304C	ACO1	0,78	0,844	0,924	103	104	108	109
YGR152C	RSR1	0,742	0,803	0,924	5	3	5	3
YHR107C	CDC12	0,869	0,941	0,923	24	22	26	23
YOR122C	PFY1	0,816	0,884	0,923	16	19	19	15
YNL268W	LYP1	0,406	0,44	0,923	4	3	6	1
YKL134C	OCT1	0,692	0,75	0,923	3	1	3	2
YGL030W	RPL30	0,87	0,943	0,923	23	27	22	27
YIL062C	ARC15	0,834	0,904	0,923	4	6	5	6
YAL016W	TPD3	0,831	0,901	0,922	13	17	9	15

YDL061C	RPS29B	0,83	0,9	0,922	32	20	30	21
YKR062W	TFA2	0,651	0,706	0,922	7	15	8	16
YER029C	SMB1	0,722	0,783	0,922	4	5	4	6
YNL224C	SQS1	0,731	0,793	0,922	2	1	3	2
YJR069C	HAM1	0,825	0,895	0,922	14	17	14	18
YEL026W	SNU13	0,848	0,92	0,922	16	11	15	9
YGR253C	PUP2	0,812	0,881	0,922	19	15	15	14
YIL044C	AGE2	0,716	0,777	0,921	5	10	6	8
YHR163W	SOL3	0,845	0,917	0,921	18	19	16	21
YGL011C	SCL1	0,831	0,902	0,921	24	22	23	22
YPL137C	GIP3	0,784	0,851	0,921	10	8	7	8
YKR072C	SIS2	0,864	0,938	0,921	15	11	13	12
YPR180W	AOS1	0,869	0,944	0,921	2	7	1	9
YIR038C	GTT1	0,776	0,843	0,921	7	13	10	13
YJR121W	ATP2	0,878	0,954	0,920	112	73	115	70
YNR001C	CIT1	0,82	0,891	0,920	34	38	33	36
YIR006C	PAN1	0,785	0,853	0,920	41	52	44	47
YLL050C	COF1	0,831	0,903	0,920	23	21	23	25
YGL125W	MET13	0,473	0,514	0,920	19	22	24	22
YDR399W	HPT1	0,864	0,939	0,920	14	15	17	15
YOR234C	RPL33B	0,817	0,888	0,920	3	3	3	3
YJL179W	PFD1	0,667	0,725	0,920	9	5	9	7
YDR365C	ESF1	0,828	0,9	0,920	23	25	21	24
YLR106C	MDN1	0,829	0,902	0,919	29	18	25	15
YNL050C	YNL050C	0,863	0,939	0,919	6	7	4	8
YNL096C	RPS7B	0,804	0,875	0,919	43	32	42	32
YOR021C	YOR021C	0,866	0,943	0,918	24	28	21	25
YKL180W	RPL17A	0,853	0,929	0,918	121	69	121	63
YJR113C	RSM7	0,873	0,951	0,918	7	10	8	11
YER114C	BOI2	0,704	0,767	0,918	5	15	5	16
YML131W	YML131W	0,838	0,913	0,918	17	19	17	17
YNL167C	SKO1	0,636	0,693	0,918	4	2	4	2
YMR161W	HLJ1	0,717	0,782	0,917	2	2	1	2
YDL120W	YFH1	0,75	0,818	0,917	4	5	3	3
YGR282C	BGL2	0,397	0,433	0,917	11	12	13	13
YAR007C	RFA1	0,782	0,853	0,917	18	24	14	22
YGR008C	STF2	0,506	0,552	0,917	6	7	6	6
YGR157W	CHO2	0,78	0,851	0,917	9	12	10	12
YIL148W	RPL40A	0,834	0,91	0,916	19	14	20	15

YDR146C	SWI5	0,197	0,215	0,916	2	1	1	1
YFR015C	GSY1	0,777	0,848	0,916	3	2	3	3
YOL061W	PRS5	0,798	0,871	0,916	27	28	27	28
YDL148C	NOP14	0,85	0,928	0,916	20	10	22	9
YGR185C	TYS1	0,836	0,913	0,916	38	35	37	35
YJL186W	MNN5	0,738	0,806	0,916	17	12	19	16
YJR065C	ARP3	0,867	0,947	0,916	15	19	16	17
YGR128C	UTP8	0,758	0,828	0,915	10	16	13	16
YJL026W	RNR2	0,789	0,862	0,915	27	25	26	24
YOR323C	PRO2	0,81	0,885	0,915	27	34	29	31
YOR057W	SGT1	0,734	0,802	0,915	4	8	5	9
YLR337C	VRP1	0,83	0,907	0,915	13	14	18	11
YHR167W	THP2	0,819	0,895	0,915	3	4	2	4
YMR239C	RNT1	0,817	0,893	0,915	5	5	4	6
YHR113W	YHR113W	0,815	0,891	0,915	19	22	17	22
YDR294C	DPL1	0,761	0,832	0,915	11	8	10	8
YKR039W	GAP1	0,332	0,363	0,915	1	1	1	1
YNL306W	MRPS18	0,716	0,783	0,914	4	12	3	12
YER031C	YPT31	0,833	0,911	0,914	7	9	8	10
YMR038C	CCS1	0,744	0,814	0,914	25	21	24	21
YNL006W	LST8	0,754	0,825	0,914	2	2	1	2
YOL124C	TRM11	0,837	0,916	0,914	3	5	4	4
YDR257C	RKM4	0,793	0,868	0,914	4	5	3	4
YLR270W	DCS1	0,835	0,914	0,914	14	22	14	23
YMR008C	PLB1	0,904	0,99	0,913	1	3	1	3
YDR272W	GLO2	0,702	0,769	0,913	16	20	14	16
YOR165W	SEY1	0,848	0,929	0,913	14	16	14	15
YDR412W	RRP17	0,827	0,906	0,913	10	7	13	3
YKL127W	PGM1	0,847	0,928	0,913	13	14	14	15
YPL106C	SSE1	0,836	0,916	0,913	187	147	187	136
YDR266C	YDR266C	0,794	0,87	0,913	14	18	12	17
YKL120W	OAC1	0,803	0,88	0,913	16	15	16	13
YDR395W	SXM1	0,792	0,868	0,912	7	9	7	7
YOR212W	STE4	0,729	0,799	0,912	5	6	4	3
YLR015W	BRE2	0,874	0,958	0,912	3	4	3	5
YIR034C	LYS1	0,8	0,877	0,912	25	37	28	37
YFL021W	GAT1	0,322	0,353	0,912	1	1	1	1
YHR005C	GPA1	0,745	0,817	0,912	7	8	9	6
YJR051W	OSM1	0,827	0,907	0,912	2	4	2	4

YDR487C	RIB3	0,837	0,918	0,912	18	19	17	17
YJL074C	SMC3	0,785	0,861	0,912	19	25	18	25
YJL122W	ALB1	0,72	0,79	0,911	24	10	23	12
YER126C	NSA2	0,473	0,519	0,911	18	19	20	20
YML110C	COQ5	0,745	0,818	0,911	15	18	16	18
YGL122C	NAB2	0,816	0,896	0,911	8	14	6	14
YFL048C	EMP47	0,801	0,88	0,910	8	8	8	7
YDR019C	GCV1	0,78	0,857	0,910	17	24	16	24
YNL183C	NPR1	0,83	0,912	0,910	4	4	4	4
YGR135W	PRE9	0,808	0,888	0,910	14	16	16	15
YJL176C	SWI3	0,808	0,888	0,910	11	29	12	27
YDR341C	YDR341C	0,835	0,918	0,910	51	59	58	61
YBL045C	COR1	0,875	0,962	0,910	53	38	54	38
YMR149W	SWP1	0,853	0,938	0,909	11	6	10	7
YDL145C	COP1	0,782	0,86	0,909	36	39	40	39
YJL060W	BNA3	0,868	0,955	0,909	23	26	23	28
YNL251C	NRD1	0,697	0,767	0,909	7	15	7	14
YPL105C	SYH1	0,796	0,876	0,909	17	17	12	20
YBR208C	DUR1,2	0,783	0,862	0,908	26	15	22	13
YOR294W	RRS1	0,822	0,905	0,908	16	13	18	12
YDR378C	LSM6	0,868	0,956	0,908	9	6	6	6
YHR170W	NMD3	0,796	0,877	0,908	7	12	7	10
YMR246W	FAA4	0,648	0,714	0,908	20	18	18	16
YER151C	UBP3	0,814	0,897	0,907	23	30	20	31
YKL193C	SDS22	0,852	0,939	0,907	17	17	21	22
YHR138C	YHR138C	0,646	0,712	0,907	4	8	5	8
YGR175C	ERG1	0,205	0,226	0,907	4	8	8	8
YOL143C	RIB4	0,732	0,807	0,907	20	15	22	15
YOL021C	DIS3	0,808	0,891	0,907	19	19	19	21
YOR206W	NOC2	0,876	0,966	0,907	21	23	20	21
YIL145C	PAN6	0,837	0,923	0,907	9	10	9	11
YIL098C	FMC1	0,816	0,9	0,907	6	5	7	5
YEL071W	DLD3	0,851	0,939	0,906	69	60	71	61
YDR496C	PUF6	0,84	0,927	0,906	25	29	26	27
YBR164C	ARL1	0,809	0,893	0,906	4	6	4	5
YLR319C	BUD6	0,789	0,871	0,906	6	9	9	8
YIL095W	PRK1	0,721	0,796	0,906	7	6	7	6
YLR026C	SED5	0,816	0,901	0,906	5	9	8	8
YDL065C	PEX19	0,815	0,9	0,906	12	20	6	22

YOR042W	CUE5	0,805	0,889	0,906	14	14	15	17
YKL150W	MCR1	0,872	0,963	0,906	38	39	34	35
YLR330W	CHS5	0,755	0,834	0,905	12	22	13	24
YFR009W	GCN20	0,79	0,873	0,905	26	27	26	27
YDL156W	YDL156W	0,644	0,712	0,904	3	2	3	2
YJL198W	PHO90	0,587	0,649	0,904	3	2	3	2
YHR024C	MAS2	0,899	0,994	0,904	18	18	19	17
YNL233W	BNI4	0,681	0,753	0,904	9	15	8	15
YDR234W	LYS4	0,851	0,941	0,904	20	20	20	23
YJL168C	SET2	0,623	0,689	0,904	5	4	5	5
YDR372C	VPS74	0,755	0,835	0,904	18	19	15	20
YLR222C	UTP13	0,745	0,824	0,904	8	17	11	17
YOR136W	IDH2	0,761	0,842	0,904	36	34	34	33
YIL039W	TED1	0,805	0,891	0,903	2	4	3	5
YJL115W	ASF1	0,87	0,963	0,903	3	5	5	5
YOL010W	RCL1	0,821	0,909	0,903	3	7	1	7
YNL229C	URE2	0,755	0,836	0,903	4	6	4	6
YJL054W	TIM54	0,799	0,885	0,903	10	13	13	13
YHR097C	YHR097C	0,65	0,72	0,903	12	13	10	11
YOR184W	SER1	0,817	0,905	0,903	40	39	41	39
YJL042W	MHP1	0,797	0,883	0,903	10	6	8	7
YNL064C	YDJ1	0,834	0,924	0,903	45	49	45	48
YML011C	RAD33	0,667	0,739	0,903	1	2	1	2
YOR163W	DDP1	0,864	0,958	0,902	4	9	3	9
YCR054C	CTR86	0,835	0,926	0,902	2	2	2	2
YNL041C	COG6	0,734	0,814	0,902	3	6	2	7
YLR420W	URA4	0,723	0,802	0,901	16	32	19	32
YML124C	TUB3	0,704	0,781	0,901	7	8	7	9
YIL149C	MLP2	0,731	0,811	0,901	8	11	9	14
YKR048C	NAP1	0,765	0,849	0,901	27	31	28	30
YNL220W	ADE12	0,828	0,919	0,901	40	51	40	50
YOL039W	RPP2A	0,827	0,918	0,901	43	35	42	34
YDL182W	LYS20	0,831	0,923	0,900	40	40	39	38
YOR108W	LEU9	0,83	0,922	0,900	16	24	19	23
YEL037C	RAD23	0,766	0,851	0,900	7	5	6	7
YER086W	ILV1	0,806	0,896	0,900	41	52	44	50
YNR049C	MSO1	0,752	0,836	0,900	3	3	2	3
YKL053C-A	MDM35	0,832	0,925	0,899	3	6	5	6
YER008C	SEC3	0,662	0,736	0,899	4	2	5	2

YLR244C	MAP1	0,767	0,853	0,899	19	29	20	29
YDR513W	GRX2	0,829	0,922	0,899	18	20	18	18
YLL011W	SOF1	0,686	0,763	0,899	11	11	12	11
YLR002C	NOC3	0,827	0,92	0,899	5	4	5	4
YBL024W	NCL1	0,831	0,925	0,898	37	50	35	51
YOL076W	MDM20	0,742	0,826	0,898	8	3	5	2
YPR168W	NUT2	0,724	0,806	0,898	5	5	3	5
YGL233W	SEC15	0,66	0,735	0,898	2	1	3	1
YFL024C	EPL1	0,554	0,617	0,898	3	2	3	2
YJR014W	TMA22	0,808	0,9	0,898	11	12	11	13
YHR114W	BZZ1	0,834	0,929	0,898	12	14	12	14
YDL074C	BRE1	0,804	0,896	0,897	7	12	9	9
YBR122C	MRPL36	0,821	0,915	0,897	9	8	11	8
YML081C-A	ATP18	0,733	0,817	0,897	6	5	5	5
YDR389W	SAC7	0,731	0,815	0,897	5	3	5	3
YNL247W	YNL247W	0,852	0,95	0,897	41	56	41	51
YMR173W	DDR48	0,737	0,822	0,897	9	9	9	10
YPL204W	HRR25	0,64	0,714	0,896	4	4	4	6
YIL008W	URM1	0,835	0,932	0,896	2	1	3	1
YPR165W	RHO1	0,809	0,903	0,896	9	10	8	10
YHR018C	ARG4	0,813	0,908	0,895	36	29	28	29
YNL112W	DBP2	0,855	0,955	0,895	42	49	47	48
YCL012C	YCL012C	0,641	0,716	0,895	3	2	2	2
YBR111W-A	SUS1	0,564	0,63	0,895	3	4	2	6
YKL040C	NFU1	0,768	0,858	0,895	13	14	13	14
YLR045C	STU2	0,768	0,858	0,895	11	8	9	7
YOR048C	RAT1	0,844	0,943	0,895	8	8	7	10
YMR153W	NUP53	0,784	0,876	0,895	16	15	16	15
YBL103C	RTG3	0,612	0,684	0,895	3	6	2	6
YJL096W	MRPL49	0,87	0,973	0,894	10	9	8	9
YOR304C-A	YOR304C-A	0,657	0,735	0,894	4	5	3	5
YBR198C	TAF5	0,774	0,866	0,894	6	5	4	4
YKR056W	TRM2	0,755	0,845	0,893	19	26	22	25
YPL043W	NOP4	0,694	0,777	0,893	24	28	24	27
YOR252W	TMA16	0,627	0,702	0,893	6	9	6	9
YMR093W	UTP15	0,793	0,888	0,893	7	13	6	13
YLR369W	SSQ1	0,884	0,99	0,893	16	24	16	21
YNL098C	RAS2	0,8	0,896	0,893	26	20	29	19
YOR020C	HSP10	0,883	0,989	0,893	39	20	40	17

YKL212W	SAC1	0,779	0,873	0,892	18	16	17	10
YGR078C	PAC10	0,793	0,889	0,892	14	13	11	14
YMR227C	TAF7	0,793	0,889	0,892	8	11	7	7
YPL143W	RPL33A	0,825	0,925	0,892	39	37	43	38
YPL178W	CBC2	0,824	0,924	0,892	6	8	6	8
YEL046C	GLY1	0,749	0,84	0,892	30	42	29	40
YDR231C	COX20	0,762	0,855	0,891	8	4	8	3
YML106W	URA5	0,827	0,928	0,891	17	26	20	24
YER007C-A	TMA20	0,835	0,937	0,891	15	21	16	20
YLR196W	PWP1	0,81	0,909	0,891	20	37	22	41
YMR043W	MCM1	0,695	0,78	0,891	1	1	1	1
YBL079W	NUP170	0,85	0,954	0,891	13	7	10	7
YDL021W	GPM2	0,768	0,862	0,891	9	13	9	15
YFL047W	RGD2	0,733	0,823	0,891	7	9	5	7
YDR153C	ENT5	0,692	0,777	0,891	11	10	13	12
YLR433C	CNA1	0,822	0,923	0,891	2	3	4	3
YKL069W	YKL069W	0,854	0,959	0,891	5	11	6	11
YGL076C	RPL7A	0,813	0,913	0,890	89	60	89	60
YDL122W	UBP1	0,796	0,894	0,890	9	12	8	12
YGL232W	TAN1	0,851	0,956	0,890	4	10	5	10
YGL006W	PMC1	0,859	0,965	0,890	3	4	3	5
YBR214W	SDS24	0,623	0,7	0,890	4	7	5	7
YKL137W	CMC1	0,36	0,405	0,889	3	5	4	7
YKR067W	GPT2	0,839	0,944	0,889	7	6	6	6
YGL184C	STR3	0,783	0,881	0,889	10	13	10	14
YPR016C	TIF6	0,798	0,898	0,889	5	7	6	7
YML062C	MFT1	0,782	0,88	0,889	7	12	11	9
YOR285W	RDL1	0,781	0,879	0,889	16	16	14	16
YJL050W	MTR4	0,78	0,878	0,888	22	26	20	26
YPL134C	ODC1	0,818	0,921	0,888	1	2	1	2
YLR071C	RGR1	0,69	0,777	0,888	5	1	3	1
YOR321W	PMT3	0,871	0,981	0,888	2	2	3	2
YML055W	SPC2	0,852	0,96	0,888	5	2	5	1
YJL001W	PRE3	0,858	0,967	0,887	8	12	7	12
YML012W	ERV25	0,698	0,787	0,887	7	5	8	6
YDR427W	RPN9	0,835	0,942	0,886	15	25	20	25
YNL119W	NCS2	0,85	0,959	0,886	6	12	6	12
YLR413W	YLR413W	0,739	0,834	0,886	4	4	4	4
YBL056W	PTC3	0,791	0,893	0,886	5	6	5	6

YPR086W	SUA7	0,649	0,733	0,885	4	2	4	2
YGL223C	COG1	0,834	0,942	0,885	2	2	2	1
YBR130C	SHE3	0,809	0,914	0,885	11	13	12	12
YLR113W	HOG1	0,801	0,905	0,885	9	16	13	16
YPL212C	PUS1	0,807	0,912	0,885	34	38	33	36
YPL101W	ELP4	0,822	0,929	0,885	4	6	4	5
YHR147C	MRPL6	0,503	0,569	0,884	5	8	4	8
YOR051C	ETT1	0,746	0,844	0,884	14	9	11	12
YDL161W	ENT1	0,729	0,825	0,884	7	9	5	9
YMR016C	SOK2	0,667	0,755	0,883	4	11	5	13
YCL033C	MXR2	0,772	0,874	0,883	1	4	1	4
YOR046C	DBP5	0,764	0,865	0,883	30	33	31	37
YOR098C	NUP1	0,717	0,812	0,883	24	25	28	25
YHR052W	CIC1	0,821	0,93	0,883	21	34	24	36
YFL010C	WWM1	0,757	0,858	0,882	5	4	4	4
YOR123C	LEO1	0,793	0,899	0,882	6	13	8	13
YPR018W	RLF2	0,613	0,695	0,882	6	6	6	8
YOR022C	YOR022C	0,814	0,923	0,882	1	3	1	4
YFL008W	SMC1	0,746	0,846	0,882	6	9	10	9
YMR062C	ARG7	0,826	0,937	0,882	38	42	45	45
YGR013W	SNU71	0,818	0,928	0,881	2	5	2	5
YML097C	VPS9	0,757	0,859	0,881	5	6	4	7
YBR082C	UBC4	0,823	0,934	0,881	9	12	11	11
YDR138W	HPR1	0,709	0,805	0,881	2	3	2	4
YPR112C	MRD1	0,72	0,818	0,880	23	43	24	42
YNL166C	BNI5	0,638	0,725	0,880	12	19	14	19
YOL097C	WRS1	0,811	0,922	0,880	41	50	41	50
YKL016C	ATP7	0,721	0,82	0,879	35	25	30	22
YLR390W-A	CCW14	0,284	0,323	0,879	5	5	4	4
YLR038C	COX12	0,699	0,795	0,879	9	11	11	12
YDR227W	SIR4	0,734	0,835	0,879	1	5	1	4
YIR021W	MRS1	0,821	0,934	0,879	1	2	1	2
YGR277C	CAB4	0,835	0,95	0,879	2	5	2	4
YOR145C	PNO1	0,697	0,793	0,879	13	12	12	9
YHR029C	YHI9	0,853	0,971	0,878	10	13	10	14
YBR236C	ABD1	0,809	0,921	0,878	2	7	2	7
YKR028W	SAP190	0,683	0,778	0,878	11	9	13	9
YAR015W	ADE1	0,85	0,969	0,877	22	28	23	31
YAL019W	FUN30	0,699	0,797	0,877	9	7	11	7

YJR034W	PET191	0,875	0,998	0,877	4	7	3	5
YCR016W	YCR016W	0,832	0,949	0,877	10	16	7	16
YFR004W	RPN11	0,732	0,835	0,877	14	18	15	16
YBR023C	CHS3	0,71	0,81	0,877	3	4	3	2
YER127W	LCP5	0,823	0,939	0,876	7	9	8	8
YNL244C	SUI1	0,822	0,938	0,876	18	16	22	16
YPL126W	NAN1	0,806	0,92	0,876	17	25	16	24
YER168C	CCA1	0,741	0,846	0,876	3	8	5	9
YBR288C	APM3	0,804	0,918	0,876	1	1	1	1
YJL111W	CCT7	0,804	0,918	0,876	34	35	33	36
YGL169W	SUA5	0,739	0,844	0,876	1	7	1	7
YGR087C	PDC6	0,702	0,802	0,875	1	1	1	1
YLR017W	MEU1	0,812	0,928	0,875	6	16	9	14
YPL188W	POS5	0,791	0,904	0,875	5	9	5	10
YKL009W	MRT4	0,818	0,935	0,875	19	22	16	22
YJL039C	NUP192	0,752	0,86	0,874	8	5	9	4
YDL190C	UFD2	0,792	0,906	0,874	9	6	7	7
YNL075W	IMP4	0,791	0,905	0,874	8	11	7	9
YLL029W	FRA1	0,735	0,841	0,874	2	6	1	7
YPL078C	ATP4	0,754	0,864	0,873	26	24	30	24
YJL002C	OST1	0,724	0,83	0,872	8	10	9	10
YOL012C	HTZ1	0,743	0,852	0,872	3	3	4	4
YOR087W	YVC1	0,782	0,897	0,872	2	2	2	2
YHR158C	KEL1	0,761	0,873	0,872	19	34	22	32
YGR072W	UPF3	0,774	0,888	0,872	3	2	4	2
YLR364W	GRX8	0,825	0,947	0,871	6	9	7	9
YEL042W	GDA1	0,735	0,844	0,871	16	18	15	19
YGL153W	PEX14	0,745	0,856	0,870	8	9	8	8
YMR108W	ILV2	0,761	0,875	0,870	80	76	85	79
YKR082W	NUP133	0,78	0,897	0,870	15	19	18	17
YNL118C	DCP2	0,751	0,864	0,869	16	33	16	35
YGL057C	GEP7	0,723	0,832	0,869	1	4	2	3
YKL139W	CTK1	0,745	0,858	0,868	4	4	5	5
YEL040W	UTR2	0,572	0,659	0,868	9	6	8	7
YDL097C	RPN6	0,769	0,886	0,868	17	32	16	33
YML004C	GLO1	0,65	0,749	0,868	12	14	13	13
YDL225W	SHS1	0,792	0,913	0,867	20	32	22	31
YKL047W	YKL047W	0,764	0,881	0,867	7	5	6	4
YGR117C	YGR117C	0,626	0,722	0,867	5	6	4	7

YFL036W	RPO41	0,586	0,676	0,867	3	2	4	3
YDL192W	ARF1	0,832	0,96	0,867	6	12	8	12
YNR018W	AIM38	0,428	0,494	0,866	4	7	4	6
YPL211W	NIP7	0,849	0,98	0,866	12	19	12	16
YOL038W	PRE6	0,751	0,867	0,866	19	18	19	19
YKL079W	SMY1	0,781	0,902	0,866	2	3	2	3
YDR229W	IVY1	0,684	0,79	0,866	3	7	1	7
YMR236W	TAF9	0,729	0,842	0,866	2	5	4	3
YBR026C	ETR1	0,805	0,93	0,866	24	20	23	19
YKR066C	CCP1	0,862	0,996	0,865	16	30	14	30
YJR072C	NPA3	0,746	0,862	0,865	7	10	6	9
YGR145W	ENP2	0,712	0,823	0,865	15	18	19	21
YBR229C	ROT2	0,853	0,986	0,865	6	3	5	4
YMR128W	ECM16	0,813	0,94	0,865	13	10	11	11
YCR087C-A	YCR087C-A	0,819	0,947	0,865	7	6	7	6
YOR103C	OST2	0,799	0,924	0,865	3	3	3	3
YGR002C	SWC4	0,681	0,788	0,864	3	5	3	4
YEL038W	UTR4	0,821	0,95	0,864	7	14	6	12
YNL100W	AIM37	0,686	0,794	0,864	5	5	5	4
YGL073W	HSF1	0,78	0,903	0,864	8	9	7	9
YJR148W	BAT2	0,779	0,902	0,864	5	11	7	10
YHR025W	THR1	0,839	0,972	0,863	11	12	12	11
YDR258C	HSP78	0,856	0,992	0,863	28	59	32	55
YOL090W	MSH2	0,791	0,917	0,863	11	8	9	8
YKL178C	STE3	0,301	0,349	0,862	3	3	2	3
YML105C	SEC65	0,771	0,894	0,862	12	13	11	12
YER180C-A	SLO1	0,694	0,805	0,862	2	4	2	4
YDL098C	SNU23	0,768	0,891	0,862	4	5	7	5
YMR027W	YMR027W	0,805	0,934	0,862	13	16	15	16
YIL115C	NUP159	0,79	0,917	0,862	32	35	33	35
YNL051W	COG5	0,583	0,677	0,861	1	3	3	3
YGL112C	TAF6	0,676	0,785	0,861	2	4	2	5
YGR275W	RTT102	0,769	0,893	0,861	6	7	7	7
YAL059W	ECM1	0,749	0,87	0,861	16	11	17	10
YLR363W-A	YLR363W-A	0,58	0,674	0,861	7	4	6	4
YEL051W	VMA8	0,734	0,853	0,860	11	9	13	11
YNL035C	YNL035C	0,703	0,817	0,860	2	4	2	4
YHR216W	IMD2	0,775	0,901	0,860	11	15	10	15
YBR092C	PHO3	0,658	0,765	0,860	12	12	14	14

YHR012W	VPS29	0,786	0,914	0,860	2	6	4	6
YOL094C	RFC4	0,782	0,91	0,859	5	8	4	7
YHR089C	GAR1	0,817	0,951	0,859	13	15	11	14
YGL083W	SCY1	0,835	0,973	0,858	8	2	9	4
YDR167W	TAF10	0,691	0,806	0,857	6	8	7	8
YMR146C	TIF34	0,75	0,875	0,857	34	42	34	33
YGR210C	YGR210C	0,753	0,879	0,857	5	8	6	9
YER122C	GLO3	0,735	0,858	0,857	28	37	28	36
YOR006C	TSR3	0,794	0,927	0,857	4	3	3	3
YBR247C	ENP1	0,799	0,933	0,856	20	24	18	24
YBR109C	CMD1	0,785	0,917	0,856	8	12	10	13
YBR101C	FES1	0,654	0,764	0,856	11	16	9	17
YMR125W	STO1	0,729	0,852	0,856	15	10	17	13
YNL113W	RPC19	0,791	0,925	0,855	6	10	8	10
YGL004C	RPN14	0,749	0,876	0,855	3	3	3	4
YNR013C	PHO91	0,772	0,903	0,855	5	2	5	4
YPR190C	RPC82	0,742	0,868	0,855	9	12	8	11
YKL122C	SRP21	0,76	0,89	0,854	10	12	10	10
YBR095C	RXT2	0,687	0,805	0,853	6	6	7	6
YDL082W	RPL13A	0,779	0,914	0,852	2	3	3	4
YFR013W	IOC3	0,784	0,92	0,852	9	7	7	6
YOR176W	HEM15	0,824	0,967	0,852	12	18	11	19
YKL005C	BYE1	0,645	0,757	0,852	1	1	1	1
YML085C	TUB1	0,799	0,938	0,852	20	20	20	21
YCR009C	RVS161	0,776	0,911	0,852	12	13	14	12
YDR021W	FAL1	0,741	0,87	0,852	1	4	2	4
YJR135W-A	TIM8	0,837	0,983	0,851	13	6	10	5
YLR143W	YLR143W	0,683	0,803	0,851	4	8	6	9
YLL048C	YBT1	0,812	0,955	0,850	24	19	20	17
YBR057C	MUM2	0,498	0,586	0,850	1	4	1	5
YDL130W	RPP1B	0,718	0,845	0,850	13	8	14	9
YDL164C	CDC9	0,667	0,785	0,850	3	7	3	8
YOR287C	RRP36	0,672	0,791	0,850	4	4	7	4
YGR083C	GCD2	0,75	0,883	0,849	8	15	8	15
YPL218W	SAR1	0,755	0,889	0,849	7	9	6	8
YDR322C-A	TIM11	0,676	0,796	0,849	2	1	3	1
YCR026C	NPP1	0,623	0,734	0,849	1	1	1	1
YDL202W	MRPL11	0,78	0,919	0,849	6	13	4	13
YDR324C	UTP4	0,767	0,904	0,848	14	12	12	14

YHR094C	HXT1	0,479	0,565	0,848	3	2	3	2
YER049W	TPA1	0,773	0,912	0,848	27	33	31	34
YML079W	YML079W	0,816	0,963	0,847	7	10	8	10
YLR452C	SST2	0,603	0,712	0,847	9	7	9	7
YER148W	SPT15	0,557	0,658	0,847	7	9	8	10
YHR135C	YCK1	0,656	0,775	0,846	2	5	5	5
YER009W	NTF2	0,804	0,95	0,846	7	2	7	3
YLR248W	RCK2	0,773	0,914	0,846	14	14	15	12
YFL017C	GNA1	0,722	0,854	0,845	6	12	7	12
YHR112C	YHR112C	0,797	0,943	0,845	6	6	6	6
YDR362C	TFC6	0,757	0,896	0,845	2	3	2	3
YDL111C	RRP42	0,798	0,945	0,844	5	2	5	2
YGL050W	TYW3	0,727	0,861	0,844	4	5	4	4
YOL070C	NBA1	0,569	0,674	0,844	10	18	8	18
YNL103W	MET4	0,325	0,385	0,844	2	3	1	3
YNL271C	BNI1	0,777	0,922	0,843	8	3	7	3
YJR076C	CDC11	0,776	0,921	0,843	10	12	9	12
YOR039W	CKB2	0,763	0,906	0,842	4	10	5	10
YPR062W	FCY1	0,725	0,862	0,841	14	13	10	12
YMR033W	ARP9	0,808	0,961	0,841	9	12	10	12
YLR397C	AFG2	0,786	0,936	0,840	9	17	12	16
YML022W	APT1	0,775	0,923	0,840	9	9	11	9
YHL004W	MRP4	0,762	0,908	0,839	16	18	20	21
YDR394W	RPT3	0,746	0,889	0,839	17	25	19	19
YML034W	SRC1	0,777	0,926	0,839	6	4	6	3
YPL208W	RKM1	0,765	0,912	0,839	1	1	1	1
YML074C	FPR3	0,798	0,952	0,838	27	23	25	22
YHR056C	RSC30	0,709	0,846	0,838	3	2	2	2
YOR056C	NOB1	0,638	0,762	0,837	8	16	8	18
YLR450W	HMG2	0,715	0,854	0,837	1	1	1	2
YER019W	ISC1	0,509	0,608	0,837	3	1	3	1
YCR002C	CDC10	0,767	0,917	0,836	12	11	12	12
YBR215W	HPC2	0,693	0,829	0,836	4	5	6	6
YJR019C	TES1	0,735	0,88	0,835	2	2	2	2
YPR034W	ARP7	0,785	0,94	0,835	11	16	11	16
YFL046W	FMP32	0,747	0,895	0,835	5	5	4	4
YML036W	CGI121	0,781	0,936	0,834	2	5	2	4
YGL191W	COX13	0,428	0,513	0,834	3	9	5	11
YGR009C	SEC9	0,69	0,828	0,833	7	10	6	7

YGL190C	CDC55	0,695	0,835	0,832	5	14	6	14
YDR485C	VPS72	0,769	0,924	0,832	1	3	1	3
YPL265W	DIP5	0,489	0,588	0,832	3	3	2	1
YOL052C	SPE2	0,802	0,965	0,831	6	13	6	9
YPL210C	SRP72	0,814	0,98	0,831	14	21	13	20
YKR007W	MEH1	0,745	0,897	0,831	5	2	5	4
YHR060W	VMA22	0,73	0,879	0,830	2	5	1	5
YJL192C	SOP4	0,753	0,907	0,830	3	3	4	4
YDR264C	AKR1	0,693	0,835	0,830	3	2	5	3
YPR175W	DPB2	0,687	0,828	0,830	5	3	5	3
YGL203C	KEX1	0,812	0,979	0,829	8	9	9	11
YAL023C	PMT2	0,739	0,891	0,829	11	7	13	9
YGR208W	SER2	0,738	0,89	0,829	8	12	7	13
YOR109W	INP53	0,785	0,947	0,829	10	16	11	16
YMR260C	TIF11	0,76	0,918	0,828	14	16	9	16
YIL064W	SEE1	0,59	0,714	0,826	2	3	2	3
YJL010C	NOP9	0,733	0,889	0,825	6	13	6	14
YBR159W	IFA38	0,768	0,932	0,824	9	9	9	9
YIR024C	YIR024C	0,747	0,907	0,824	4	4	3	4
YAL044W-A	YAL044W-A	0,69	0,838	0,823	4	3	5	3
YNL310C	ZIM17	0,677	0,823	0,823	3	4	2	5
YDL132W	CDC53	0,702	0,854	0,822	10	11	9	9
YHR133C	NSG1	0,748	0,91	0,822	6	4	6	3
YOL080C	REX4	0,746	0,908	0,822	4	4	5	5
YCL029C	BIK1	0,74	0,901	0,821	4	9	5	8
YNR003C	RPC34	0,613	0,747	0,821	6	9	5	9
YBL054W	TOD6	0,512	0,624	0,821	10	15	11	13
YNL155W	YNL155W	0,392	0,478	0,820	3	4	5	3
YER027C	GAL83	0,815	0,995	0,819	1	2	1	2
YOR303W	CPA1	0,655	0,8	0,819	9	11	8	12
YBL101C	ECM21	0,79	0,967	0,817	3	4	2	3
YPL084W	BRO1	0,663	0,812	0,817	12	12	11	14
YJL101C	GSH1	0,667	0,817	0,816	15	15	16	16
YMR259C	YMR259C	0,675	0,827	0,816	4	1	3	1
YDR047W	HEM12	0,758	0,929	0,816	8	6	7	6
YGR055W	MUP1	0,59	0,725	0,814	11	8	13	11
YDR539W	FDC1	0,592	0,728	0,813	6	4	7	4
YDR161W	YDR161W	0,795	0,978	0,813	9	11	11	11
YKL032C	IXR1	0,558	0,687	0,812	12	13	10	12

YKR069W	MET1	0,727	0,896	0,811	19	26	14	26
YHR058C	MED6	0,628	0,774	0,811	1	3	2	4
YDR494W	RSM28	0,679	0,837	0,811	6	8	8	10
YKL068W	NUP100	0,771	0,951	0,811	13	21	13	19
YBR267W	REI1	0,612	0,755	0,811	14	19	11	16
YGR104C	SRB5	0,808	0,997	0,810	1	1	1	1
YDL076C	RXT3	0,73	0,902	0,809	4	7	3	6
YOL126C	MDH2	0,474	0,586	0,809	2	6	3	6
YMR099C	YMR099C	0,749	0,926	0,809	18	28	21	28
YOL016C	CMK2	0,791	0,979	0,808	3	2	4	2
YOR036W	PEP12	0,675	0,836	0,807	6	2	6	2
YNL032W	SIW14	0,665	0,824	0,807	2	5	1	5
YFR040W	SAP155	0,718	0,89	0,807	2	1	3	1
YIL131C	FKH1	0,546	0,677	0,806	3	3	3	3
YKR080W	MTD1	0,673	0,835	0,806	8	14	12	15
YPR135W	CTF4	0,759	0,943	0,805	8	6	6	5
YLR051C	FCF2	0,621	0,772	0,804	4	7	5	7
YDR201W	SPC19	0,651	0,811	0,803	2	4	2	4
YER017C	AFG3	0,667	0,831	0,803	12	8	12	8
YOR367W	SCP1	0,557	0,694	0,803	4	9	3	9
YDL167C	NRP1	0,711	0,886	0,802	4	3	5	3
YBR049C	REB1	0,719	0,897	0,802	15	23	16	25
YDR098C	GRX3	0,677	0,845	0,801	4	7	4	7
YPR091C	YPR091C	0,789	0,986	0,800	13	6	9	6
YNL256W	FOL1	0,753	0,943	0,799	7	8	7	9
YMR285C	NGL2	0,767	0,961	0,798	4	4	5	4
YOR222W	ODC2	0,755	0,947	0,797	4	8	6	8
YGL213C	SKI8	0,768	0,964	0,797	6	5	6	5
YGL197W	MDS3	0,603	0,757	0,797	2	1	3	1
YDR333C	YDR333C	0,677	0,85	0,796	1	1	2	1
YDR373W	FRQ1	0,673	0,845	0,796	1	2	1	2
YGR169C-A	YGR169C-A	0,714	0,897	0,796	6	6	4	5
YJL140W	RPB4	0,658	0,827	0,796	6	8	5	8
YBL016W	FUS3	0,505	0,635	0,795	2	4	2	5
YGL187C	COX4	0,668	0,84	0,795	12	17	12	16
YOR265W	RBL2	0,755	0,95	0,795	9	11	9	11
YML103C	NUP188	0,781	0,984	0,794	7	7	6	8
YLR399C	BDF1	0,644	0,812	0,793	10	23	14	21
YNL091W	NST1	0,701	0,884	0,793	7	7	7	6

YMR131C	RRB1	0,712	0,898	0,793	9	22	10	22
YNL272C	SEC2	0,667	0,843	0,791	7	8	5	8
YNL062C	GCD10	0,744	0,941	0,791	3	6	4	7
YNL129W	NRK1	0,706	0,894	0,790	7	11	7	10
YOR014W	RTS1	0,623	0,789	0,790	3	3	7	4
YLR074C	BUD20	0,441	0,559	0,789	7	9	5	9
YEL024W	RIP1	0,615	0,78	0,788	18	18	16	17
YKR089C	TGL4	0,492	0,624	0,788	3	3	5	2
YGR020C	VMA7	0,7	0,888	0,788	14	13	11	14
YGL040C	HEM2	0,754	0,957	0,788	15	21	12	23
YPR011C	YPR011C	0,733	0,932	0,786	1	2	1	2
YKL114C	APN1	0,769	0,978	0,786	4	7	5	6
YDR145W	TAF12	0,706	0,898	0,786	12	16	13	16
YMR097C	MTG1	0,395	0,503	0,785	1	3	1	3
YJR024C	MDE1	0,556	0,71	0,783	2	5	3	5
YNL197C	WHI3	0,738	0,943	0,783	5	8	5	6
YNL031C	HHT2	0,711	0,909	0,782	15	16	16	17
YGL066W	SGF73	0,563	0,72	0,782	4	5	5	4
YPL016W	SWI1	0,776	0,996	0,779	1	3	1	3
YPL207W	TYW1	0,531	0,682	0,779	10	12	11	8
YOL067C	RTG1	0,723	0,929	0,778	1	2	1	2
YMR216C	SKY1	0,599	0,771	0,777	3	10	5	7
YHL014C	YLF2	0,705	0,908	0,776	1	2	1	2
YLR188W	MDL1	0,329	0,426	0,772	2	2	2	2
YIL083C	CAB2	0,678	0,879	0,771	4	6	4	6
YBR166C	TYR1	0,743	0,965	0,770	5	7	8	5
YHL020C	OPI1	0,421	0,547	0,770	4	9	8	8
YNL027W	CRZ1	0,723	0,94	0,769	7	14	5	13
YDR163W	CWC15	0,338	0,44	0,768	1	3	1	3
YAL015C	NTG1	0,633	0,826	0,766	2	5	2	3
YHR108W	GGA2	0,733	0,957	0,766	19	18	21	19
YOR043W	WHI2	0,738	0,964	0,766	8	8	9	5
YDL223C	HBT1	0,646	0,844	0,765	10	13	10	13
YDR397C	NCB2	0,598	0,783	0,764	7	8	7	8
YOL032W	OPI10	0,655	0,858	0,763	5	5	4	4
YKR057W	RPS21A	0,637	0,836	0,762	3	3	4	4
YGR092W	DBF2	0,564	0,741	0,761	2	2	2	2
YBR073W	RDH54	0,379	0,498	0,761	1	2	1	3
YKR023W	YKR023W	0,696	0,915	0,761	9	7	7	7

YER134C	YER134C	0,684	0,904	0,757	7	12	9	12
YDL188C	PPH22	0,616	0,816	0,755	2	2	2	2
YBR030W	RKM3	0,682	0,904	0,754	2	1	1	2
YIR003W	AIM21	0,657	0,871	0,754	18	23	22	26
YIL138C	TPM2	0,666	0,883	0,754	10	14	12	15
YPL190C	NAB3	0,662	0,879	0,753	4	15	3	15
YER014W	HEM14	0,626	0,833	0,752	4	4	5	4
YLR407W	YLR407W	0,356	0,474	0,751	3	4	3	3
YHR083W	SAM35	0,675	0,899	0,751	3	3	4	3
YGR021W	YGR021W	0,745	0,993	0,750	3	4	3	4
YOR347C	PYK2	0,646	0,862	0,749	6	10	5	9
YML045W	YML045W	0,502	0,67	0,749	37	30	38	31
YER048W-A	ISD11	0,633	0,845	0,749	4	7	4	7
YNL281W	HCH1	0,652	0,871	0,749	16	19	17	18
YDL121C	YDL121C	0,538	0,719	0,748	3	2	2	2
YNR034W	SOL1	0,727	0,973	0,747	2	6	2	6
YPR058W	YMC1	0,458	0,613	0,747	1	2	1	3
YDL047W	SIT4	0,656	0,88	0,745	2	5	3	5
YJR057W	CDC8	0,706	0,949	0,744	1	4	1	4
YIL007C	NAS2	0,629	0,846	0,743	3	2	4	4
YBR140C	IRA1	0,697	0,938	0,743	2	1	2	2
YKL190W	CNB1	0,714	0,961	0,743	3	1	3	2
YDL207W	GLE1	0,736	0,992	0,742	4	2	4	2
YNL068C	FKH2	0,451	0,608	0,742	1	2	1	2
YJL201W	ECM25	0,693	0,935	0,741	2	2	2	1
YGR081C	SLX9	0,486	0,656	0,741	13	10	13	11
YKL135C	APL2	0,672	0,909	0,739	7	10	9	10
YGL110C	CUE3	0,683	0,926	0,738	3	2	4	2
YJL053W	PEP8	0,701	0,952	0,736	5	6	3	7
YBL035C	POL12	0,727	0,988	0,736	8	13	8	10
YPL191C	YPL191C	0,639	0,871	0,734	1	1	1	1
YNL265C	IST1	0,665	0,907	0,733	4	3	3	3
YHR028C	DAP2	0,648	0,885	0,732	3	7	3	7
YKL042W	SPC42	0,594	0,812	0,732	3	7	3	7
YEL003W	GIM4	0,68	0,93	0,731	3	7	3	7
YJL141C	YAK1	0,668	0,915	0,730	3	2	2	2
YDR061W	YDR061W	0,701	0,961	0,729	1	8	1	7
YKR024C	DBP7	0,683	0,937	0,729	5	11	8	11
YDR484W	VPS52	0,664	0,912	0,728	2	2	3	2

YGR138C	TPO2	0,38	0,522	0,728	1	1	1	1
YGL101W	YGL101W	0,595	0,82	0,726	6	11	9	11
YMR135C	GID8	0,679	0,937	0,725	3	4	3	4
YOR221C	MCT1	0,657	0,909	0,723	4	3	4	3
YGR028W	MSP1	0,552	0,764	0,723	2	2	2	2
YDR110W	FOB1	0,589	0,816	0,722	2	2	2	1
YNR037C	RSM19	0,543	0,753	0,721	9	11	7	11
YLR059C	REX2	0,62	0,861	0,720	6	6	4	6
YHR214C-B	YHR214C-B	0,562	0,781	0,720	1	2	1	2
YNR038W	DBP6	0,584	0,813	0,718	8	10	9	7
YDR262W	YDR262W	0,323	0,45	0,718	4	10	2	11
YHL007C	STE20	0,649	0,905	0,717	8	18	8	19
YLR119W	SRN2	0,543	0,76	0,714	2	3	2	6
YMR244C-A	YMR244C-A	0,659	0,923	0,714	4	5	4	5
YGR260W	TNA1	0,549	0,77	0,713	5	7	6	7
YKL195W	MIA40	0,679	0,957	0,710	12	15	10	17
YBL042C	FUI1	0,412	0,581	0,709	5	2	4	4
YML017W	PSP2	0,626	0,884	0,708	9	16	10	14
YGL017W	ATE1	0,523	0,739	0,708	1	2	2	1
YOL051W	GAL11	0,44	0,623	0,706	6	6	4	6
YML067C	ERV41	0,593	0,841	0,705	2	3	2	3
YJR161C	COS5	0,422	0,6	0,703	1	1	1	1
YBR233W	PBP2	0,626	0,892	0,702	2	3	2	3
YFL044C	OTU1	0,614	0,876	0,701	1	1	1	1
YEL043W	YEL043W	0,631	0,905	0,697	1	2	1	2
YIL112W	HOS4	0,669	0,967	0,692	4	4	4	4
YOL030W	GAS5	0,574	0,83	0,692	3	3	4	3
YBR068C	BAP2	0,298	0,431	0,691	3	1	3	1
YCR069W	CPR4	0,286	0,414	0,691	1	2	2	2
YGL181W	GTS1	0,536	0,781	0,686	4	5	2	4
YKR074W	AIM29	0,638	0,93	0,686	2	11	5	11
YPR169W	JIP5	0,533	0,777	0,686	4	8	4	9
YHL024W	RIM4	0,369	0,541	0,682	1	1	1	1
YDL150W	RPC53	0,523	0,768	0,681	11	12	12	11
YGL117W	YGL117W	0,562	0,828	0,679	3	4	2	4
YER103W	SSA4	0,557	0,821	0,678	6	9	9	11
YDR041W	RSM10	0,656	0,969	0,677	3	3	5	3
YFR025C	HIS2	0,645	0,953	0,677	3	3	3	3
YNL133C	FYV6	0,32	0,473	0,677	2	3	2	4

YNL212W	VID27	0,575	0,852	0,675	7	12	11	13
YMR171C	EAR1	0,465	0,691	0,673	2	1	1	2
YLR033W	RSC58	0,654	0,974	0,671	5	11	5	10
YKL184W	SPE1	0,472	0,704	0,670	4	9	5	10
YLR086W	SMC4	0,646	0,966	0,669	3	2	3	2
YMR215W	GAS3	0,345	0,516	0,669	10	12	12	14
YEL066W	HPA3	0,639	0,956	0,668	3	6	5	5
YOR075W	UFE1	0,658	0,988	0,666	1	2	1	2
YDR038C	ENA5	0,498	0,748	0,666	5	8	8	8
YLR300W	EXG1	0,381	0,573	0,665	7	10	10	11
YDL102W	POL3	0,554	0,836	0,663	4	3	5	5
YGR250C	YGR250C	0,428	0,648	0,660	5	13	5	13
YGR252W	GCN5	0,474	0,72	0,658	4	7	4	10
YFL007W	BLM10	0,633	0,964	0,657	5	1	5	1
YNL262W	POL2	0,644	0,984	0,654	4	2	4	1
YIL038C	NOT3	0,534	0,818	0,653	5	15	7	16
YOR045W	TOM6	0,616	0,945	0,652	1	3	2	3
YDR059C	UBC5	0,496	0,765	0,648	3	3	4	3
YMR037C	MSN2	0,551	0,85	0,648	6	9	4	8
YNL110C	NOP15	0,628	0,971	0,647	7	9	7	8
YBR135W	CKS1	0,446	0,69	0,646	1	2	1	2
YGL005C	COG7	0,455	0,706	0,644	4	7	3	9
YLR052W	IES3	0,579	0,901	0,643	2	7	4	6
YLR006C	SSK1	0,463	0,724	0,640	1	3	2	3
YJR097W	JJJ3	0,477	0,748	0,638	1	2	2	2
YIL019W	FAF1	0,54	0,847	0,638	7	3	8	3
YBR007C	DSF2	0,432	0,682	0,633	2	3	2	5
YBL055C	YBL055C	0,594	0,939	0,633	3	8	2	8
YMR021C	MAC1	0,387	0,612	0,632	2	1	2	1
YFR047C	BNA6	0,55	0,87	0,632	2	6	1	7
YOL004W	SIN3	0,476	0,755	0,630	10	8	6	8
YBR200W	BEM1	0,535	0,849	0,630	4	11	4	11
YKL022C	CDC16	0,503	0,804	0,626	2	2	2	2
YMR098C	ATP25	0,476	0,761	0,625	3	1	4	1
YGL013C	PDR1	0,612	0,989	0,619	2	1	2	2
YDL067C	COX9	0,449	0,73	0,615	4	3	4	4
YBR042C	CST26	0,499	0,816	0,612	2	1	2	1
YPL107W	YPL107W	0,379	0,622	0,609	2	6	3	5
YGL094C	PAN2	0,575	0,952	0,604	1	1	1	1

YLR063W	YLR063W	0,566	0,938	0,603	2	3	2	3
YGR255C	COQ6	0,451	0,751	0,601	3	5	3	5
YCR057C	PWP2	0,558	0,934	0,597	4	8	4	7
YHR069C	RRP4	0,544	0,911	0,597	5	8	8	7
YDR349C	YPS7	0,578	0,968	0,597	1	1	1	1
YDL141W	BPL1	0,588	0,985	0,597	4	7	6	8
YBR197C	YBR197C	0,386	0,647	0,597	2	3	2	2
YBL068W	PRS4	0,563	0,95	0,593	7	9	7	8
YNR040W	YNR040W	0,541	0,915	0,591	3	3	4	4
YNL258C	DSL1	0,326	0,552	0,591	3	2	3	2
YHR030C	SLT2	0,58	0,983	0,590	5	8	3	6
YDR531W	CAB1	0,552	0,936	0,590	3	3	2	2
YER056C	FCY2	0,368	0,627	0,587	4	5	4	4
YDR246W	TRS23	0,482	0,824	0,585	2	4	5	5
YGL246C	RAI1	0,565	0,967	0,584	2	7	2	7
YIL135C	VHS2	0,465	0,798	0,583	3	1	3	1
YHR090C	YNG2	0,28	0,482	0,581	1	1	1	2
YLR092W	SUL2	0,39	0,674	0,579	15	14	19	17
YOL082W	ATG19	0,404	0,699	0,578	2	1	3	1
YJR053W	BFA1	0,471	0,818	0,576	2	1	2	1
YKL058W	TOA2	0,506	0,88	0,575	2	2	1	2
YKR090W	PXL1	0,435	0,762	0,571	1	3	2	2
YDL157C	YDL157C	0,404	0,711	0,568	5	3	2	2
YPL183C	RTT10	0,43	0,762	0,564	5	3	6	4
YNL260C	YNL260C	0,444	0,79	0,562	1	5	2	4
YBR212W	NGR1	0,39	0,701	0,556	3	5	4	5
YBR275C	RIF1	0,539	0,971	0,555	3	2	1	2
YOL069W	NUF2	0,386	0,709	0,544	1	3	1	4
YFR034C	PHO4	0,276	0,507	0,544	2	3	4	2
YOR085W	OST3	0,449	0,828	0,542	3	1	4	1
YKL063C	YKL063C	0,536	0,99	0,541	3	2	2	2
YJR091C	JSN1	0,392	0,729	0,538	2	1	2	1
YBR061C	TRM7	0,47	0,879	0,535	3	4	3	4
YJR142W	YJR142W	0,496	0,929	0,534	1	3	2	4
YIL140W	AXL2	0,417	0,785	0,531	5	3	5	3
YER040W	GLN3	0,496	0,959	0,517	1	1	1	1
YJR066W	TOR1	0,499	0,971	0,514	2	2	2	2
YDL219W	DTD1	0,453	0,893	0,507	4	8	4	8
YFR041C	ERJ5	0,363	0,725	0,501	3	3	3	4

YDL010W	GRX6	0,426	0,852	0,500	3	3	4	3
YMR291W	YMR291W	0,286	0,572	0,500	3	5	3	4
YOR037W	CYC2	0,47	0,941	0,499	1	1	2	2
YMR002W	MIC17	0,408	0,82	0,498	6	4	6	3
YBL005W-B	YBL005W-B	0,337	0,686	0,491	1	5	2	6
YGL084C	GUP1	0,447	0,912	0,490	1	1	2	1
YJL011C	RPC17	0,477	0,982	0,486	3	10	3	10
YER016W	BIM1	0,394	0,818	0,482	1	6	3	7
YMR313C	TGL3	0,34	0,706	0,482	1	1	2	1
YAL007C	ERP2	0,439	0,924	0,475	2	4	2	4
YMR059W	SEN15	0,205	0,437	0,469	1	2	2	2
YNR015W	SMM1	0,389	0,832	0,468	3	8	3	7
YLR093C	NYV1	0,371	0,797	0,465	2	1	2	1
YCR034W	FEN1	0,409	0,887	0,461	3	3	4	3
YOR017W	PET127	0,323	0,704	0,459	3	3	3	3
YMR113W	FOL3	0,404	0,886	0,456	2	3	1	3
YHR148W	IMP3	0,322	0,718	0,448	3	3	3	4
YIR005W	IST3	0,342	0,765	0,447	1	1	2	1
YLR395C	COX8	0,227	0,516	0,440	1	2	2	2
YAR014C	BUD14	0,247	0,581	0,425	2	3	1	2
YMR278W	PGM3	0,339	0,809	0,419	9	20	6	19
YIL157C	COA1	0,264	0,66	0,400	1	1	2	1
YBR038W	CHS2	0,329	0,841	0,391	1	1	2	1
YJL097W	PHS1	0,374	0,958	0,390	1	1	2	2
YDR510W	SMT3	0,318	0,821	0,387	8	12	9	12
YBL006C	LDB7	0,281	0,733	0,383	2	2	2	2
YNL090W	RHO2	0,368	0,972	0,379	2	1	2	1
YGL108C	YGL108C	0,313	0,829	0,378	2	2	1	2
YKL041W	VPS24	0,327	0,874	0,374	3	9	4	8
YPR067W	ISA2	0,325	0,876	0,371	2	2	1	2
YOL122C	SMF1	0,319	0,861	0,370	1	1	1	1
YMR121C	RPL15B	0,331	0,906	0,365	1	1	2	1
YGR129W	SYF2	0,244	0,7	0,349	4	7	1	6
YNR048W	YNR048W	0,296	0,85	0,348	1	1	1	1
YPL065W	VPS28	0,225	0,653	0,345	1	3	3	3
YML121W	GTR1	0,292	0,883	0,331	1	2	2	2
YLR120C	YPS1	0,303	0,932	0,325	4	3	5	3
YOL093W	TRM10	0,238	0,74	0,322	1	5	2	5
YIL114C	POR2	0,249	0,893	0,279	2	2	2	4

YJR026W	YJR026W	0,194	0,706	0,275	1	1	1	1
YDR092W	UBC13	0,202	0,772	0,262	1	2	2	2
YDR308C	SRB7	0,197	0,773	0,255	2	4	2	4
YBR087W	RFC5	0,221	0,895	0,247	3	5	2	5
YDR245W	MNN10	0,177	0,741	0,239	2	2	2	3
YMR289W	ABZ2	0,206	0,902	0,228	1	1	2	1
YPL019C	VTC3	0,223	0,982	0,227	2	2	2	2
YGL054C	ERV14	0,188	0,913	0,206	1	1	2	1
YPL214C	THI6	0,169	0,829	0,204	1	1	2	2
YPL007C	TFC8	0,162	0,819	0,198	1	1	2	1
YNL036W	NCE103	0,061	0,31	0,197	3	11	2	11
YGR177C	ATF2	0,114	0,709	0,161	1	2	2	2
YLR275W	SMD2	0,138	0,91	0,152	4	5	2	5
YDL128W	VCX1	0,081	0,536	0,151	1	2	2	2
YOR220W	RCN2	0,095	0,717	0,132	1	4	2	5
YDR200C	VPS64	0,074	0,712	0,104	2	3	1	3
YJR130C	STR2	0,093	0,963	0,097	3	5	3	5
YFL034C-A	RPL22B	0,062	0,824	0,075	4	7	6	7
YOL007C	CSI2	0,01	0,298	0,034	1	2	0	2
YPL038W	MET31	0,01	0,441	0,023	1	1	0	1
YLL012W	YEH1	0,01	0,494	0,020	1	1	0	1
YGR238C	KEL2	0,01	0,539	0,019	1	1	0	2
YPR184W	GDB1	0,01	0,54	0,019	1	2	0	3
YOR189W	IES4	0,01	0,545	0,018	2	3	0	3
YIL156W-B	YIL156W-B	0,01	0,581	0,017	1	1	0	1
YPL070W	MUK1	0,01	0,597	0,017	1	2	0	2
YMR052W	FAR3	0,01	0,609	0,016	1	1	0	1
YJL133W	MRS3	0,01	0,62	0,016	1	1	0	1
YPL124W	SPC29	0,01	0,676	0,015	1	2	0	2
YFR036W	CDC26	0,01	0,678	0,015	1	2	0	2
YMR288W	HSH155	0,01	0,691	0,014	1	1	0	2
YDR357C	CNL1	0,01	0,698	0,014	1	1	0	1
YDL043C	PRP11	0,01	0,7	0,014	1	3	0	3
YDR339C	FCF1	0,01	0,732	0,014	1	4	0	4
YGR007W	ECT1	0,01	0,733	0,014	1	2	0	3
YMR022W	UBC7	0,01	0,739	0,014	2	4	0	3
YLR363C	NMD4	0,01	0,758	0,013	1	3	0	3
YIL061C	SNP1	0,01	0,761	0,013	1	1	0	1
YDR480W	DIG2	0,01	0,766	0,013	1	2	0	2

YDL042C	SIR2	0,01	0,785	0,013	1	5	0	3
YBR175W	SWD3	0,01	0,786	0,013	1	1	0	1
YNL083W	SAL1	0,01	0,798	0,013	1	1	0	1
YJL030W	MAD2	0,01	0,819	0,012	1	1	0	1
YCR015C	YCR015C	0,01	0,829	0,012	1	1	0	1
YPL046C	ELC1	0,01	0,84	0,012	2	2	0	2
YLR361C-A	YLR361C-A	0,01	0,844	0,012	1	2	0	2
YHR003C	YHR003C	0,01	0,845	0,012	1	4	0	4
YOR173W	DCS2	0,01	0,85	0,012	2	1	0	1
YGL156W	AMS1	0,01	0,851	0,012	1	1	0	1
YHR144C	DCD1	0,01	0,853	0,012	1	2	0	2
YGR158C	MTR3	0,01	0,86	0,012	1	5	0	5
YDL006W	PTC1	0,01	0,862	0,012	1	1	0	1
YLR078C	BOS1	0,01	0,864	0,012	1	1	0	1
YGR047C	TFC4	0,01	0,871	0,011	1	1	0	1
YGL164C	YRB30	0,01	0,887	0,011	2	4	0	4
YPL064C	CWC27	0,01	0,901	0,011	1	1	0	1
YPL170W	DAP1	0,01	0,908	0,011	1	5	0	5
YPR179C	HDA3	0,01	0,91	0,011	1	5	0	5
YER071C	YER071C	0,01	0,947	0,011	3	3	0	4
YDR416W	SYF1	0,01	0,961	0,010	1	1	0	1
YDR013W	PSF1	0,01	0,978	0,010	1	4	0	3

Appendix Table S5 - related to Figure EV2:

Gene Ontology Analysis of Cellular Processes and Cellular Components from 76 proteins with reduced turnover in Dsc mutants.

GO terms with less than 2 genes in the dataset were not reported.

GOID	GO term	Adjusted p-value (FDR)	Enrichment	Frequency	Genome Frequency	
GO:0006629	lipid metabolic process	**0,099755517**	2,57	9 out of 76 genes, 11.8%	296 of 6433 genes, 4.6%	NUS1,MSS4,LCB3,YEH2,ERG3,ORM2,FLC1,PIS1,ANT1
GO:0055085	transmembrane transport	0,109828283	2,53	7 out of 76 genes, 9.2%	234 of 6433 genes, 3.6%	PHO87,FTR1,MPC1,PEX4,SAM37,AQR1,PEX25
GO:0061024	membrane organization	0,109828283	2,40	8 out of 76 genes, 10.5%	282 of 6433 genes, 4.4%	DRS2,DNF2,UGO1,YPP1,NEO1,SAM37,DNF3,VPS16
GO:0006399	tRNA metabolic process	0,123021299	2,33	5 out of 76 genes, 6.6%	182 of 6433 genes, 2.8%	QRI7,MSM1,AIR1,RPM2,MOD5
GO:0007005	mitochondrion organization	0,109828283	2,24	7 out of 76 genes, 9.2%	265 of 6433 genes, 4.1%	OM14,QRI7,UGO1,COA3,RPM2,SAM37,BUL1
GO:0006605	protein targeting	0,109828283	2,21	8 out of 76 genes, 10.5%	307 of 6433 genes, 4.8%	OM14,PEX4,YPP1,POM34,SPC3,SAM37,VPS16,PEX25
GO:0016192	vesicle-mediated transport	0,123021299	1,93	8 out of 76 genes, 10.5%	351 of 6433 genes, 5.5%	DRS2,DNF2,YPP1,NEO1,TVP18,ERP4,SLY41,VPS16
GO:0007010	cytoskeleton organization	0,204495159	1,78	5 out of 76 genes, 6.6%	238 of 6433 genes, 3.7%	AIM14,SPC97,NDC80,POM34,SHE4
GO:0007165	signal transduction	0,204495159	1,76	5 out of 76 genes, 6.6%	240 of 6433 genes, 3.7%	STE50,LRG1,KSP1,LCB3,IRA2
GO:0022618	ribonucleoprotein complex assembly	0,216699419	1,66	3 out of 76 genes, 3.9%	153 of 6433 genes, 2.4%	DRS2,PRP9,PRP8
GO:0030154	cell differentiation	0,22065489	1,59	3 out of 76 genes, 3.9%	160 of 6433 genes, 2.5%	MSS4,SHE4,SPO24
GO:0042592	homeostatic process	0,210336116	1,53	5 out of 76 genes, 6.6%	276 of 6433 genes, 4.3%	FTR1,YRF1-3,HMX1,ORM2,VHS3
GO:0006461	protein complex assembly	0,210336116	1,50	5 out of 76 genes, 6.6%	282 of 6433 genes, 4.4%	SPC97,NDC80,COA3,SAM37,VPS16
GO:0006397	mRNA processing	0,227818146	1,49	3 out of 76 genes, 3.9%	170 of 6433 genes, 2.6%	PRP9,PRP8,RPM2

GO:0051186	cofactor metabolic process	0,231789595	1,42	3 out of 76 genes, 3.9%	179 of 6433 genes, 2.8%	HMX1,VHS3,HEM4
GO:0071554	cell wall organization or biogenesis	0,24345729	1,28	3 out of 76 genes, 3.9%	198 of 6433 genes, 3.1%	LRG1,YEH2,FLC1
GO:0006464	cellular protein modification process	0,204495159	1,27	10 out of 76 genes, 13.2%	665 of 6433 genes, 10.3%	PSY4,STE50,NUS1,GCN2,PEX4,KSP1,UBP7,HIR3,BUL1,ALG5
GO:0000003	reproduction	0,210336116	1,27	7 out of 76 genes, 9.2%	467 of 6433 genes, 7.3%	DRS2,STE50,LRG1,DNF2,MSS4,DNF3,SHE4
GO:0048646	anatomical structure formation involved in morphogenesis	0,275924488	1,24	2 out of 76 genes, 2.6%	137 of 6433 genes, 2.1%	MSS4,SPO24
GO:0034655	nucleobase-containing compound catabolic process	0,275924488	1,16	2 out of 76 genes, 2.6%	146 of 6433 genes, 2.3%	AIR1,POL32
GO:0006950	response to stress	0,210336116	1,13	9 out of 76 genes, 11.8%	672 of 6433 genes, 10.4%	PSY4,STE50,HTL1,GCN2,RTT107,POL32,HMX1,ORM2,IRA2
GO:0048856	anatomical structure development	0,275924488	1,06	2 out of 76 genes, 2.6%	160 of 6433 genes, 2.5%	MSS4,SPO24
GO:0040007	growth	0,275924488	1,03	2 out of 76 genes, 2.6%	165 of 6433 genes, 2.6%	STE50,KSP1
GO:0007034	vacuolar transport	0,275924488	1,01	2 out of 76 genes, 2.6%	168 of 6433 genes, 2.6%	YPP1,VPS16
GO:0006351	transcription, DNA-templated	0,211197146	1,01	8 out of 76 genes, 10.5%	673 of 6433 genes, 10.5%	ROX3,HTL1,SAS4,HIR3,TOF2,RPM2,SIN4,MOD5
GO:0051276	chromosome organization	0,216699419	0,92	6 out of 76 genes, 7.9%	554 of 6433 genes, 8.6%	HTL1,SAS4,YRF1-3,HIR3,TOF2,MOD5
GO:0044281	small molecule metabolic process	0,211197146	0,88	7 out of 76 genes, 9.2%	675 of 6433 genes, 10.5%	PHO87,NUS1,MSM1,ERG3,IRA2,VHS3,ANT1
GO:00	cell cycle	0,211197146	0,79	6 out of 76	644 of 6433	PSY4,MSS4,GCN2,RTT107,HIR3,POM34

07049				genes, 7.9%	genes, 10.0%	
GO:0006259	DNA metabolic process	0,210336116	0,56	3 out of 76 genes, 3.9%	453 of 6433 genes, 7.0%	YRF1-3,RTT107,POL32
GO:0006412	translation	0,123021299	0,47	4 out of 76 genes, 5.3%	727 of 6433 genes, 11.3%	GCN2,MSM1,COA3,RPM2

**GO term set: generic GO-slim: Components**						
**GOID**	**GO term**	**Adjusted p-value (FDR)**	**Enrichment**	**Frequency**	**Genome Frequency**	
GO:0005777	peroxisome	0,158347118	3,34	3 out of 76 genes, 3.9%	76 of 6433 genes, 1.2%	PEX4,PEX25,ANT1
GO:0005886	plasma membrane	**0,016735143**	2,57	12 out of 76 genes, 15.8%	395 of 6433 genes, 6.1%	PHO87,YDL012C,DNF2,MSS4,FTR1,YPP1,YOR1,YEH2,DNF3,BUL1,AQR1,MSB1
GO:0005783	endoplasmic reticulum	**0,010121824**	2,34	17 out of 76 genes, 22.4%	615 of 6433 genes, 9.6%	DRS2,NUS1,DNF2,YSP2,AIM14,LCB3,ERG3,SPC3,HMX1,ORM2,DNF3,IRA2,ERP4,SLY41,FLC1,ALG5,PIS1
GO:0005794	Golgi apparatus	0,160654653	2,13	5 out of 76 genes, 6.6%	199 of 6433 genes, 3.1%	DRS2,NEO1,TVP18,DNF3,PIS1
GO:0005635	nuclear envelope	0,184478129	2,06	3 out of 76 genes, 3.9%	123 of 6433 genes, 1.9%	NUS1,POM34,HMX1
GO:0005768	endosome	0,200889434	1,78	3 out of 76 genes, 3.9%	143 of 6433 genes, 2.2%	YPP1,NEO1,VPS16
GO:0005730	nucleolus	0,200889434	1,29	5 out of 76 genes, 6.6%	329 of 6433 genes, 5.1%	CGR1,YGR283C,AIR1,TOF2,MOD5
GO:0005739	mitochondrion	0,184478129	1,15	16 out of 76 genes, 21.1%	1178 of 6433 genes, 18.3%	OM14,QRI7,LRG1,YSP2,UGO1,MPC1,MSM1,COA3,TOF2,YLR419W,RPM2,SAM37,IRA2,MSB1,MOD5,PIS1
GO:0043234	protein complex	0,184478129	1,11	15 out of 76 genes, 19.7%	1147 of 6433 genes, 17.8%	DRS2,ROX3,HTL1,SAS4,FTR1,SPC97,AIR1,NDC80,HIR3,POM34,SPC3,SAM37,SIN4,VHS3,VPS16
GO:0005623	cell	0,057243774	1,09	73 out of 76 genes, 96.1%	5680 of 6433 genes, 88.3%	DRS2,PSY4,ROX3,OM14,YPT10,STE50,HTL1,PHO87,YDL012C,PRP9,QRI7,NUS1,LRG1,DNF2,SAS4,MSS4,GCN2,YSP2,YDR415C,UGO1,FTR1,CGR1,MPC1,AIM14,PEX4,MSM1,YPP1,YOR1,YGR283C,YRF1-3, KSP1, RTT107, PRP8, SPC97, NEO1,AIR1,NDC80,UBP7,COA3,LCB3,POL32,HIR3,TO

						F2,POM34,YEH2,ERG3,SPC3,HMX1,ORM2,YLR419W,RPM2,SAM37,TVP18,DNF3,BUL1,AQR1,YNL234W,SIN4,IRA2,ERP4,SHE4,VHS3,CMR2,MSB1,MOD5,SLY41,VPS16,PEX25,FLC1,ALG5,SPO24,PIS1,ANT1
GO:0005773	vacuole	0,200889434	1,08	6 out of 76 genes, 7.9%	470 of 6433 genes, 7.3%	YPT10,YDR415C,AQR1,YNL234W,VPS16,FLC1
GO:0005622	intracellular	0,184478129	1,03	67 out of 76 genes, 88.2%	5511 of 6433 genes, 85.7%	DRS2,PSY4,ROX3,OM14,YPT10,STE50,HTL1,PRP9,QRI7,NUS1,LRG1,DNF2,SAS4,MSS4,GCN2,YSP2,YDR415C,UGO1,CGR1,MPC1,AIM14,PEX4,MSM1,YPP1,YGR283C,YRF1-3 ,KSP1, RTT107, PRP8, SPC97, NEO1, AIR1, NDC80,UBP7,COA3,LCB3,POL32,HIR3,TOF2,POM34,ERG3,SPC3,HMX1,ORM2,YLR419W,RPM2,SAM37,TVP18,DNF3,BUL1,AQR1,YNL234W,SIN4,IRA2,ERP4,SHE4,VHS3,CMR2,MSB1,MOD5,SLY41,VPS16,PEX25,FLC1,ALG5,PIS1,ANT1
GO:0005737	cytoplasm	0,184478129	1,00	51 out of 76 genes, 67.1%	4309 of 6433 genes, 67%	DRS2,PSY4,OM14,YPT10,STE50,QRI7,NUS1,LRG1,DNF2,GCN2,YSP2,YDR415C,UGO1,MPC1,AIM14,PEX4,MSM1,YPP1,YGR283C,KSP1,SPC97,NEO1,UBP7,COA3,LCB3,TOF2,ERG3,SPC3,HMX1,ORM2,YLR419W,RPM2,SAM37,TVP18,DNF3,AQR1,YNL234W,IRA2,ERP4,SHE4,VHS3,CMR2,MSB1,MOD5,SLY41,VPS16,PEX25,FLC1,ALG5,PIS1,ANT1
GO:0005654	nucleoplasm	0,239882312	0,97	3 out of 76 genes, 3.9%	263 of 6433 genes, 4.1%	ROX3,SAS4,SIN4
GO:0005840	ribosome	0,230706962	0,95	4 out of 76 genes, 5.3%	357 of 6433 genes, 5.5%	GCN2,AIM14,YPP1,YGR283C
GO:0005634	nucleus	0,184478129	0,92	24 out of 76 genes, 31.6%	2208 of 6433 genes, 34.3%	PSY4,ROX3,HTL1,PRP9,NUS1,SAS4,MSS4,CGR1,YPP1,YGR283C,YRF1-3, KSP1, RTT107, PRP8, AIR1, NDC80, POL32, HIR3,TOF2,POM34,HMX1,RPM2,SIN4,MOD5
GO:0005856	cytoskeleton	0,267366207	0,85	2 out of 76 genes, 2.6%	200 of 6433 genes, 3.1%	YPP1,SPC97
GO:0000228	nuclear chromosome	0,238726802	0,84	3 out of 76 genes, 3.9%	303 of 6433 genes, 4.7%	SAS4,NDC80,POL32
GO:00	cytosol	0,055746828	0,42	5 out of 76	1005 of 6433	GCN2,YPP1,IRA2,CMR2,MOD5

05829				genes, 6.6%	genes, 15.6%	

Appendix Table S6. Yeast strains, plasmids and reagents

Antibodies	SOURCE	IDENTIFIER
Goat anti mouse IgG-Peroxidase	Sigma	Cat. # A4416; RRID:AB_258167
Goat anti rabbit IgG-Peroxidase	Sigma	Cat. # A0545; RRID:AB_257896
Mouse monoclonal anti PGK1 (22C5D8)	Invitrogen	Cat. # 459250; RRID:AB_2532235
Mouse monoclonal anti GFP (IgG1K, clones 7.1 and 13.1)	Roche Diagnostics	Cat. # 11814460001; RRID:AB_390913
Mouse monoclonal anti FLAG M2	Sigma	Cat. # F3165; RRID:AB_259529
Mouse monoclonal anti HA (12CA5)	Abcam	Homemade hybridoma supernatant, lot 20081201
Mouse monoclonal anti ubiquitin (P4D1)	Santa Cruz	Cat. # sc-8017; RRID:AB_628423
Mouse monoclonal anti Ypk1 phospho-T662	Gift from Loewith lab	(Berchtold et al., 2012)
Goat polyclonal anti-Ypk1	Santa Cruz	Cat. # yl15; RRID:AB_793265
Rabbit polyclonal anti-Orm2, generated against N-terminal peptide MIDRTKNESPAFEESPLTP	Gift from Howard Riezman	This study
Rabbit polyclonal anti-Lcb1	Gift from Teresa Dunn	(Gable, Slife et al., 2000)
Rabbit polyclonal anti-Lcb2	Gift from Teresa Dunn	(Gable et al., 2000)
Rabbit polyclonal anti-Tul1	Gift from Peter Espenshade	(Tong et al., 2014)
Rabbit polyclonal anti-Ubx3	Gift from Peter Espenshade	(Tong et al., 2014)
Rabbit polyclonal anti-Dsc2	Gift from Peter Espenshade	(Tong et al., 2014)
Rabbit polyclonal anti-Dsc3	Gift from Peter Espenshade	(Tong et al., 2014)
**Chemicals, Peptides, and Recombinant Proteins**	**SOURCE**	**IDENTIFIER**
[13C6,15N2]-L-lysine	Sigma	Cat. # 608041
anti-Flag magnetic beads M2	Sigma	Cat. # M8823; RRID:AB_2637089
3xFLAG peptide	Sigma	Cat. # F4799
Phos-tag acrylamide	Wako	304-93521
MG-132	Sigma	Cat. # M7449
Cycloheximide	Sigma	Cat. # C7698
**Commercial Assays**	**SOURCE**	**IDENTIFIER**
TAQman gene expression assay for ORM2	Thermo Scientific	Cat. # Sc04149509_s1
TAQman gene expression assay for PGK1	Thermo Scientific	Cat. # Sc04104844_s1
RNeasy Mini kit	Qiagen	Cat. # 74104
Revert Aid First strand cDNA synthesis kit	Thermo	Cat. # K1622
**Yeast strains:**	**SOURCE**	**IDENTIFIER**

Y7092 wildtype (*MATalpha can1::STE2pr-Sp_HIS5 lyp1Δ leu2Δ ura3Δ his3Δ met15Δ cyh2)*	(Tong et al., 2001)	Y7092
Y7092 *vps4::NatR +p(CEN; LEU2; YPT53pr-URA3)*	this study	OSY162
BY4743 *Mat.A/alpha xxx::KanMX4/XXX*	(Buser et al., 2016); Peter lab	BY4743 deletion strain collection
SEY6210 wildtype (*MATalpha leu2-3, 112 ura3-52 his3-200 trp1-901 lys2-801 suc2-9)*	(Robinson, Klionsky et al., 1988)	SEY6210
SEY6210.1 wildtype (*MATa leu2-3, 112 ura3-52 his3-200 trp1-901 lys2-801 suc2-9)*	(Robinson et al., 1988)	SEY6210.1
SEY6210 *vps4::TRP1*	(Babst et al., 1997)	MBY3
SEY6210.1 *vps4::TRP1*	(Babst et al., 1997)	MBY4
SEY6210.1 *tul1::HIS3*	this study	SSY17
SEY6210.1 *vps4::TRP1, tul1::HIS3*	this study	SSY31
SEY6210.1 *orm1::HIS3*	this study	OSY510
SEY6210 *orm1::HIS3*	this study; courtesy of C. Stefan	YSC570
SEY6210 *orm2::TRP1*	this study; courtesy of C. Stefan	YSC580
SEY6210.1 *orm2::TRP1*	this study	YWY005
SEY6210.1 *orm2::TRP1, atg8::HIS3*	this study; (Müller, Schmidt et al., 2015)	YWY003
SEY6210 *orm2::TRP1, pdr5::HIS3*	this study	YWY006
SEY6210.1 *orm2::TRP1, hrd1::HIS3*	this study	YWY012
SEY6210.1 *orm2::TRP1, doa10::HIS3*	this study	YWY014
SEY6210.1 *orm2::TRP1, doa10::HIS3, hrd1::HIS3*	this study	YWY020
SEY6210.1 *orm2::TRP1, tul1::HIS3*	this study	YWY027
SEY6210.1 *vps4::TRP1, orm2::TRP1, tul1::HIS3*	this study	YWY029
SEY6210.1 *orm2::TRP1, pep4::LEU2*	this study; (Bankaitis & Emr, 1986)	YWY032
SEY6210.1 *ubx3::HIS3*	this study	YWY042
SEY6210.1 *dcs2::HIS3*	this study	YWY045
SEY6210.1 *dsc3::HIS3*	this study	YWY046
SEY6210 *orm2::TRP1, ubx3::HIS3*	this study	YWY047
SEY6210.1 *vps4::TRP1, ubx3::HIS3*	this study	YWY048
SEY6210 *orm2::TRP1, dsc3::HIS3*	this study	YWY055
SEY6210.1 *orm2::TRP1, dsc2::HIS3*	this study	YWY056
SEY6210 *tul1::HIS3, pep4::LEU2*	this study; (Bankaitis & Emr, 1986)	YWY072
SEY6210.1 *orm2::TRP1, doa10::HIS3, hrd1::HIS3, asi1::KanMX4*	this study	YWY129
SEY6210.1 *vps4::TRP1, orm2::TRP1*	this study	VBY51
SEY6210 *pep4::LEU2*	this study; (Bankaitis & Emr, 1986)	OSY454
SEY6210.1 *orm2::TRP1, tul1::HIS3, pdr5::HIS3*	this study	OSY721
SEY6210.1 *orm2::TRP1, YIP(TRP1)-TPI1pr-pre(KAR2)-dsRedExpress2-HDEL*	this study	OSY724
SEY6210.1 *orm2::TRP1, tul1::HIS3, YIP(TRP1)-TPI1pr-pre(KAR2)-dsRedExpress2-HDEL*	this study	OSY725
SEY6210 *orm2::TRP1, tul1::HIS3, Vps4-3xHA-mCherry::TRP1*	this study; (Adell et al., 2017)	OSY727
SEY6210 *orm1::HIS3, tul1::HIS3*	this study	OSY731
SEY6210.1 *orm1::HIS3, tul1::HIS3*	this study	OSY732
SEY6210.1 *orm2::TRP1, tul1::HIS3, YIP(URA3)-SEC7pr-SEC7-6xmCherry*	this study; (Day et al., 2018)	OSY738
SEY6210.1 *tul1::HIS3; Vps4-3xHA-eGFP::TRP1, YIP(URA3)-SEC7pr-SEC7-6xmCherry*	this study; (Day et al., 2018)	OSY751
SEY6210.1 *orm2::TRP1, vld1::HIS3*	this study	MWY010

SEY6210.1 *orm2::TRP1, gld1::HIS3*	this study	MWY013
SEY6210.1 *cdc48-3, orm2::TRP1, pdr5::HIS3*	this study; (Moir, Stewart et al., 1982)	OSY715
SEY6210.1 *cdc48-3, orm2::TRP1*	this study; (Moir et al., 1982)	OSY720
YP102 wildtype (*ura3-52 lys2-801 ade2-101 his3Δ200 leu2-Δ1)*	(Ghislain et al., 1993)	OSY343
YP102 *cim3-1*	(Ghislain et al., 1993)	DTY551
BY4742 wildtype (*MATalpha his3Δ 1; leu2Δ 0; lys2Δ 0; ura3Δ)*	(Brachmann, Davies et al., 1998)	BY4742
BY4742 *tsc3::KanMX4*	Open Biosystems	OSY737
SEY6210.1xBY4742 *tsc3::KanMX4; tul1::HIS3; trp1Δ*	this study	OSY746
TB50 *MATalpha; tor1-1, avo3Δ1274-1430::hphMX6*	(Gaubitz et al., 2015)	MPr8
SEY6210.1 *orm2::TRP1 SEC13-WT -T(ADH1)::HIS3*	this study	OSY809
SEY6210.1 *orm2::TRP1 sec13-4 -T(ADH1)::HIS3*	this study	OSY810
SEY6210.1 *orm2::TRP1, sec13-4 -T(ADH1)::HIS3; YIP(TRP1)-P(TPI1)-pre(Kar2)-dsRedExpress2-HDEL*	this study	OSY821
SEY6210.1 *orm2::TRP1, SEC13-WT -T(ADH1)::HIS3; YIP(TRP1)-P(TPI1)-pre(Kar2)-dsRedExpress2-HDEL*	this study	OSY826
SEY6210 *tul1::HIS3; orm2::TRP1; SEC13-WT -T(ADH1)::HIS3; YIP(TRP1)-P(TPI1)-pre(Kar2)-dsRedExpress2-HDEL*	this study	OSY828
SEY6210.1 *tul1::HIS3; orm2::TRP1; sec13-4 -T(ADH1)::HIS3; YIP(TRP1)-P(TPI1)-pre(Kar2)-dsRedExpress2-HDEL*	this study	OSY829
SEY6210.1 *sec13-4 -T(ADH1)::HIS3; YIP(TRP1)-P(TPI1)-pre(Kar2)-dsRedExpress2-HDEL*	this study	OSY823
SEY6210.1 *SEC13-WT -T(ADH1)::HIS3; YIP(TRP1)-P(TPI1)-pre(Kar2)-dsRedExpress2-HDEL*	this study	OSY824
**Primer for gene knock out / knock in**	**Used for strain:**	**Primer name:**
CTAATTCAGGCAAAAAAGGAAAGTCCTACAGCAAAAGA<br>GGCAAAAGTACAGTCGGATCCCCGGGTTAATTAA	SSY17	TUL1_KO_F
TACAAGGTCGATTTTACCTAACAGGGTGCTTGTAATAT<br>TGGAATTCGAGCTCGTTTAAAC	SSY17	TUL1_KO_R
CCTCATCAATCGGCTTGACTGCGATTTAAACATACCCT<br>TTAAACCGGATCCCCGGGTTAATTAA	YWY042	UBX3-KO-F
CTTTAGAATAAACGTTTTGAGATGACTATTTTTGAAAT<br>TCCTTGAATTCGAGCTCGTTTAAAC	YWY042	UBX3-KO-R
GGGAGAGAAAGTCAACGACATAAAAAGCAAACACAATA<br>GTCTACAAATACGGATCCCCGGGTTAATTAA	YWY045	DSC2-KO-F
CCGTTGCTATGTTTATTTGTTTATGTAGGTATATGCTG<br>ATATAAAGAATTCGAGCTCGTTTAAAC	YWY045	DSC2-KO-R
CCAAGCAAAGAGAGAGGTGAAAGGCAGCATACATTGAT<br>AAAATTCGGATCCCCGGGTTAATTAA	YWY046	DSC3-KO-F
CATTATTGTACATGAAAACGACATCTAATCGCCTCACT<br>AGAATTCGAGCTCGTTTAAAC	YWY046	DSC3-KO-R
AATTGCAATTTGTAAGAGAAGGGGAGAAAGACAAAATA<br>ATAATCGGATCCCCGGGTTAATTAA	YWY012	HRD1-KO-F
GGCTGAGATTGATCAGACTCAACCGTGTTTTCATCTGT<br>AGGATCGCGAATTCGAGCTCGTTTAAAC	YWY012	HRD1-KO-R
GCCAAGAGTACCACTAATTGAATCAAAGAGACTAGAAG<br>TGTGACGGATCCCCGGGTTAATTAA	YWY014	DOA10-KO-F
CGTGGTTAATTCTGGAGTTGCTGACTGATCATAACCAT<br>AAATCGAATTCGAGCTCGTTTAAAC	YWY014	DOA10-KO-R
CTTAATTTTATCTGGGTTTTTTTCTTCTTTTTACAAAG<br>AAACTATGCTAAGAATCGGATCCCCGGGTTAATTAA	YWY129	ASI1-KO-F

CCCAAACGAAAAACCTCTTTTAGATACCATGCAAAAGTTCTTAAACTAGAATTCGAGCTCGTTTAAAC	YWY129	ASI1-KO-R
GGCTTAGATGACAAGCTAATACAGAAAGGGAAAGGAGCAGGGCATCGGAAGGAACGGATCCCCGGGTTAATTAA	MWY010	VLD1-KO-F
CCGTTCTTTTCATTTTGGTAATTTTTTTTCTTTTCCTTTCTTGAATGAATTCGAGCTCGTTTAAAC	MWY010	VLD1-KO-R
CCATTAGCAGGCCAAAAGCAAACTAATCTATGTTTTCAATAAGGCCAATATAATACGGATCCCCGGGTTAATTAA	MWY013	GLD1-KO-F
GCCGATAACAGTAATATGCATTACCCCTTATGGACTACATATATATACTAGAATTCGAGCTCGTTTAAAC	MWY013	GLD1-KO-R
GCACCTTAGAAGGTCACAGCGATTGGG	OSY809 / OSY810	SEC13-F
GCCTGTTCTATTGAAGAAAAACAGTCTTATATATCTATTGTATATACGTCGATTATTTTTGTTCGCGAATTCGAGCTCGTTTAAAC	OSY809 / OSY810	SEC13utr-LG-R
**Primer for plasmid cloning**	**Used for plasmid(s):**	**Primer name:**
cgcTCTAGAGGGTCCCCGGCATTGAGG	pOS129; pVB17; pOS128; pOS143; pYW006 and all derivatives thereof	ORM2_XbaI_F
gcgGAATTCCCCTAGAGGCAAGATTGTAGCTGAAGCTGG	pOS129; pVB17; pOS128; pOS143; pYW006 and all derivatives thereof	ORM2_EcoRI_R
CCATTATAAAAACGCATAAGAAACAGTTTCATCATGGACTACAAAGACCATGACGG	pOS129	Prom(ORM2)-FLAG_F
CCGTCATGGTCTTTGTAGTCCATGATGAAACTGTTTCTTATGCGTTTTTATAATGG	pOS129	Prom(ORM2)-FLAG_R
CGATTACAAGGATGACGATGACAAGGGATCCATGATTGACCGCACTAAAAACGAATCTCC	pOS129	FLAG-ORM2_F
GGAGATTCGTTTTTAGTGCGGTCAATCATGGATCCCTTGTCATCGTCATCCTTGTAATCG	pOS129	FLAG-ORM2_R
CCAGTGACCGACCATAGGAGAAGACGGGCAGCCGCCGTAATATCACATGTGGAACAGGAAACC	pYW001; pYW008	ORM2_AAA_F
GGTTTCCTGTTCCACATGTGATATTACGGCGGCTGCCCGTCTTCTCCTATGGTCGGTCACTGG	pYW001; pYW008	ORM2_AAA_R
CCAGTGACCGACCATAGGAGAAGACGGGACGACGACGTAATATCACATGTGGAACAGGAAACC	pYW002; pYW009	ORM2_DDD_F
GGTTTCCTGTTCCACATGTGATATTACGTCGTCGTCCCGTCTTCTCCTATGGTCGGTCACTGG	pYW002; pYW009	ORM2_DDD_R
GGATCCATGATTGACCGCACTAGAAACGAATCTCCAGCTTTTG	pYW014	Orm2-K6R-F
CAAAAGCTGGAGATTCGTTTCTAGTGCGGTCAATCATGGATCC	pYW014	Orm2-K6R-R
CCAATGTGTCTAACCTGAGACCATTCCCTTCTCAAAGCAACAGAATATCCACTCCAGTGACC	pYW015	Orm2-K25R-K33R-F
GGTCACTGGAGTGGATATTCTGTTGCTTTGAGAAGGGAATGGTCTCAGGTTAGACACATTGG	pYW015	Orm2-K25R-K33R-R
CCCTGCTCAAGCCAACAAAATATCCGCTCCAGTGACCGACCATAGGAGAAGACGG	pOS148	ORM2_29-31-36A-F
CACTGGAGCGGATATTTTGTTGGCTTGAGCAGGGAATGGTTTCAGGTTAGACAC	pOS148	ORM2_29-31-36A-R
GAAGAGGCTCCGCTTGCCCCCAATGTGGCTAACCTGAAACCATTCCCTGCTCAAGCC	pOS148	ORM2_15-18-22A-F
CAGGTTAGCCACATTGGGGGCAAGCGGAGCCTCTTCAAAAGCTGGAGCTTCG	pOS148	ORM2_15-18-22A-R

CGAAGCTCCAGCTTTTGAAGAGTCTCCGCTTACC	pOS148	ORM2_S9A-F
GCTGGAGCTTCGTTTTTAGTGCGGTCAATCATGGATCC	pOS148	ORM2_S9A-R
cgcTCTAGAGCGCGTAGGGCCGCCAGCG	pOS127	ORM1_XbaI_F
cgcGAATTCGAAGCAGTACGTGAAATAGTGC	pOS127	ORM1-EcoRI_R
cgcCTCGAGGCGCGTAGGGCCGCCAGCG	pYW005	ORM1_XhoI_F
cgcGAGCTCGAAGCAGTACGTGAAATAGTGC	pYW005	ORM1-SacI_R
cgcgagctcCCCTGACGTCCTGATCGTGCCC	pOS166	Sac1-SacI-F2
cgcgcggccgcGGGCGAACGCCTTCATGTATAGTAGCC	pOS166	Sac1-NotI-R2
cgcGAGCTCGTACTGTCATCATCGTTTGC	pYW032	TUL1-SacI-F
gcgACTAGTGGAGTTGGAGCGATTTAG	pYW032	TUL1-SpeI-R
cgcGAGCTCCGGAAAGTTCATACAGTTGC	pYW052	SacI-Ubx3-F
cgcACTAGTGTGAGTTAGATCAATTTTGGC	pYW052	SpeI-Ubx3-R
GTACGAGCTCGCAGTTATACCGCTATAAGCAATTG	pSM52	A_Sec61-mCherry_FWD
CTCACGTTAATTAACCCGGGGATCCGCATCAAATCAGAAAATCCTGGAAC	pSM52	A_Sec61-mCherry_REV
GATGCGGATCCCCGGGTTAATTAACGTGAGCAAGGGCGAGGAGGATAAC	pSM52	B_Sec61-mCherry_FWD
TCACTAGTGATCTTACTTGTACAGCTCGTCCATG	pSM52	B_Sec61-mCherry_REV
CGCAAGCTTGCACCTTAGAAGGTCACAGCGATTGGG	pOS239; pOS241	SEC13-HindIII-F
CGCGGCGCGCCTCCCTTCAAATTCGTCGATAGACTCATTTGCATTC	pOS239; pOS241	SEC13-AscI-R
CCAGTTGGTCTTTGTCAG**AC**AATGTACTAGCTCTTTCCGGTGGCG	pOS241	sec13-4_F
GAGCTAGTACATTGTCTGACAAAGACCAACTGGCTCTCCATAAAACATC	pOS241	sec13-4_R
**Plasmids**	**SOURCE**	**IDENTIFIER**
empty centromer vector *HIS3*	(Sikorski & Hieter, 1989)	pRS413
empty centromer vector *TRP1*	(Sikorski & Hieter, 1989)	pRS414
empty centromer vector *LEU2*	(Sikorski & Hieter, 1989)	pRS415
empty centromer vector *URA3*	(Sikorski & Hieter, 1989)	pRS416
pRS415 - *VPS4*	(Müller et al., 2015)	pOS014
pRS416 - *3xHA-ORM2* (endogenous 5' and 3')	this study	pOS128
pRS416 - *3xFLAG-ORM2* (endogenous 5' and 3')	this study	pOS129
pRS416 - *3xFLAG-ORM2*^{S9A,S15A,T18A,S22A,S29A,S31A,T36A} (endogenous 5' and 3')	this study	pOS148
pRS415 - *HA-SAC1* (endogenous 5' and 3')	this study	pOS166
pRS415 - *GFP-ORM2* (endogenous 5' and 3')	this study	pOS173
pRS416 - *GFP-ORM2*^{K25R,K33R,S46A,S47A,S48A} (endogenous 5' and 3')	this study	pOS242
pRS416 - *GFP-ORM2*^{K25R,K33R,S46D,S47D,S48D} (endogenous 5' and 3')	this study	pOS243
pFA6a - *SEC13-WT-C-term. fragment-T(ADH1)::HIS3* template plasmid for generation of OSY809 (SEC13 WT) and derivatives thereof	this study	pOS239
pFA6a - sec*13-4-C-term. fragment-T(ADH1)::HIS3* template plasmid for generation of OSY810 (SEC13 WT) and derivatives thereof	this study	pOS241
pRS416 - *ORM2* (endogenous 5' and 3')	this study	pVB17
pRS416 - *3xFLAG-ORM2*^{S46A,S47A,S48A} (endogenous 5' and 3')	this study	pYW001

pRS416 - *3xFLAG-ORM2*^{S46D,S47D,S48D} (endogenous 5' and 3')	this study	pYW002
pRS416 - *GFP-ORM1* (endogenous 5' and 3')	this study	pYW005
pRS416 - *GFP-ORM2* (endogenous 5' and 3')	this study	pYW006
pRS416 - *GFP-ORM2*^{S46A,S47A,S48A} (endogenous 5' and 3')	this study	pYW008
pRS416 - *GFP-ORM2*^{S46D,S47D,S48D} (endogenous 5' and 3')	this study	pYW009
pRS415- ^{ADH1}*Ub-P-GFP*	this study; (Müller et al., 2015)	pYW010
pRS416 - *3xFLAG-ORM2*^{K6R} (endogenous 5' and 3')	this study	pYW014
pRS416 - *3xFLAG-ORM2*^{K25R,K33R} (endogenous 5' and 3')	this study	pYW015
pRS416 - *3xFLAG-ORM2*^{K25R,K33R,S46A,S47A,S48A} (endogenous 5' and 3')	this study	pYW026
pRS416 - *3xFLAG-ORM2*^{K25R,K33R,S46D,S47D,S48D} (endogenous 5' and 3')	this study	pYW027
pRS416 - *GFP-ORM2*^{K25R,K33R} (endogenous 5' and 3')	this study	pYW028
pRS416 - *3xFLAG-ORM2*^{K25R} (endogenous 5' and 3')	this study	pYW030
pRS416 - *3xFLAG-ORM2*^{K33R} (endogenous 5' and 3')	this study	pYW031
pRS415 - *TUL1 (endogenous 5' and 3')*	this study	pYW032
pRS415 - *UBX3 (endogenous 5' and 3')*	this study	pYW052
pRS415 - *SEC61-eGFP (endogenous 5' and 3')*	this study	pSM52
pRS415 - *VPS4-3xHA-eGFP* (endogenous 3' and 5')	this study; (Adell et al., 2017)	pSS37
pRS415 - *P*^{TDH3}*-mCherry-CPS1(2-270)*	(Adell et al., 2017)	pC29
YIP(URA3)-SEC7pr-SEC7-6xmCherry	(Day et al., 2018)	YIplac211-SEC7-mCherry2Bx6; RRID:Addgene_105267
YIP(TRP1)-TPI1pr-pre(KAR2)-dsRedExpress2-HDEL	Benjamin Glick, unpublished	YIPlac204TKC-DsRed-Express2-HDEL; RRID:Addgene_21770

**Software and Algorithms**	**SOURCE**	**IDENTIFIER**
Photoshop CS5	Adobe	Version 12.0.4x64; RRID:SCR_014199
Illustrator CS5.1	Adobe	Version 15.1.0; RRID:SCR_010279
Proteome Discoverer 2.2	Thermo Scientific	Version 2.2; RRID:SCR_014477
Excel for Mac	Microsoft	Version 16.16.2; RRID:SCR_016137
ImageJ2	(Rueden et al., 2017)	Version 2.0.0-rc49/1.51h; RRID:SCR_003070
Fiji	(Schinderlin et al., 2012)	Version 1.0
Huygens Professional	Scientific Volumes Imaging	Version 18.19
Visi View	Visitron	Version 2.1.4
LasX	Leica	Version 3.5.2.18963
