# Supplementary material for: Endosome and Golgi‐associated degradation (EGAD) of membrane proteins regulates sphingolipid metabolism
Source: EMBO J. 2019 May 27;38(15):e101433. doi: 10.15252/embj.2018101433 (PMC6669922; doi:10.15252/embj.2018101433)
Supplement: Supplementary file 2 — Expanded View Figures PDF [file EMBJ-38-e101433-s002.pdf]

Expanded View Figures

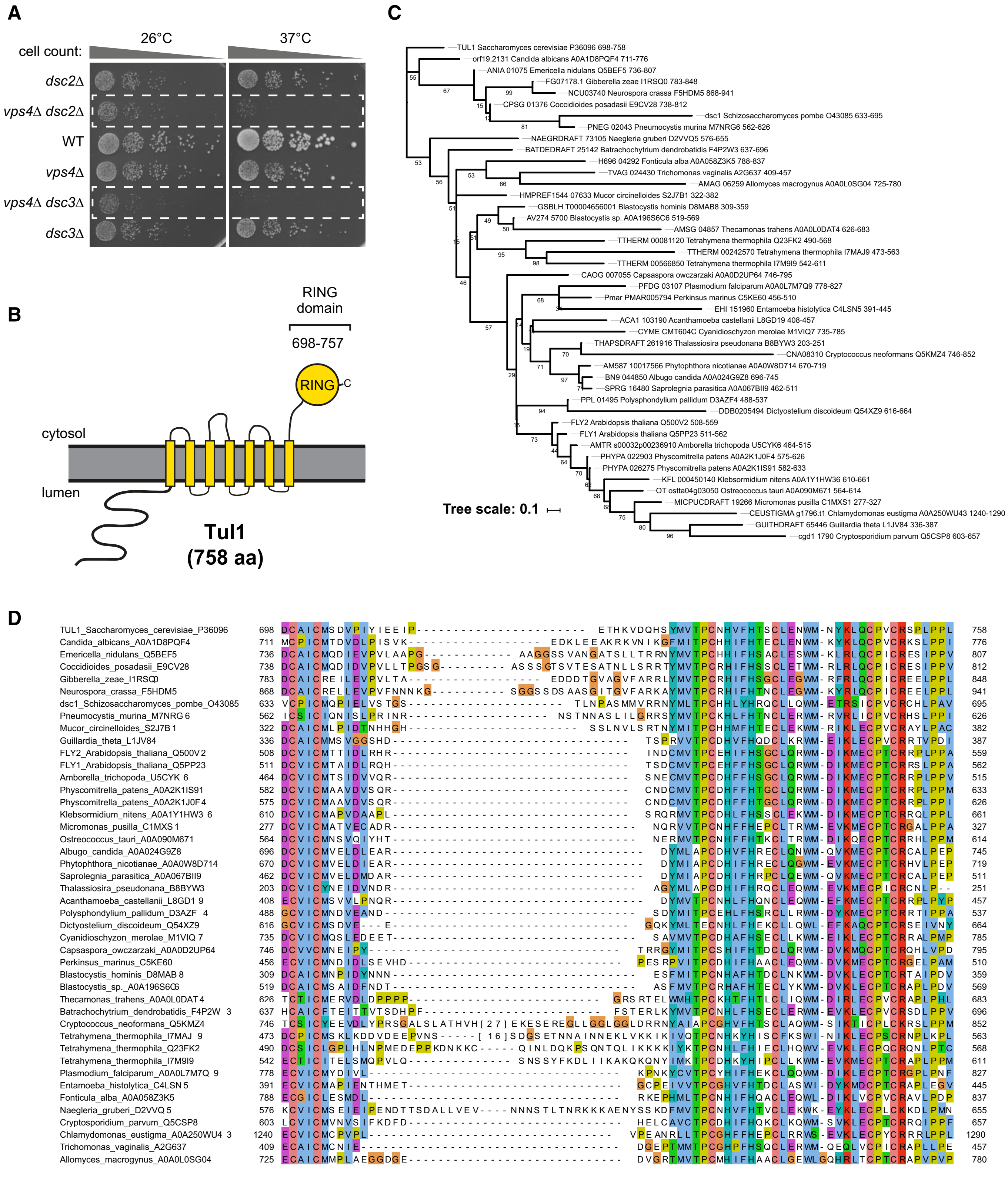

Figure EV1.

 © 2019 The Authors

Figure EV1. Genetic and bioinformatic analysis (related to Fig 1).

A Equal amounts of WT cells and indicated single or double mutants in serial dilutions were incubated on agar plates at the indicated temperatures.
B Schematic representation of the topology of Tul1.
C Phylogenetic tree of selected Tul1 orthologs. Ultrafast bootstrap (UFboot) values are indicated on the branches.
D Multiple alignment of the Tul1 RING finger domain. UniProt sequence accessions are provided after the species name. For two sequences (Cryptococcus_neoformans_Q5KMZ4, Tetrahymena_thermophila_I7MAJ9), a long insertion was replaced by the number of deleted residues in brackets.

Figure EV2. Functional link between the Dsc and the SPOTS complex (related to Fig 2).

A Gene Ontology (GO) analysis for cellular processes and components of 76 proteins that were stabilized in *tul1Δ pep4Δ* mutants relative to *pep4Δ* cells (Appendix Tables S4 and S5). Only GO terms with more than twofold enrichment over the genome frequency are shown. Red GO terms were reported with false discovery rate (FDR) below 0.1; black GO terms with FDR above 0.1.
B Tul1 genetic interaction profile similarities (using http://thecellmap.org) (Costanzo *et al*, 2016) (Pearson correlation coefficient, PCC = 0.10).
C Equal amounts of WT cells and indicated single or double mutants in serial dilutions were incubated on agar plates at the indicated temperatures. The dashed box indicates the *tul1Δ tsc3Δ* double mutant.
D Negative genetic interaction network of *tul1Δ* (Costanzo *et al*, 2016) (PCC = −0.3).
E Schematic presentation of Orm1 and Orm2 with Ypk1-dependent phospho-sites and the ubiquitination sites in Orm2 that were identified here.
F SDS–PAGE and Western blot of total cell lysates of the indicated strains demonstrating specificity of the anti-Orm2 antibody.
G Quantification of *ORM2* mRNA normalized to stable *PGK1* mRNA from WT cells and *tul1Δ* mutants by qPCR (n = 4). Data are presented as mean fold change from WT ± standard deviation. Statistical significance was assessed by Student's *t*-test.
H SDS–PAGE and Western blot analysis of total cell lysates of WT cells or the indicated mutants with the indicated antibodies.

© 2019 The Authors

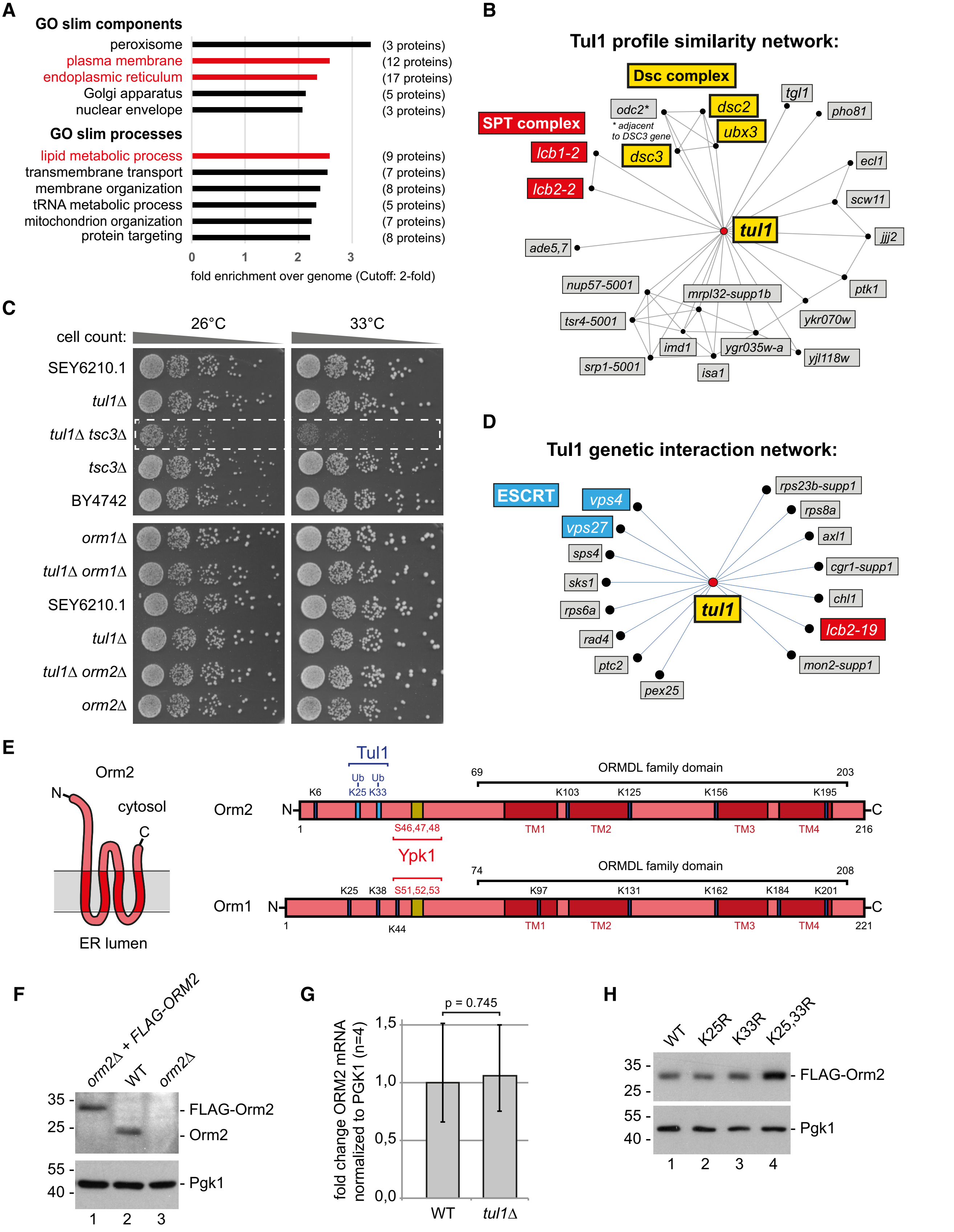

Figure EV2.

 © 2019 The Authors

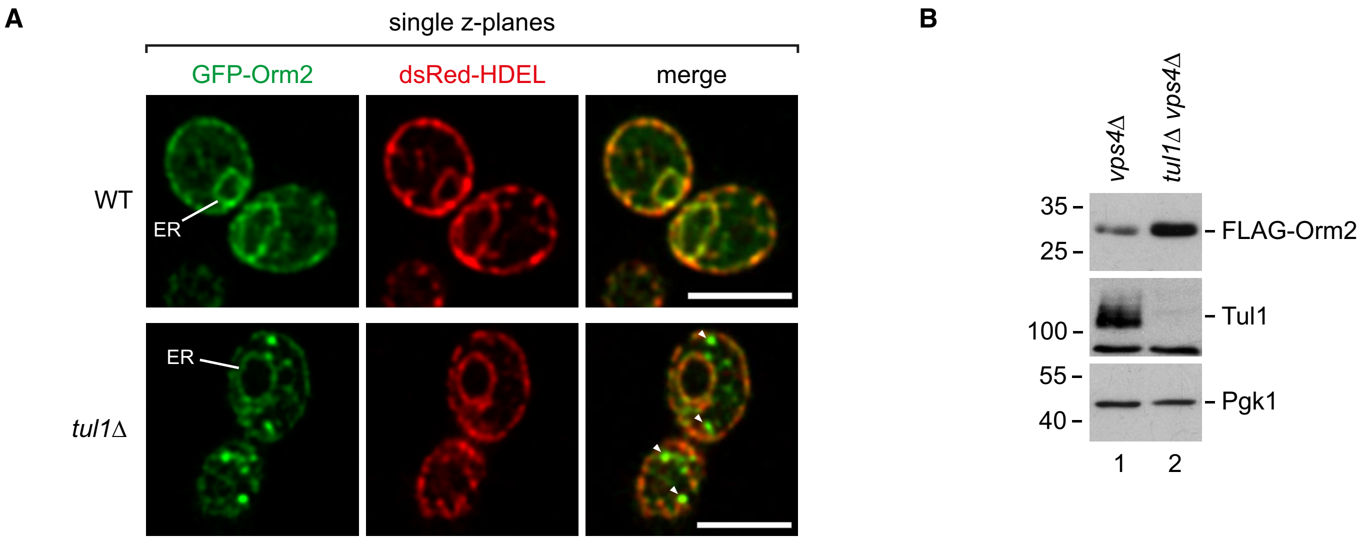

Figure EV3. Confocal microscopy of Orm2 and analysis of Orm2 protein levels (related to Fig 3).

A Single z planes from life cell confocal fluorescence microscopy of WT cells and *tul1Δ* mutants expressing GFP-Orm2 and dsRed-HDEL. Scale bars 5 μm.

B SDS–PAGE and Western blot analysis of total cell lysates of the indicated mutants with the indicated antibodies.

Figure EV4. Analysis of Orm1/2 degradation (related to Fig 4).

Cells were treated with cycloheximide (CHX) to block protein synthesis for the indicated times before cells were lysed. Total cell lysates were analyzed by SDS–PAGE and Western blot (A-C,G) with the indicated antibodies.

A, B WT cells or the indicated mutants expressing FLAG-Orm2.

C *pdr5Δ* mutants expressing FLAG-Orm2 and Ub-GFP were incubated with MG-132 or vehicle (DMSO) 10 min prior to the addition of CHX.

D Live-cell epifluorescence and phase-contrast microscopy of *pep4Δ* mutants expressing GFP-Orm2.

E SDS–PAGE and Western blot analysis of total cell lysates from WT and *sec13-4* cells incubated at the indicated temperatures.

F Epifluorescence and phase-contrast microscopy of *sec13-4* mutants expressing dsRed-HDEL and GFP-Orm2 (upper panels) or Vps4-eGFP (lower panels) were incubated at 26°C or shifted for 90 min to the non-permissive temperature (37°C).

G WT cells or *tul1Δ* mutants expressing HA-Orm1.

H Epifluorescence and phase-contrast microscopy of living WT cells and *tul1Δ* mutants expressing GFP-Orm1.

Data information: scale bars = 5 μm.

© 2019 The Authors

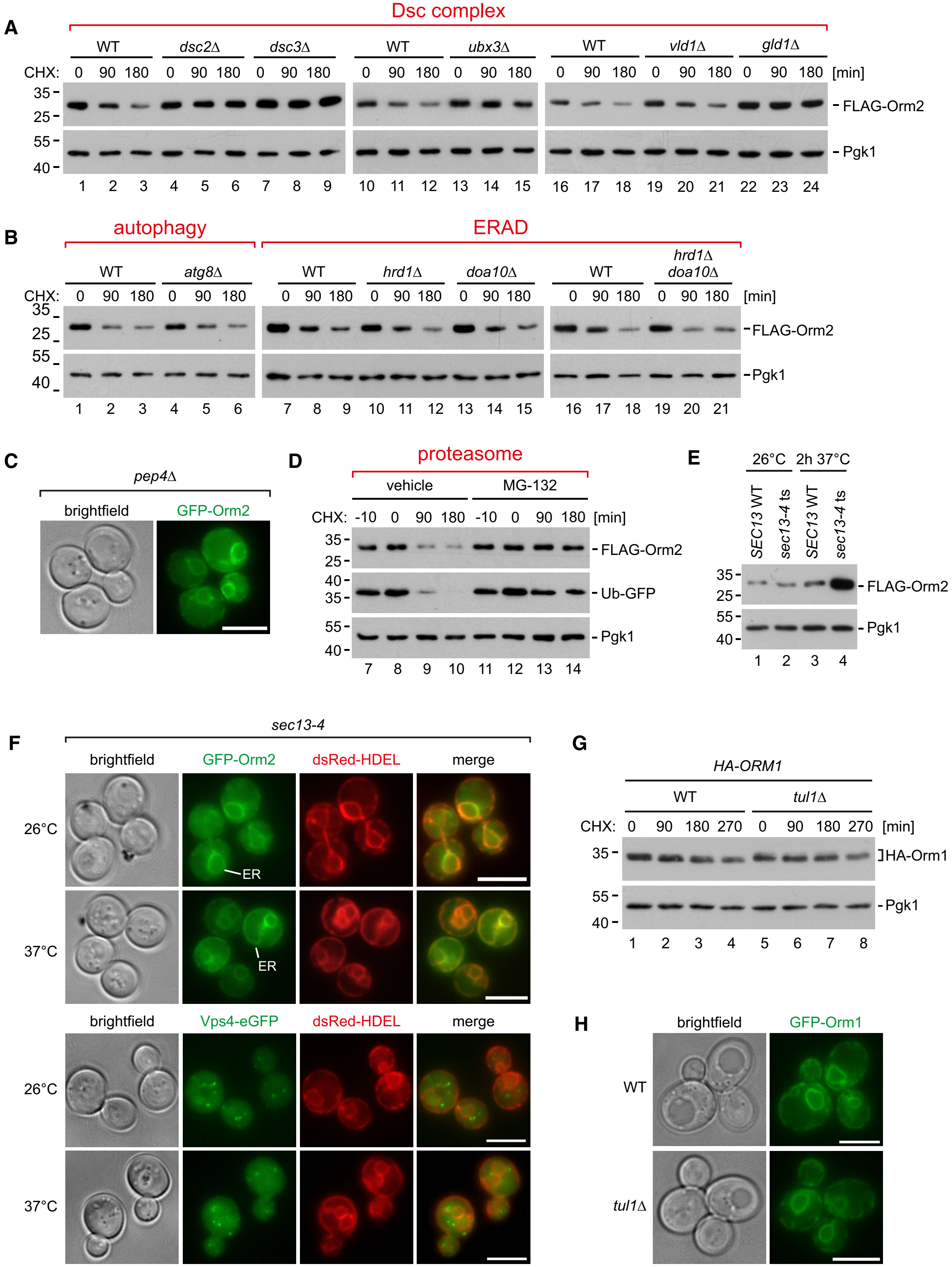

Figure EV4.

 © 2019 The Authors

Figure EV5. Analysis of Orm1/2 phosphorylation, protein levels, and localization (related to Figs 6 and 7).

A SDS–PAGE or Phos-tag SDS–PAGE and Western blot analysis of total cell lysates from WT cells and *tul1Δ* mutants expressing FLAG-Orm2. Right panel: densitometric quantification of phosphorylated forms of FLAG-Orm2 or FLAG-Orm2-K25,33R from three independent experiments (mean ± standard deviation).

B SDS–PAGE or Phos-tag SDS–PAGE and Western blot analysis of total cell lysates from WT cells and *tul1Δ* mutants expressing HA-Orm1.

C Epifluorescence and phase-contrast microscopy of living *cdc48*-3 mutants expressing GFP-Orm2-3A (green) incubated for 5 h at the (non-)permissive temperature (26°C, 37°C).

D Left panel: densitometric quantification of FLAG-Orm2 protein levels (in WT and *tul1*Δ cells), and FLAG-Orm2-K25,33R or FLAG-Orm2-3A protein levels from at least three independent experiments. Right panel: densitometric quantification of FLAG-Orm2-3A protein levels (in WT and *tul1*Δ cells). Mean ± standard deviation normalized to the respective control (set to 1).

E Epifluorescence and phase-contrast microscopy of cells expressing dsRed-HDEL (red) and GFP-Orm2-K25,33R-3A or GFP-Orm2-K25,33R-3D (green). Arrowheads point to GFP-Orm2 accumulations outside the endoplasmic reticulum (ER).

Data information: scale bars = 5 μm.

© 2019 The Authors

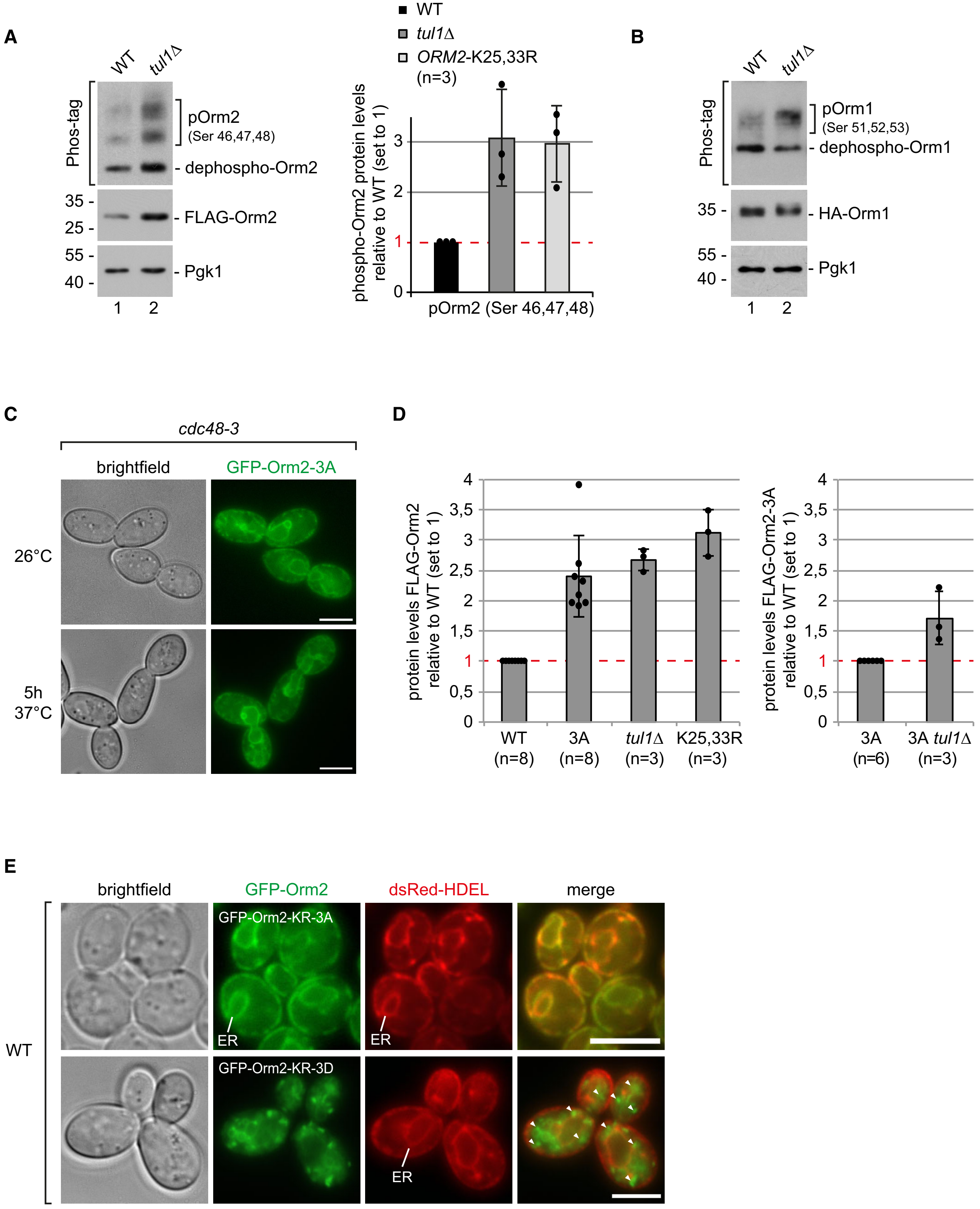

Figure EV5.

 © 2019 The Authors
